# Direct Arylation of Benzo[*b*]furan and Other Benzo-Fused Heterocycles

**DOI:** 10.1002/ejoc.201403125

**Published:** 2014-11-17

**Authors:** Toan Dao-Huy, Maximilian Haider, Fabian Glatz, Michael Schnürch, Marko D Mihovilovic

**Affiliations:** [a] Institute of Applied Synthetic Chemistry, Vienna University of Technology Getreidemarkt 9/163-OC, 1060 Vienna, Austria E-mail: michael.schnuerch@tuwien.ac.at http://www.ias.tuwien.ac.at/staff/3129565/

**Keywords:** Synthetic methods, Arylation, Fused-ring systems, C–H activation, Palladium, Regioselectivity, Concerted metal deprotonation

## Abstract

The direct arylation of benzo[*b*]furan, benzo[*b*]thiophene, and indole has been studied by using aromatic bromides as the aryl source. The protocol employing common reagents and a Pd catalyst has led to the regioselective arylation of these heterocycles at the 2-position. A range of functional groups were tolerated, providing quick access to a variety of arylated benzo-fused heterocycles that would be accessible more elaborately using classical synthetic strategies. This is the first systematic study of the direct arylation of benzo[*b*]furan.

## Introduction

**Figure 1:**
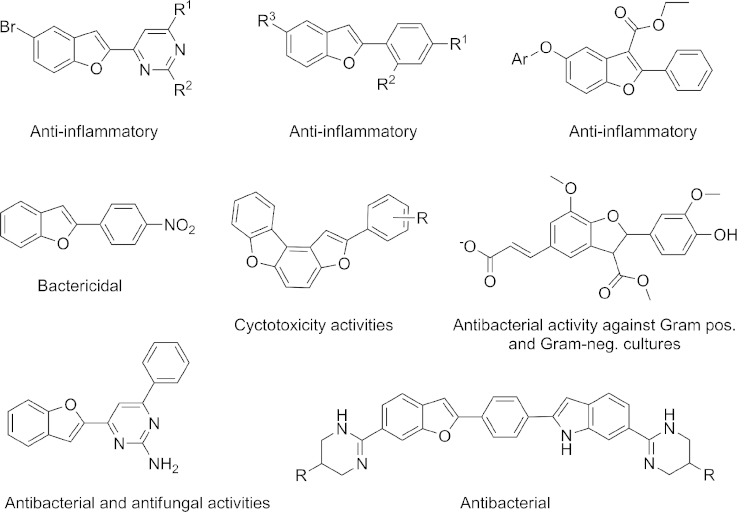
Bioactive benzo[*b*]furan derivatives.

2-Arylbenzo[*b*]furan derivatives are found frequently in natural products and synthetic compounds with high biological activity. For example, neolignins containing benzo[*b*]furan derivatives have been reported to have good anti-inflammatory properties.[1] Some representatives of this heterocyclic system are also anti-bacterial or potential anti-cancer agents (Figure 1).[2] Consequently, facile synthetic approaches to such compounds are of critical importance to further assess and refine the potential of this structural class as bioactive entities.

For decades, bi(hetero)aryl motifs, including arylated benzo[*b*]furans, have been synthesized by de novo synthesis using cyclization strategies.[3] This changed with the development of metal-catalyzed cross-coupling reactions, which were then the method of choice for the synthesis of such systems for many years.[4] In recent times, another method has started to complement and even substitute cross-coupling chemistry, namely direct arylation methods, often referred to as C–H activation reactions.[5] The advantage of this type of transformation is the absence of pre-functionalized coupling partners. Therefore, in the C–C bond-forming step, either the organometallic species (preferably) or the (pseudo)halide component can be substituted by a reactive (i.e., activated) C–H bond instead. This streamlines the synthesis and increases the efficiency considerably, because pre-functionalization (often achieved within multi-step sequences) of substrate(s) is no longer required. Hence, avoiding such pre-functionalization shortens synthetic pathways significantly, which is more effiecient in terms of time, resources, and energy. As a consequence, the environmental burden is reduced. All these factors often contribute to more economical processes as well.[6] Therefore, many research groups, including ours, have focused attention on the development of new methods for the direct arylation of heterocycles.[7]

Heterocycles are especially suitable for C–H activation chemistry because C–H bonds differ significantly in reactivity due to the presence of one or more heteroatoms. To date, series of electron-rich benzo-fused heterocycles have been activated successfully, often by using palladium species to catalyze the direct arylation.[8]–[12]

Interestingly, the direct arylation of benzo[*b*]furan has been largely neglected. Over the years, only a handful examples have been reported on this subject either with mediocre yields or under peculiar reaction conditions.[11] DeBoef and co-workers disclosed a protocol for the oxidative coupling of benzo[*b*]furan and benzene derivatives.[11a] However, benzene had to be used as solvent (>80 equiv. as a mixture with AcOH) and a complex catalytic system was required. Glorius and co-workers disclosed that *N*,*N*-diisopropylbenzamide can be oxidatively coupled with benzofuran under Rh catalysis, in this case with a 10-fold excess of benzofuran.[11e] Fagnou and co-workers reported the direct arylation of a series of heterocycles by using a common protocol;[11c] one example is the arylation of benzo[*b*]furan with *o*-bromotoluene and in this case the yield was low at 29 %. Also, Kappe[11d] and Bhanage[11b] and their co-workers each contributed one example of the arylation of benzo[*b*]furan with aryl halides. Kappe essentially followed the protocol of Fagnou and co-workers and obtained a yield of 50 % when benzo[*b*]furan was coupled with 3-bromoquinoline under microwave irradiation. Bhanage and co-workers treated benzo[*b*]furan with bromo- or iodobenzene using [Pd(tmhd)_2_] as catalyst (tmhd = 2,2,6,6-tetramethyl-3,5-heptanedione) in good yields of 65 and 83 %, respectively. Substituted aryl halides were not investigated. Mori et al. used iodobenzene, 4-iodoanisol, and ethyl 4-iodobenzoate as coupling partners in the direct arylation of benzo[*b*]furan.[10a] For iodobenzene, no isolated yield was reported, only an NMR yield of 41 % and with no further comments. The other two coupling partners applied gave yields of 41 and 53 %, respectively. A more detailed investigation of the direct arylation of benzo[*b*]furan was reported by Ohta in 1990.[13a] They used the simple [Pd(PPh_3_)_4_] as catalyst, aryl bromides as coupling partners and KOAc as base in DMAc at reflux. Of the 10 aryl bromides tested, only six gave the coupling product, but in low yields (typically ca. 20 %). The only exception was *o*-bromonitrobenzene, which gave 50 % of the arylation product. The same group reported the direct arylation of benzo[*b*]furan with chloropyrazines.[13b] The most C2 selective protocol was reported by Doucet and co-workers just recently.[11h] By using benzylsulfonyl chlorides as coupling partner in combination with [PdCl_2_(MeCN)_2_] and Li_2_CO_3_, they were able to arylate benzo[*b*]furan selectively at C2 in generally good yields (13 examples, 50–91 %). Even though this is the most selective protocol reported so far, the type of aryl donor applied can be considered as a certain drawback. Hence, there is still demand for a general, direct arylation protocol of benzo[*b*]furan using readily available aryl donors.

Comparing the aromaticity of benzo[*b*]furan with -thiophene or furan, the C2–C3 bond on benzo[*b*]furan behaves chemically more like an olefinic double bond than an aromatic system, as is the case in benzo[*b*]thiophene.[2d,^14^] Consequently, the arylation at C-2 of benzo[*b*]furan is not very selective. Normally, 2-, 3-, and bis-arylated products will be observed as a mixture causing problems for the isolation of the desired product.[13a] Therefore cyclization strategies are still often used to synthesize 2-arylbenzo[*b*]furans.[15] Naturally, this requires more steps than the direct arylation approach and moreover has the intrinsic possibility of encountering difficulties in the synthesis, namely intermediate stability and/or functional group tolerance.[16] On the other hand, oxidative direct arylation is another option, although many functional groups do not tolerate the required reaction conditions.[17]

Herein we describe our efforts to directly arylate benzo[*b*]furan by taking advantage of a protocol that can be used for benzo-fused heterocycles in general and also allows coupling with a wide range of coupling partners bearing different functional groups.

## Results and Discussion

We started our investigations by using the reaction conditions that we recently introduced for the successful C-5 arylation of *N*-protected thiazoleamines.[18] There we used palladium acetate [Pd(OAc)_2_] as catalyst without an additional ligand, potassium acetate (KOAc) as base and *N*,*N*-dimethylacetamide (DMAc) as solvent at 140 °C for 24 h. In the case of the arylation of thiazole, aryl iodides served as the aryl source, whereas for the arylation of benzo[*b*]furan we wanted to focus on cheaper and more readily available bromides. As a model reaction we investigated the coupling of 4-bromoanisole with benzo[*b*]furan.

Initially we started with a catalyst loading of 2 mol-% in the absence of any ligand, however, this gave only 16 % yield of the desired product (Table 1, entry 1). Screening of various catalyst loadings (1–10 mol-%; see the Supporting Information) revealed that 4 mol-% gave the highest but a still unsatisfactory yield (entry 2) as higher catalyst loadings led to increased byproduct formation (C-3 arylation, debromination, and homocoupling of 4-bromoanisole).

**Table 1:** Optimization of the arylation of benzo[*b*]furan[a]

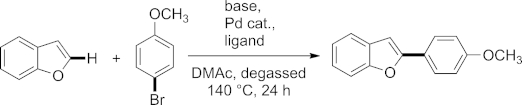				

Entry	Base	Pd cat.	Ligand	Yield[b] [%]
1	KOAc	Pd(OAc)_2_	–	16[c]
2	KOAc	Pd(OAc)_2_	–	22
3	KOAc	Pd(OAc)_2_	SPhos	45
4	KOAc	Pd(OAc)_2_	CPhos	40
5	KOAc	[Pd(PPh_3_)_2_Cl_2_]	SPhos	40
6	KOAc	[Pd(PPh_3_)_4_]	SPhos	44
7	CsOAc	Pd(OAc)_2_	SPhos	19
8	KOPiv	Pd(OAc)_2_	SPhos	65
9	CsOPiv	Pd(OAc)_2_	SPhos	72

[a] Reaction conditions: benzo[*b*]furan (1 equiv.), 4-bromoanisole (1.5 equiv.), base (1.5 equiv.), Pd cat. (4 mol-%), ligand (8 mol-%), 140 °C, 24 h, 0.5 m of substrate in solvent (degassed DMAc).

[b] The yield was determined by GC.

[c] 2 mol-% was used instead of 4 mol-% of Pd cat.

**Figure 2:**
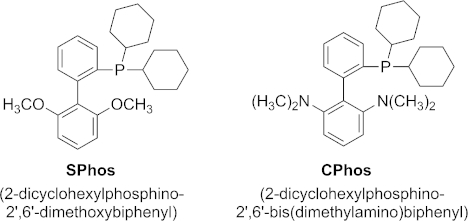
Structures of the phosphine ligands.

Because the addition of ligands has a significant influence on metal-catalyzed reactions (change in catalytic species and eventually oxidation state), we embarked on ligand screening with several commercially available ligands.[19] Because the addition of phosphine ligands changes the electronic and steric (vacant coordination sites blocked) environment of the catalytically active metal center, we chose to screen several commercially available phosphine ligands (see the Supporting Information). In this investigation only two systems (C-Phos and S-Phos, Figure 2) displayed improved yields compared with conditions without an additional ligand (40 and 45 % yields, respectively; entries 3 and 4). It has to be mentioned that none of the investigated conditions led to full conversion. Variation of the palladium catalyst precursor species also did not affect the reaction efficiency significantly (entries 5 and 6).

Although there is strong evidence that the reaction proceeds through a concerted metalation/deprotonation (CMD) pathway,[20a,20b] we chose a carboxylate base for our screening efforts. This modification was inspired by the work of Fagnou and co-workers, who reported that pivalic acid (in situ becoming potassium pivalate due to the presence of stoichiometric amounts of K_2_CO_3_) acts as a co-catalyst to promote C–H cleavage and phosphine dissociation, ultimately preventing catalyst inhibition by excess phosphine.[11c,20c] Speculating that the nature of the carboxylate base can also have a significant influence on the performance of a direct arylation process, we tested other carboxylate bases such as CsOAc, KOPiv, and CsOPiv.

**Scheme 1:**
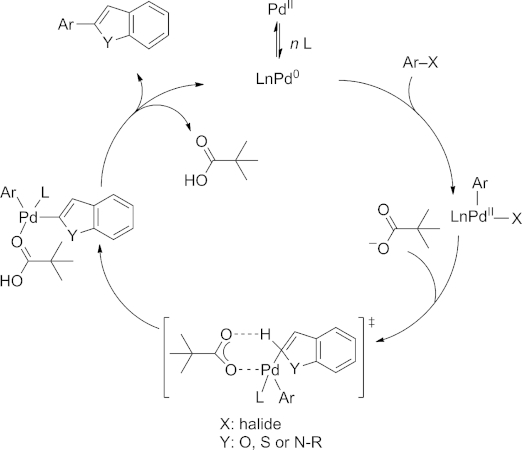
Proposed mechanism for the direct arylation of benzo-fused heterocycles using pivalate specie as co-catalyst.

The proposed mechanism of this reaction is shown in Scheme 1. The reaction starts with the oxidative addition of the aryl bromide to the palladium catalyst. Then a pivalate ion from caesium pivalate coordinates to the catalyst. In the subsequent step a concerted metalation deprotonation takes place, as depicted in the transition state. Finally, dissociation of pivalic acid and reductive elimination delivers the desired product and regenerates the catalytically active palladium species.

In contrast to the protocol by Fagnou and co-workers, we chose to use pivalate directly and use of either potassium pivalate (KOPiv) or caesium pivalate (CsOPiv) as base resulted in a significant increase in yield. Only when conducting the reactions in the presence of CsOPiv was full conversion observed (72 % yield, Table 1, entry 9). In addition, a minor counter-ion effect was observed favoring Cs^+^ over K^+^ (65 %, entry 8). In both cases, the 3-arylated isomer was detected as a minor byproduct (<5 %) by GC–MS. Having identified CsOPiv as the best-performing base we tested several N-based ligands in combination with this base, however, the conversions were significantly lower than those obtained with S-Phos (see the Supporting Information).

As a result, the following reaction conditions were used for to evaluate the substrate scope: Benzo[*b*]furan (1.0 equiv.) with aryl bromide (1.5 equiv.), CsOPiv (1.5 equiv.), Pd(OAc)_2_ (4 mol-%), and SPhos (8 mol-%) in degassed DMAc (0.5 m) at 140 °C for 24 h. Our results are summarized in Table 2. Benzo[*b*]furan was treated with a series of aryl bromides bearing various functional groups. In several cases, the bis-arylated benzo[*b*]furan was observed as the major byproduct by GC–MS, and is responsible for decreased yields of the desired product due to associated separation problems.

**Table 2:** Scope of the direct arylation of benzo[*b*]furan at the 2-position[a]

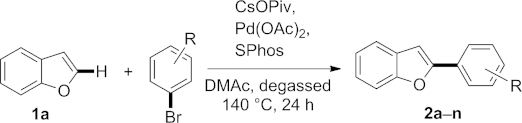				

Entry	R	Product	Ratio C2/C3/bis-aryl[b]	Yield[c] of 2[%]
1	H	**2a**	100:9:33	60
2	4-OH	**2b**	–	0
3	4-CH_3_	**2c**	100:6:29	54
4	4-OCH_3_	**2d**	100:5:n.o.	70
5	4-Cl	**2e**	100:20:12	57
6	4-F	**2f**	100:17:17	50
7	4-COOEt	**2k**	100:11:n.o.	45
8	4-CHO	**2l**	100:4:n.o.	44
9	4-CN	**2m**	100:5:42	34
10	4-NO_2_	**2n**	100:n.o.:n.o.	22
11	2-OCH_3_	**2g**	–	0
12	2-CH_3_	**2h**	100:20:16	40
13	2-Cl	**2i**	100:22:26	43
14	3-Cl	**2j**	100:18:32	50

[a] Reaction conditions: benzo[*b*]furan (1 equiv.), aryl bromide (1.5 equiv.), CsOPiv (1.5 equiv.), Pd(OAc)_2_ (4 mol-%), SPhos (8 mol-%), 140 °C, 24 h, 0.5 m of substrate in solvent (degassed DMAc).

[b] n.o.: not observed.

[c] Isolated yields.

To investigate the electronic effects of substituents on the aryl bromide only *para*-substituted derivatives were subjected to the established reaction conditions to eliminate steric interactions (Table 2, entries 1–10). It was found that electron-rich halides (entries 1, 3, 4) upon coupling with benzo[*b*]furan generally result in better yields than electron-deficient substrates. Only in the case of 4-bromophenol was no conversion observed (entry 2), most likely due to the rather acidic phenolic OH group. For electron-withdrawing substituents a significant trend was observed: The yield decreases with increasing electron-withdrawing nature of the substituent (see trend for entries 5–10), which accounts for the low yield in the case of the 4-NO_2_ substituent, for which the conversion was especially low, even though the 2-arylated product was formed exclusively (entry 10). In this case the homocoupled product of the halide was observed in a significant amount. Next, we investigated the influence of the position of the aryl functional group on the halide (entries 11–14). A methyl group at the *ortho* position was still tolerated, even though the yield dropped to 40 % (entry 12). In contrast, a methoxy group at the *ortho* position inhibited conversion completely (entry 11), presumably due to chelating effects. Subsequent to the oxidative addition into the C–Br bond, a relatively stable palladium complex may be formed due to the adjacent oxygen of the methoxy group; this results in coordination to the metal center through a lone pair, consequently interfering with any further reaction at the metal center.

In addition, we used three different isomers of chlorobromobenzene in the arylation protocol: Variation of reactivity was observed with substitution at the *para* position giving the highest yield (57 %) and *ortho* substitution the lowest (43 %); however, the effect was not very pronounced (entries 5, 13, 14). Notably, we never detected any product originating from a reaction at the chloride position rather than the bromide position. This absolute selectivity enables further transformations at the chloride position in future work.

Next, we investigated whether the same reaction conditions can be translated to other benzo-fused heterocycles as well (Table 3).

**Table 3:** Scope of the direct arylation of other benzo-fused heterocycles[a]

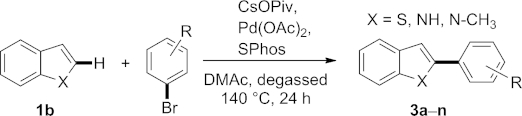				

Entry	X	R	Product	Yield[c] [%]
1	S	H	**3a**	43[c]
2	S	4-OH	**3b**	0
3	S	4-CH_3_	**3c**	34[c]
4	S	4-OCH_3_	**3d**	53
5	S	4-Cl	**3e**	58[c]
6	S	4-F	**3f**	35[c]
7	S	2-OCH_3_	**3g**	0
8	S	2-CH_3_	**3h**	68[c]
9	S	2-Cl	**3i**	60[c]
10	S	4-COOEt	**3j**	45
11	S	4-CN	**3k**	25
12	S	4-NO_2_	**3l**	26
13	NH	H	**3m**	0
14	N-CH_3_	H	**3n**	64[c]

[a] Reaction conditions: Benzo-fused heterocycle substrate (1 equiv.), aryl bromide (1.5 equiv.), CsOPiv (1.5 equiv.), Pd(OAc)_2_ (4 mol-%), SPhos (8 mol-%), 140 °C, 24 h, 0.5 m of substrate in solvent (degassed DMAc).

[b] Isolated yields.

[c] Bis-arylated byproduct observed by GC.

Benzo[*b*]thiophene was subjected to the established reaction conditions as first benchmarking system.[21] In this case bis-arylated benzo[*b*]thiophenes were also observed in several cases but in low quantities (<10 %). In the case of benzo[*b*]thiophene, the byproduct did not impede isolation of the 2-arylated product as observed for benzo[*b*]furan.[13a] The more pronounced difference in the polarity of the byproduct and desired product allowed a more facile purification as compared with the benzo[*b*]furan series. The results of the reactions are quite similar to the data obtained for benzo[*b*]furan. Again, neither a free OH nor an *o*-methoxy group was tolerated (Table 3, entries 2 and 7) and electron-withdrawing substituents had a negative effect on the reaction (entries 6, 10–12). It is noteworthy that *o*-bromotoluene gave a better yield than the corresponding *para*-substituted isomer (entries 8 and 9 vs. 3 and 5).

Finally, we tested our conditions on indole, as well, even though direct arylation is well established on this heterocyclic system.[9] In this context, it was our aim to establish the scope and limitations of the developed protocol as a general method for direct arylation of benzo fused systems. Indole itself did not react, corroborating our above indicated hypothesis of a detrimental effect by acidic protons (entry 13). Blocking this position by employing 1-methylindole, the protocol again turned out as very robust to give a good isolated yield of the arylated product (64 %, entry 14), again accompanied by minor amounts of bis-arylated side-product.

The formation of bis-arylated products as minor contaminant in a number of examples prompted us to exploit the protocol for the synthesis of 2,3-bis-arylated benzo-fused heterocycles as well, starting from C-2 arylated derivatives (Table 4).

**Table 4:** Scope of the direct arylation on C-3 of 2-arylbenzo[*b*]furan[a]

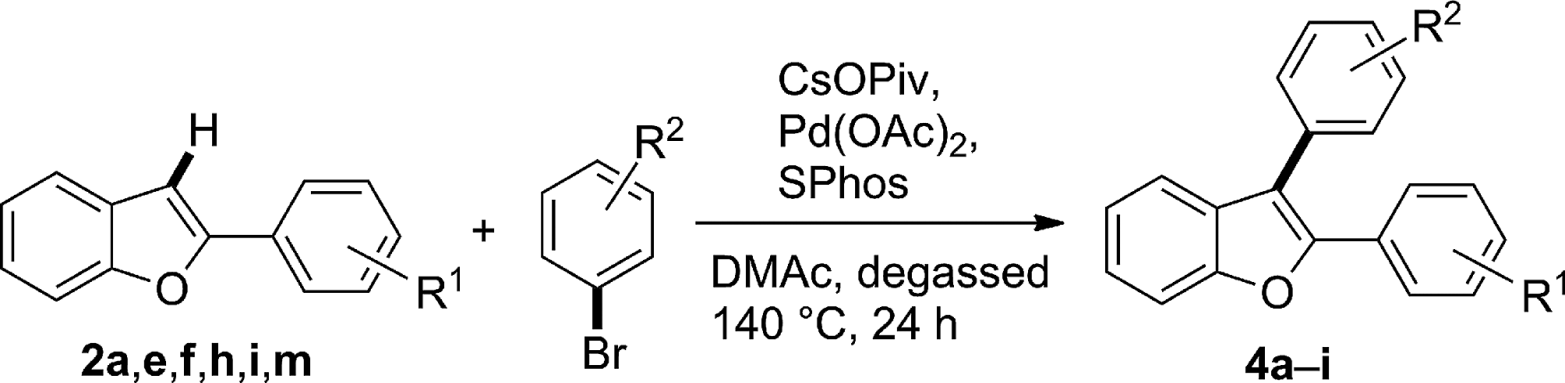				

Entry	R^1^	R^2^	Product	Yield [%]
1	H	H	**4a**	47
2	4-Cl	4-Cl	**4b**	58
3	4-F	4-F	**4c**	44
4	2-Me	2-Me	**4d**	40
5	2-Cl	2-Cl	**4e**	53
6	4-CN	4-CN	**4f**	17
7	H	4-OMe	**4g**	0[b]
8	H	4-Cl	**4h**	50
9	H	4-NO_2_	**4i**	20

[a] Reaction condition: 2-arylbenzo[*b*]furan substrate (1 equiv.), aryl bromide (1.5 equiv.), CsOPiv (1.5 equiv.), Pd(OAc)_2_ (4 mol-%), SPhos (8 mol-%), 140 °C in 24 h, 0.5*M* of substrate in solvent (degassed DMAc), yields were isolated yields.

[b] Observed trace of product on GC.

Initially, we introduced the same aryl residue in the second step at C-3 which was already attached to C-2 (Table 4, entries 1–6). Again, yields were in the same range (but slightly lower) confirming our previously observed trend regarding the electronic nature of substituents on the aryl bromides. The lower yields for C-3 arylation can be rationalized by steric hindrance imposed by the aryl substituent in position 2 (compare entries 1–6/Table 4 to entries 1, 5–9, 11/Table 3).

As a further logical extension of the method, we then introduced different aryls in position 3 than in position 2 (Table 4, entries 7–9). Arylation with 4-chloro-bromobenzene proceeded with similar efficiency compared to the reaction in position 2 (entry 8). 4-Bromoanisole on the other hand did not react at C-3 at all (entry 7). Coincidentally, no bis-arylated product was observed in case of C-2 arylation with 4-bromoanisole (Entry 4 in Table 2). The reason for the failure is not clear at the moment and will be subject of further investigations. Introduction of 4-nitrophenyl in position 3 resulted in a low yield of 20 %, which was expected due to previous results obtained with that particular coupling partner.

## Conclusions

In summary, we developed a generally applicable method to directly arylate benzo-fused heterocycles such as benzo[*b*]furan, benzo[*b*]thiophene, and *N*-methylindole. Even though yields are usually moderate, this protocol is very versatile and generally applicable producing highest yields for direct arylation of benzo[*b*]furan compared to available literature procedures.[11,13] Both electron-rich and electron-poor aryl bromides can be used as aryl source, however, strong electron-withdrawing substitutents have a limiting effect on the efficiency of the protocol.

As already indicated, the method can also be applied to benzo[*b*]thiophene and *N*-methylindole with similar results. The C2-arylated products were susceptible to undergo additional arylation at C-3 employing the same protocol, showing similar trends in reactivity as for C2-arylation. Investigations to translate this protocol to other heterocycles and application of other aryl sources (aryl iodides or chlorides) are currently under investigation in our laboratory.

## Experimental Section

**General Methods:** Chemicals were purchased from commercial suppliers and used without further purification unless otherwise noted. Reactions were followed via TLC (0.25 mm silica gel 60-F plates). Visualization was accomplished with UV light. Flash chromatography was carried out using silica gel 320–400 mesh by MPLC. All solvents for MPLC were distilled prior to use. Dimethylacetamide (DMAc) was distilled and subsequently degassed by bubbling argon through the solvent in an ultrasonic bath for 2 h. Palladium catalysts and ligands were stored under argon in a desiccator and weighed in under air. ^1^H and ^13^C NMR were recorded from CDCl_3_ solutions on a Bruker AC 200. Chemical shifts (*δ*) are reported in parts per million (ppm) with trimethylsilane (TMS) as the internal standard. The abbreviations used to report the data are s (singlet), d (doublet), t (triplet), q (quartet), m (multiplet), and br. (broad). GC–MS spectra were recorded either on a Thermo Finnigan Focus GC/DSQ II using a standard capillary column BGB 5 (30 m × 0.25 mm ID) or a Thermo Trace 1300/ISQ LT using a standard capillary column BGB 5 (30 m × 0.25 mm ID). HR-MS for literature unknown compounds were analyzed by LC-IT-TOF-MS in only positive ion detection mode with the recording of MS and MS/MS spectra. Melting points were determined using a Stanford Research Systems MPA100 OptiMelt Automatic Melting Point System. Data is given in 0.5 °C intervals.

**General Procedure for Direct Arylation of Benzo-fused Heterocycles:** A 7 mL vial equipped with a screw cap with septum and a magnetic stirring bar was charged with benzo[*b*]furan or either benzothiophene, indole or 1-methyl indole (1.0 mmol, 1.0 equiv.), aryl bromide (1.5 mmol, 1.5 equiv.), caesium pivalate (350 mg, 1.5 mmol, 1.5 equiv.), Pd(OAc)_2_ (9 mg, 0.04 mmol, 0.04 equiv.) and Sphos (16.4 mg, 0.08 mmol, 0.08 equiv.). The vial was evacuated and flushed with argon 3 times. Then 2 mL of degassed DMAc were added via syringe. The mixture was heated to 140 °C for 24 h. The reaction mixture was cooled to room temperature and then diluted with 15 mL diethyl ether or ethyl acetate (depending on the polarity of the product) and filtered through a pad of celite. The organic phase was washed 3 times with saturated ammonium chloride solution, once with brine and dried with sodium sulfate. The solvent was removed under reduced pressure. Purification was performed on silica gel eluting with PE (petroleum ether) or PE/EtOAc (ethyl acetate) mixtures (depending on the polarity of the product), if mixed fractions of C2 & C3 arylated compounds were obtained, these fractions were recrystallized.

**Representative Examples**

**2-Phenylbenzo[*b*]furan:** Synthesized according to the general procedure starting from benzo[*b*]furan (110 μL, 118 mg, 1.0 mmol), bromobenzene(105 μL, 157 mg, 1.5 mmol), caesium pivalate (350 mg, 1.5 mmol), Pd(OAc)_2_ (9 mg, 0.04 mmol) and Sphos (16.4 mg, 0.08 mmol). Purification was employed in two steps: First column chromatography was carried out (silica gel, pure PE as eluent) which afforded 155 mg of a product mixture containing 2-phenylbenzo[*b*]furan and 2,3-bisphenylbenzo[*b*]furan (respective mol ratio 10:1). This product mixture was dissolved in 1 mL PE at 50 °C. The solution was cooled to ca. –5–0 °C and kept at that temperature overnight. The precipitated crystals were separated by filtration, washed 3 times with cold PE (–5 °C) and dried in vacuo to obtain 116 mg (60 %) of 2-phenylbenzo[*b*]furan as a colorless solid. *R*_f_ = 0.64 (PE 100 %). Mp. 120–121 °C. ^1^H NMR (200 MHz, CDCl_3_): *δ* = 7.95–7.80 (m, 2 H), 7.69–7.14 (m, 8 H), 7.03 (s, 1 H) ppm. ^13^C NMR (50 MHz, CDCl_3_): *δ* = 155.9, 154.9, 130.5, 129.2, 128.8 (2 C), 128.6, 124.9 (2 C), 124.3, 122.9, 120.9, 111.2, 101.3 ppm. MS analyst, *m*/*z* (Int.): 194(100) [M^+^], 165(50), 97(9), 82(12).

**4,4′-(Benzo[*b*]furan-2,3-diyl)dibenzonitrile (4f):** Synthesized according to the general procedure starting from 4-(benzo[*b*]furan-2-yl)benzonitrile (**2m**) (110 mg, 0.5 mmol), 4-bromobenzonitrile (136 mg, 0.75 mmol), caesium pivalate (175 mg, 0.75 mmol), Pd(OAc)_2_ (4.5 mg, 0.02 mmol) and Sphos (8.2 mg, 0.04 mmol). Purification by MPLC (silica gel with gradient elution PE → PE/EtOAc, 95:5) afforded 27 mg (34 %) **4f** as pale yellow solid. Mp. 178–183 °C. ^1^H NMR (200 MHz, CDCl_3_): *δ* = 7.83–7.79 (m, 2 H), 7.73–7.69 (m, 2 H), 7.64–7.59 (m, 5 H), 7.49–7.39 (m, 2 H), 7.33 (dd, *J* = 7.4, 1.1 Hz, 1 H) ppm. ^13^C NMR (50 MHz, CDCl_3_): *δ* = 154.3, 148.9, 137.2, 134.1, 133.1 (2 C), 132.5 (2 C), 130.4 (2 C), 128.9, 127.3 (2 C), 126.4, 123.9, 120.0, 118.6, 118.5, 118.4, 112.1, 112.1, 111.6 ppm. MS Analyst, *m*/*z* (Int.): 321(21), 320(100) [M^+^], 291(13), 289(11), 264(12), 206(18), 190(10), 118(12).

**Supporting Information** (see footnote on the first page of this article): Complete tables for reaction optimization, experimental details, NMR spectroscopic data and scanned spectra of all prepared compounds.

## Supporting Information

As a service to our authors and readers, this journal provides supporting information supplied by the authors. Such materials are peer reviewed and may be re-organized for online delivery, but are not copy-edited or typeset. Technical support issues arising from supporting information (other than missing files) should be addressed to the authors.

miscellaneous_information
