# Supplementary material for: Direct Arylation of Benzo[b]furan and Other Benzo-Fused Heterocycles
Source: European J Org Chem. 2014 Nov 17;2014(36):8119–25. doi: 10.1002/ejoc.201403125 (PMC4502765; doi:10.1002/ejoc.201403125)
Supplement: Supplementary file 1 — miscellaneous_information [file ejoc2014-8119-sd1.pdf]

Eur. J. Org. Chem. **2014** · © WILEY-VCH Verlag GmbH & Co. KGaA, 69451 Weinheim, 2014 · ISSN 1099–0690

SUPPORTING INFORMATION

DOI: 10.1002/ejoc.201403125
Title: Direct Arylation of Benzo[*b*]furan and Other Benzo-Fused Heterocycles
Author(s): Toan Dao-Huy, Maximilian Haider, Fabian Glatz, Michael Schnürch,* Marko D. Mihovilovic

Contents

Optimization of the C-H activation 2

Evaluation of C-H activation of benzo-fused heterocycles 5

Characterization and NMR spectra of C-H activation products 6

References 37

Optimization of the C-H activation

A 7mL vial equipped with a screw cap with septum and a magnetic stirring bar was charged with benzo[*b*]furan (118 mg, 1.0 mmol), 4-bromoanisole (280 mg, 190 µL, 1.5 mmol), base (1.5 mmol), Pd catalyst, ligand (if necessary) and dodecane (85 mg, 113 µL, 0.5 mmol) as internal standard. The vial was closed, evacuated and flushed with argon 3 times. Then 2 mL of degassed DMAc were added by syringe. The mixture was heated to 140ºC. The reaction was monitored by TLC and GC. The conversion and GC-yield was calculated on relative peak area respect to internal standard.

The optimistic condition was applied to all evaluation reactions as general condition.

Table S1. Screening of $Pd(OAc)_2$ amount for direct arylation of benzo[*b*]furan.[a]

Entry	$Pd(OAc)_2$	Conversion (%)	Yield (%)	C2:C3:Bis
1	1 mol%	18	14	100:16:00
2	2 mol%	22	16	100:17:00
**3**	**4 mol%**	**27**	**22**	**100:17:00**
4	6 mol%	32	23[b]	100:15:00
5	8 mol%	36	22[b]	100:16:00
6	10 mol%	41	20[b]	100:17:00

[a] Reaction condition: benzo[*b*]furan (1 eq.), 4-bromoanisole (1.5 eq.), KOAc (1.5 eq.), $Pd(OAc)_2$ as catalyst without ligand, 140 °C in 24 hours, 0.5M of substrate in solvent (degassed DMAc). The yield was determined by GC. [b] Observed homocoupling by-products of 4-bromoanisole.

Table S2. Ligand Screening for direct arylation of benzo[*b*]furan.[a]

Entry	Base	Pd cat.	Ligand	Yield	C2:C3:Bis
1	KOAc	$Pd(OAc)_2$	*-No ligand-*	22	100:17:00
2	KOAc	$Pd(OAc)_2$	Tri-2-furylphosphine	15	100:22:00
3	KOAc	$Pd(OAc)_2$	Tris(p-chlorophenyl)phosphine	22	100:26:00
4	KOAc	$Pd(OAc)_2$	JohnPhos	10	100:19:00
5	KOAc	$Pd(OAc)_2$	XPhos	7	100:16:00
**6**	**KOAc**	**$Pd(OAc)_2$**	**SPhos**	**45**	**100:14:00**
7	KOAc	$Pd(OAc)_2$	CPhos	40	100:21:00

[a] Reaction condition: benzo[*b*]furan (1 eq.), 4-bromoanisole (1.5 eq.), KOAc (1.5 eq.), $Pd(OAc)_2$ (4 mol%), ligand (8 mol%), 140 °C in 24 hours, 0.5M of substrate in solvent (degassed DMAc). The yield was determined by GC.

Table S3. Screening of Pd species for direct arylation of benzo[*b*]furan.[a]

Entry	Base	Pd cat.	Ligand	Yield	C2:C3:Bis
1	KOAc	$Pd(OAc)_2$	CPhos	40	100:21:00
**2**	**KOAc**	**$Pd(OAc)_2$**	**SPhos**	**45**	**100:14:00**
3	KOAc	$Pd(PPh_3)_2Cl_2$	CPhos	38	100:20:00
4	KOAc	$Pd(PPh_3)_2Cl_2$	SPhos	40	100:13:00
5	KOAc	$Pd(PPh_3)_4$	CPhos	33	100:31:00
6	KOAc	$Pd(PPh_3)_4$	SPhos	44	100:29:00

[a] Reaction condition: benzo[*b*]furan (1 eq.), 4-bromoanisole (1.5 eq.), KOAc (1.5 eq.), Pd cat. (4 mol%), ligand (8 mol%), 140°C in 24hours, 0.5M of substrate in solvent (degassed DMAc). The yield was determined by GC.

Table S4. Screening of bases for direct arylation of benzo[*b*]furan.[a]

Entry	Base	Pd cat.	Ligand	Yield	C2:C3:Bis
1	KOAc	$Pd(OAc)_2$	SPhos	45	100:14:00
2	CsOAc	$Pd(OAc)_2$	SPhos	19	100:24:00
3	^{t}BuOK	$Pd(OAc)_2$	SPhos	7	100:09:00
4	K_2CO_3	$Pd(OAc)_2$	SPhos	13	100:12:00
5	Cs_2CO_3	$Pd(OAc)_2$	SPhos	25	100:09:00
	KOPiv	$Pd(OAc)_2$	*-No ligand-*	44	100:10:00
	KOPiv	$Pd(OAc)_2$	SPhos	65	100:08:00
	CsOPiv	$Pd(OAc)_2$	*-No ligand-*	54	100:11:00
**6**	**CsOPiv**	**$Pd(OAc)_2$**	**SPhos**	**72**	**100:05:00**

[a] Reaction condition: benzo[*b*]furan (1 eq.), 4-bromoanisole (1.5 eq.), base (1.5 eq.), $Pd(OAc)_2$ (4 mol%), ligand (8 mol%), 140°C in 24hours, 0.5M of substrate in solvent (degassed DMAc). The yield was determined by GC. [b] Using 2 mol% instead of 4 mol% of Pd cat.

Table S5. Ligand Screening revisited after optimization of the base.[a]

Entry	Base	Pd cat.	Ligand	Yield	C2:C3:Bis
1	CsOPiv	$Pd(OAc)_2$	2,2'-bipyridine	34	100:06:00

2	CsOPiv	$Pd(OAc)_2$	2,2'-bipyridine	42	100:05:00
3	CsOPiv	$Pd(OAc)_2$	1,10-Phenanthroline	6	100:15:00
4	CsOPiv	$Pd(OAc)_2$	4,4'-di-*tert*-butyl-2,2'-bipyridine	16	100:13:00

[a] Reaction condition: benzo[*b*]furan (1 eq.), 4-bromoanisole (1.5 eq.), base (1.5 eq.), $Pd(OAc)_2$ (4 mol%), ligand (8 mol%), 140 °C in 24 hours, 0.5M of substrate in solvent (degassed DMAc). The yield was determined by GC. [b] Using 0.16 eq. of 2,2'-bipyridine ligand.

Evaluation of C-H activation of benzo-fused heterocycles

General procedure: A 7mL vial equipped with a screw cap with septum and a magnetic stirring bar was charged with benzo[*b*]furan or either benzothiophene, indole or 1-methyl indole (1.0 mmol, 1.0 eq.), arylbromide (1.5 mmol, 1.5 eq.), cesium pivalate (350mg, 1.5 mmol, 1.5 eq.), $Pd(OAc)_2$ (9mg, 0.04mmol, 0.04 eq.) and Sphos (16.4 mg, 0.08 mmol, 0.08 eq.). The vial was evacuated and flushed with argon 3 times. Then 2 mL of degassed DMAc were added via syringe. The mixture was heated to 140 °C for 24 hours. The reaction mixture was cooled to room temperature and then diluted with 15mL diethylether or ethylacetate (depending on the polarity of the product) and filttrated through a pad of celite. The organic phase was washed 3 times with saturated ammonium chloride solution, once with brine and dried over sodium sulfate. The solvent was removed under reduced pressure. Purification was performed on silica gel eluting with PE (petroleum ether) or PE/EtOAc (ethyl acetate) mixtures (depending on the polarity of the product), if mixed fractions of C2 & C3 arylated compounds were obtained, these fractions were recrystallized. For that purpose the product mixture was dissolved in the least amount of boiling PE. The solution was cooled to -5 – 0 ºC and left to crystallize overnight. The crystals were filtrated and washed 3 times with cold PE (-5 ºC). The solid material was dried in vacuum.

The same procedure was used for C-3 arylation of 2-arylbezon[*b*]furans.

Characterization and NMR spectra of C-H activation products

2-Phenylbenzofuran (2a) [1]: Synthesized according to the general procedure as colorless solid (58 mg product, 60% yield). mp 120-121ºC (Lit. 121−122ºC); **^{1}H NMR (200 MHz, $CDCl_3$)** δ (ppm) = 7.95 – 7.80 (m, 2H), 7.69 – 7.14 (m, 8H), 7.03 (s, 1H). **^{13}C NMR (50 MHz, $CDCl_3$)** δ (ppm) = 155.9, 154.9, 130.5, 129.2, 128.8(2C), 128.6, 124.9(2C), 124.3, 122.9, 120.9, 111.2, 101.3. **MS analyst, m/z (Int.)**: 195(16), 194(100), 165(50), 97(9), 82(12).

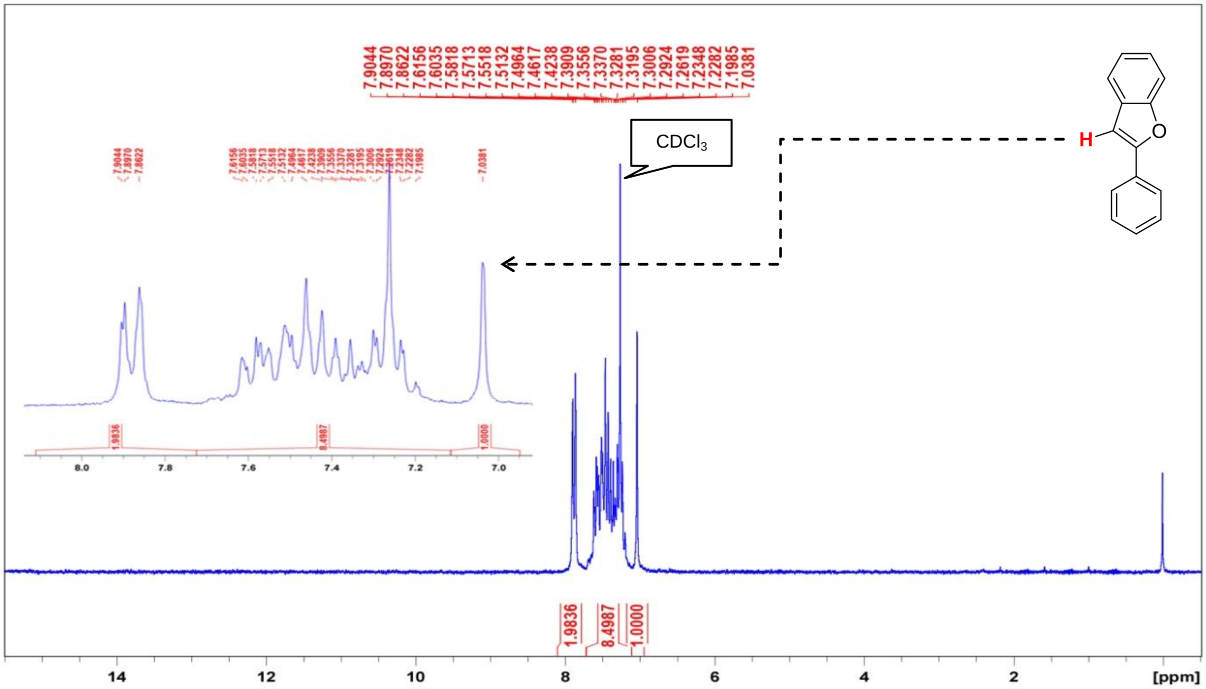

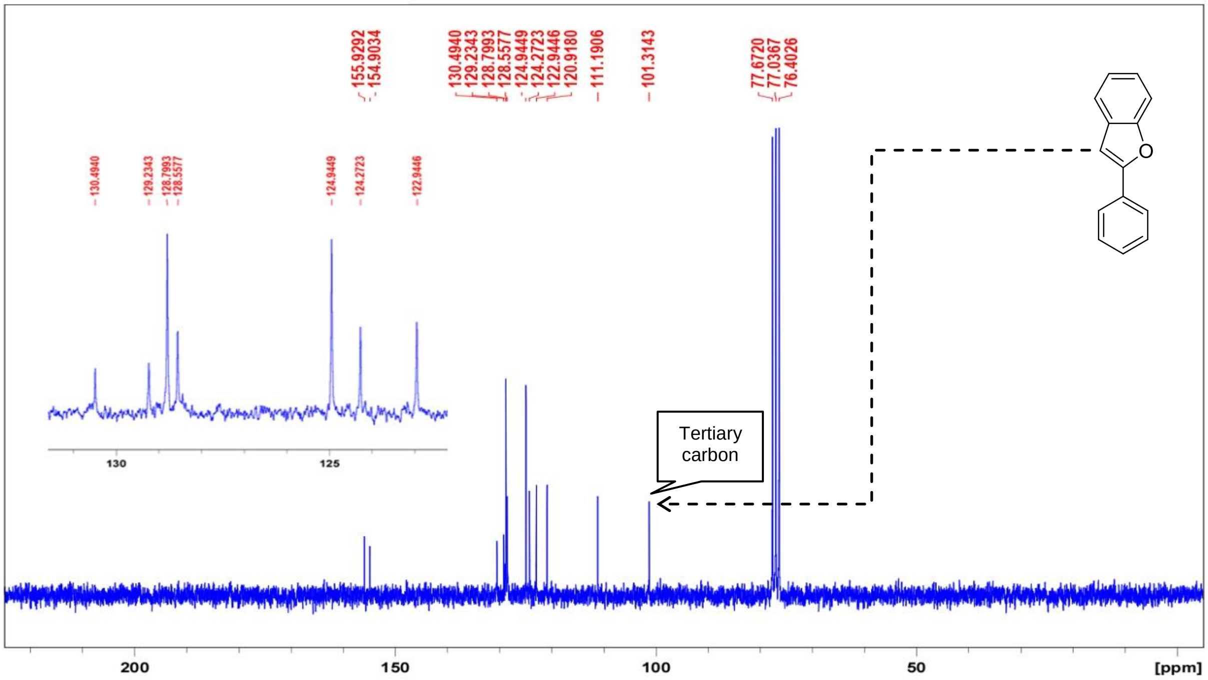

2-(p-Tolyl)benzofuran (2c) [2]: Synthesized according to the general procedure as colorless solid (56 mg product, 54% yield). mp 125-127ºC (Lit. 126−128ºC); **^{1}H NMR (200 MHz, $CDCl_3$)** δ (ppm) = 7.77 – 7.73 (m, 2H), 7.57 – 7.48 (m, 2H), 7.30 – 7.16 (m, 4H), 6.94 (d, *J* = 0.8 Hz, 1H), 2,38 (s, 3H). **^{13}C NMR (50 MHz, $CDCl_3$)** δ (ppm) = 156.2, 154.8, 138.6, 129.5(2C), 129.4, 127.8, 124.9(2C), 124.0, 122.9, 120.8, 111.1, 100.6, 21.4. **MS analyst, m/z (Int.)**: 209(17), 208(100), 207(29), 178(15), 165(16), 89(9).

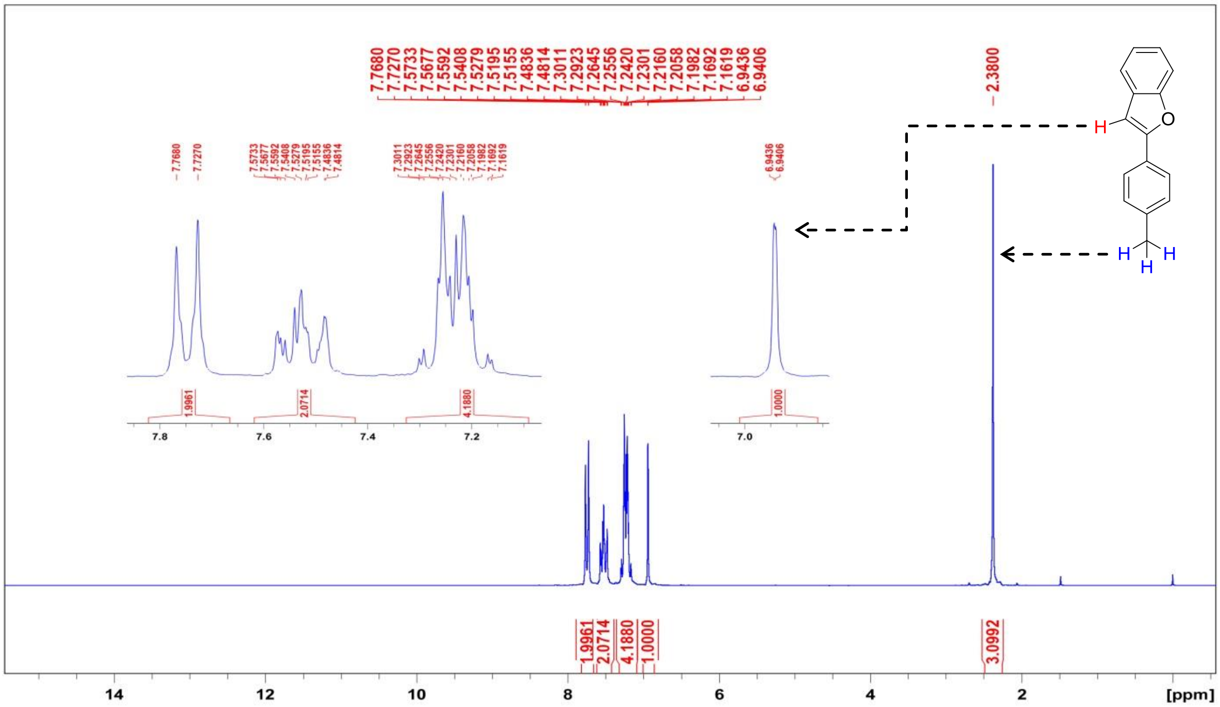

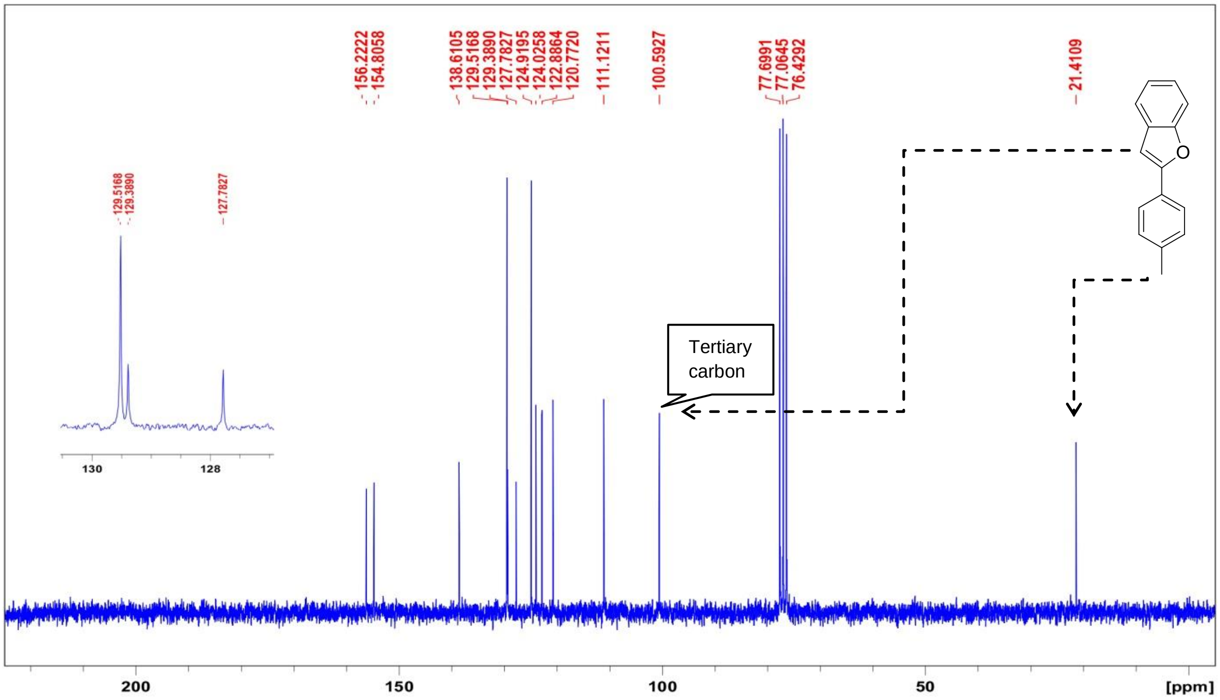

2-(4-Methoxyphenyl)benzofuran (2d) [3]: Synthesized according to the general procedure as colorless solid (78 mg product, 70% yield); mp 140-142ºC (Lit. 148−150ºC); **^{1}H NMR (200 MHz, $CDCl_3$)** δ (ppm) = 7.81 – 7.76 (m, 2H), 7.56 – 7.47 (m, 2H), 7.29 – 7.15 (m, 2H), 6.98 – 6.94 (m, 2H), 6.86 (d, *J* = 0.8 Hz, 1H), 3.83 (s, 3H). **^{13}C NMR (50 MHz, $CDCl_3$)** δ (ppm) = 160.0, 156.1, 154.7, 129.5, 126.4(2C), 123.7, 123.4, 122.8, 120.6, 114.3(2C), 111.0, 99.7, 55.4. **MS analyst, m/z (Int.)**: 225(16), 224(100), 210(12), 209(82), 207(29), 181(52), 152(34), 112(20), 76(12).

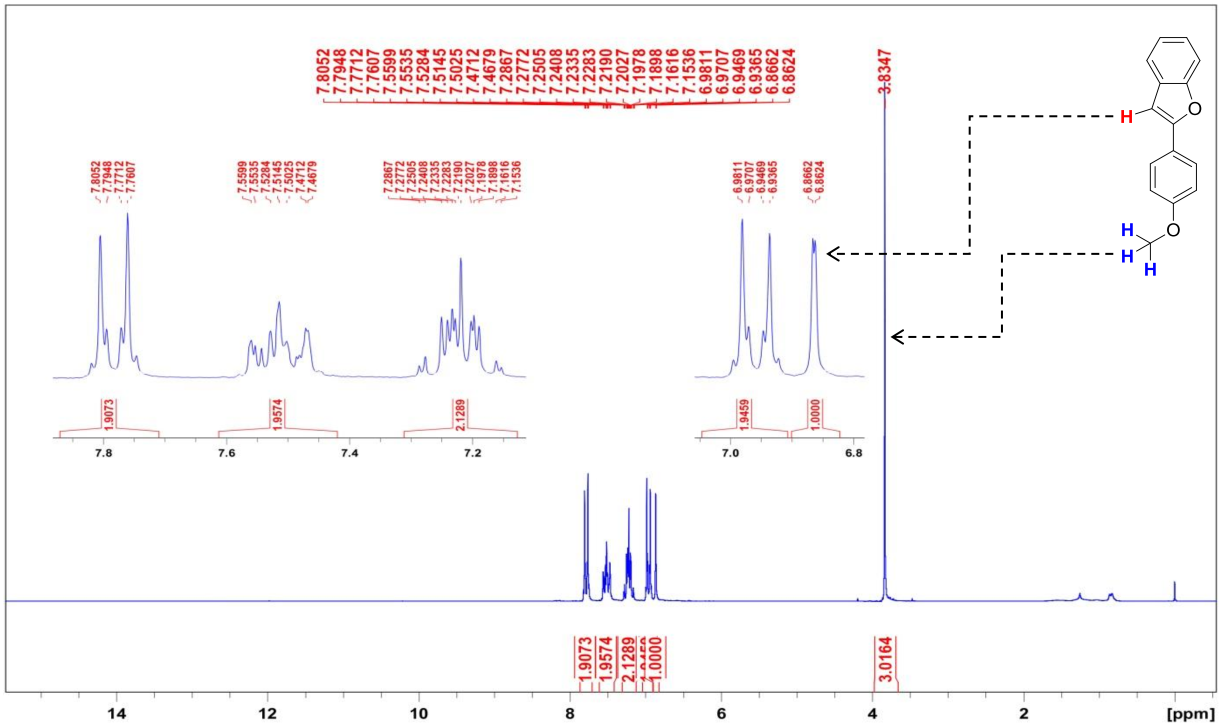

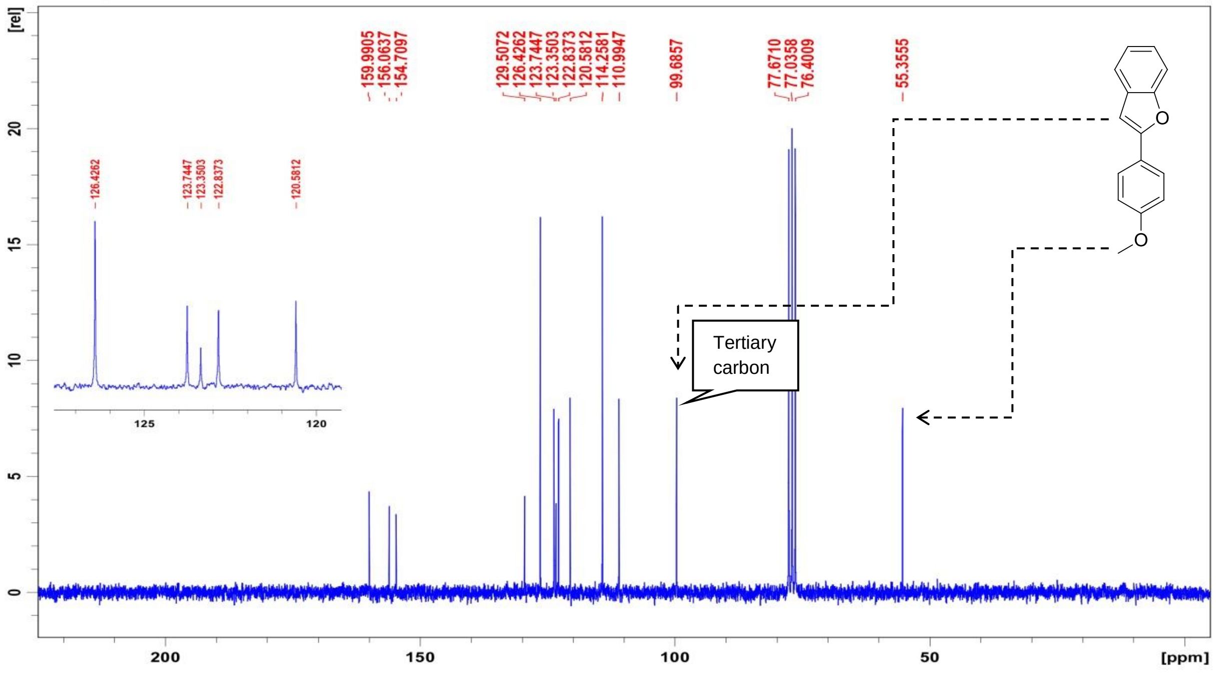

2-(4-Chlorophenyl)benzofuran (2e) [4]: Synthesized according to the general procedure as colorless solid (64 mg product, 57% yield; mp 144-145ºC (Lit. 148−149ºC); **^{1}H NMR (200 MHz, $CDCl_3$)** δ (ppm) = 7.80 – 7.76 (m, 2H), 7.63 – 7.14 (m, 7H), 7.00 (s, 1H). **^{13}C NMR (50 MHz, $CDCl_3$)** δ (ppm) = 154.9, 154.8, 134.3, 129.0(3C), 129.0, 126.1(2C), 124.6, 123.1, 121.0, 111.2, 101.7. **MS analyst, m/z (Int.)**: 230(32), 229(15), 228(100), 165(67), 164(18), 163(20), 115(16), 114(34), 82(30), 81(55), 63(12).

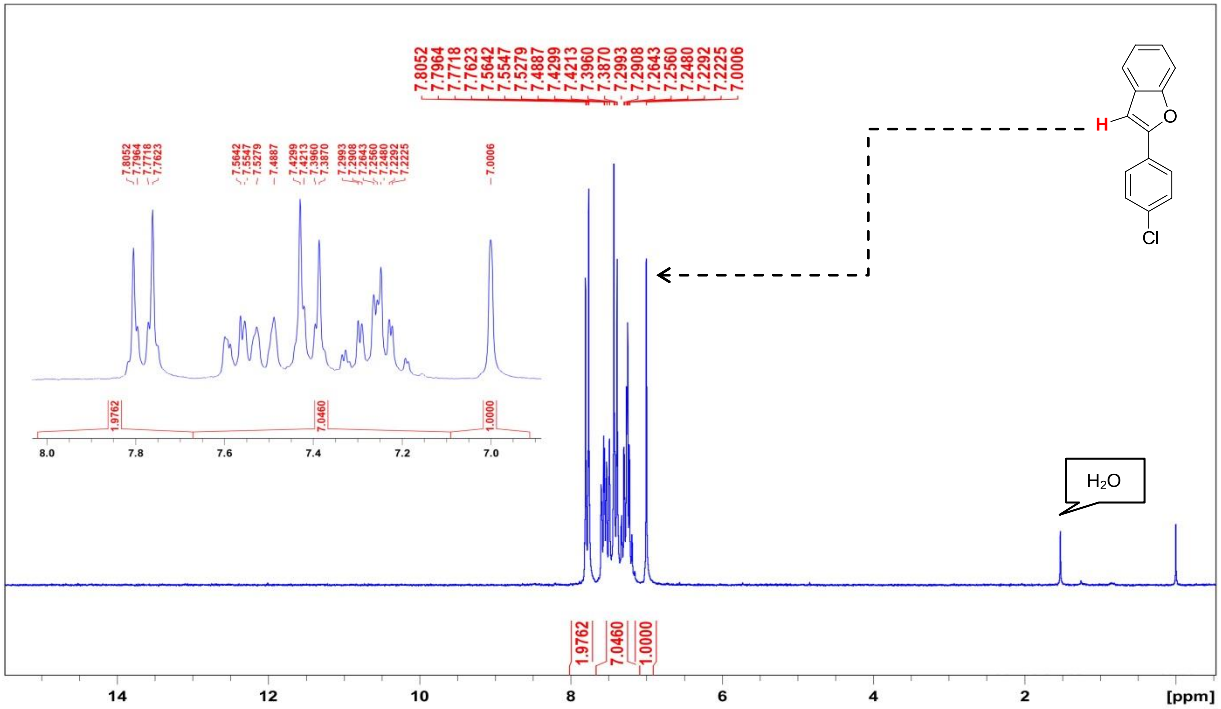

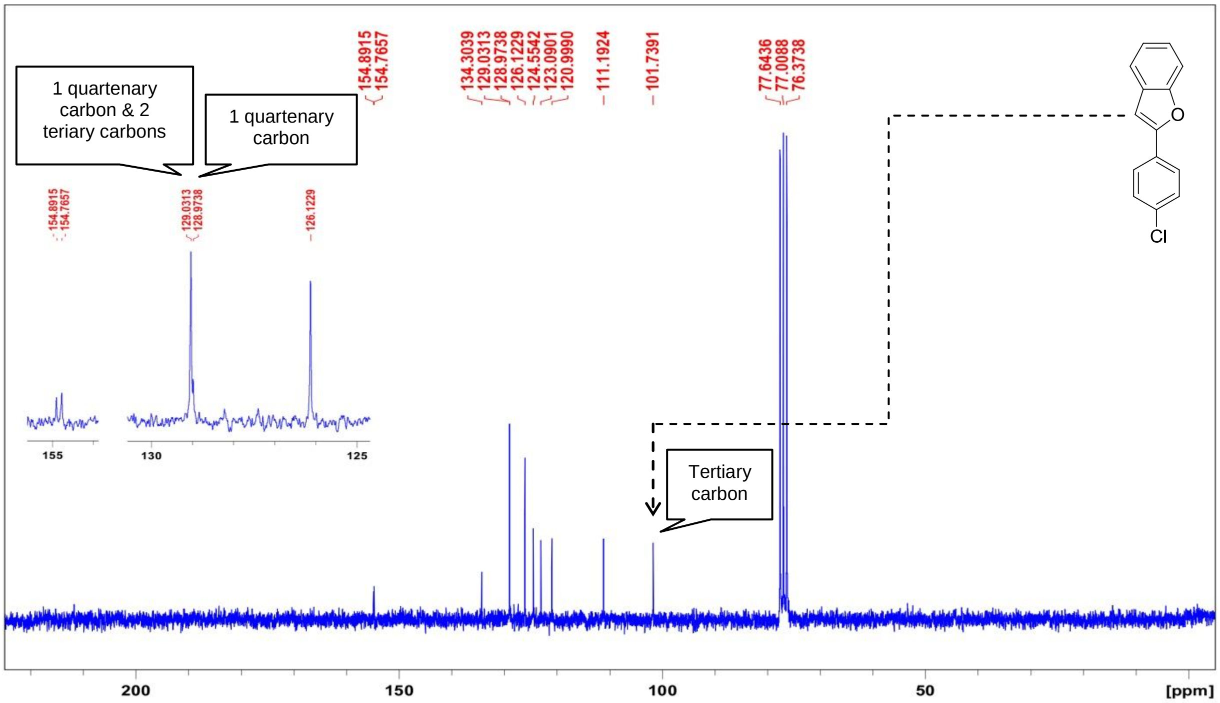

2-(4-Fluorophenyl)benzofuran (2f) [5]: Synthesized according to the general procedure as colorless solid (53 mg product, 50% yield); mp 120-122ºC (Lit. 124ºC); **^{1}H NMR (200 MHz, $CDCl_3$)** δ (ppm) = 7.87 – 7.80 (m, 2H), 7.59 – 7.49 (m, 2H), 7.33 – 7.09 (m, 4H), 6.95 (s, 1H). **^{13}C NMR (50 MHz, $CDCl_3$)** δ (ppm) = 162.8(d, J_{CF} = 247Hz), 155.0, 154.8, 129.2, 126.8, 126.7 (d, J_{CF} = 8Hz, 2C) , 124.3, 123.0, 120.9, 115.9 (d, J_{CF} = 20Hz, 2C), 111.1, 101.0. **MS analyst, m/z (Int.)**: 213(13), 212(100), 183(69), 106(35), 91(26), 82(13).

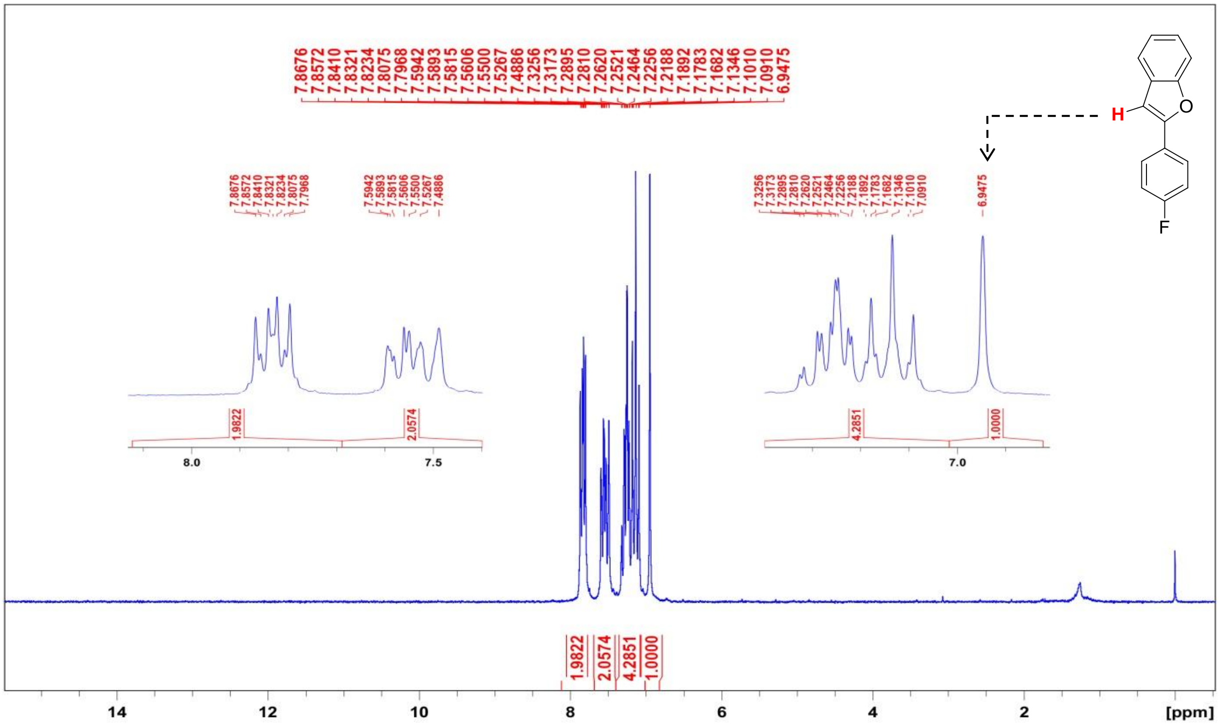

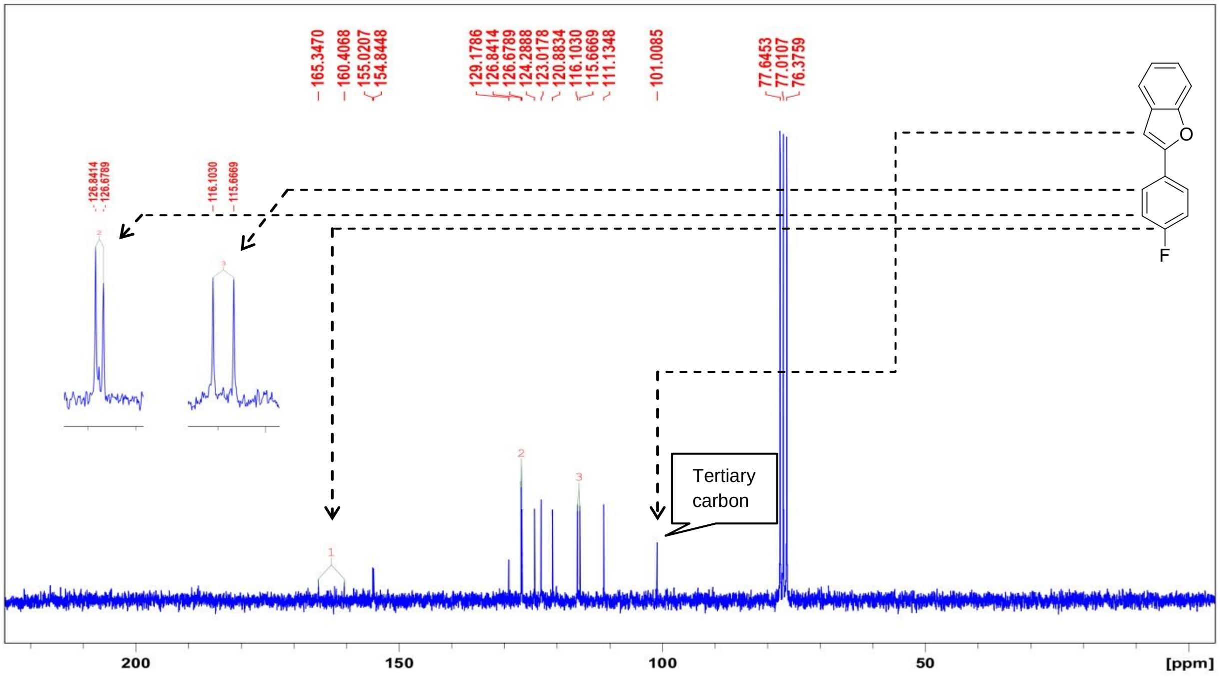

2-(o-Tolyl)benzofuran (2h) [6]: Synthesized according to the general procedure as colorless oil (41 mg product, 40% yield).; **^{1}H NMR (200 MHz, $CDCl_3$)** δ (ppm) = 7.87 – 7.82 (m, 1H), 7.62 – 7.50 (m, 2H), 7.33 – 7.19 (m, 5H), 6.88 (d, *J* = 0.8 Hz, 1H), 2.58 (s, 3H). **^{13}C NMR (50 MHz, $CDCl_3$)** δ (ppm) = 155.7, 154.4, 135.8, 131.3, 130.0, 129.2, 128.5, 128.2, 126.1, 124.3, 122.8, 120.9, 111.1, 105.1, 22.0. **MS analyst, m/z (Int.):** 209(15), 208(100), 207(67), 189(13), 179(20), 178(30), 165(13), 152(14), 115(14), 89(16).

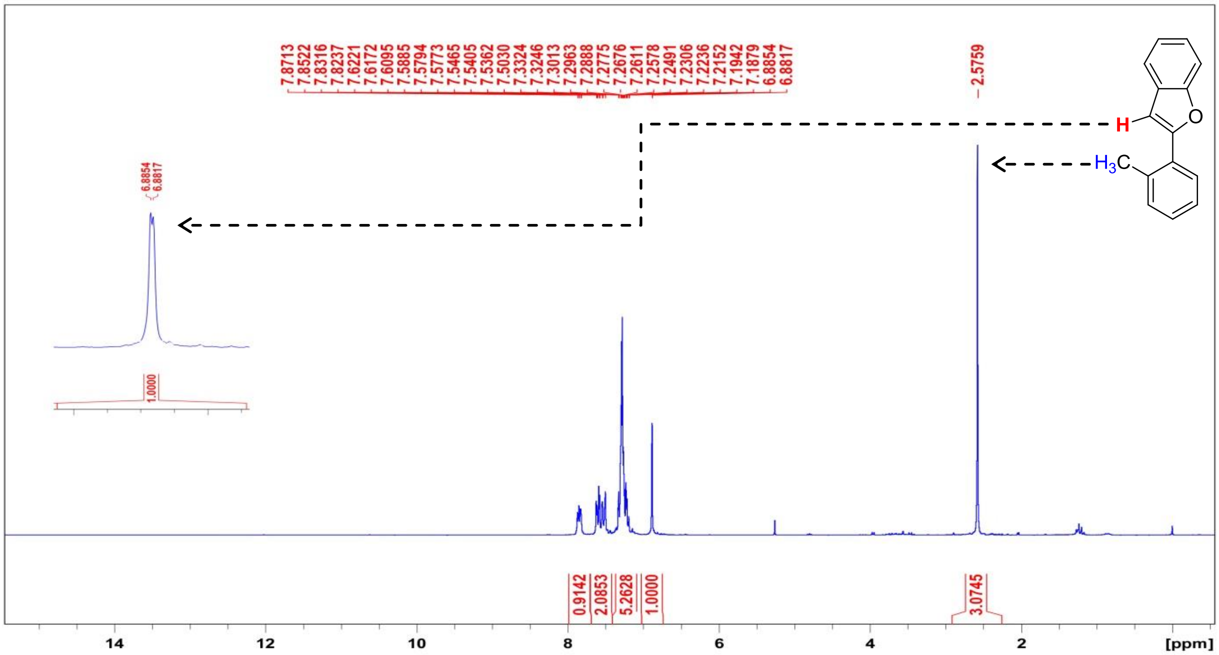

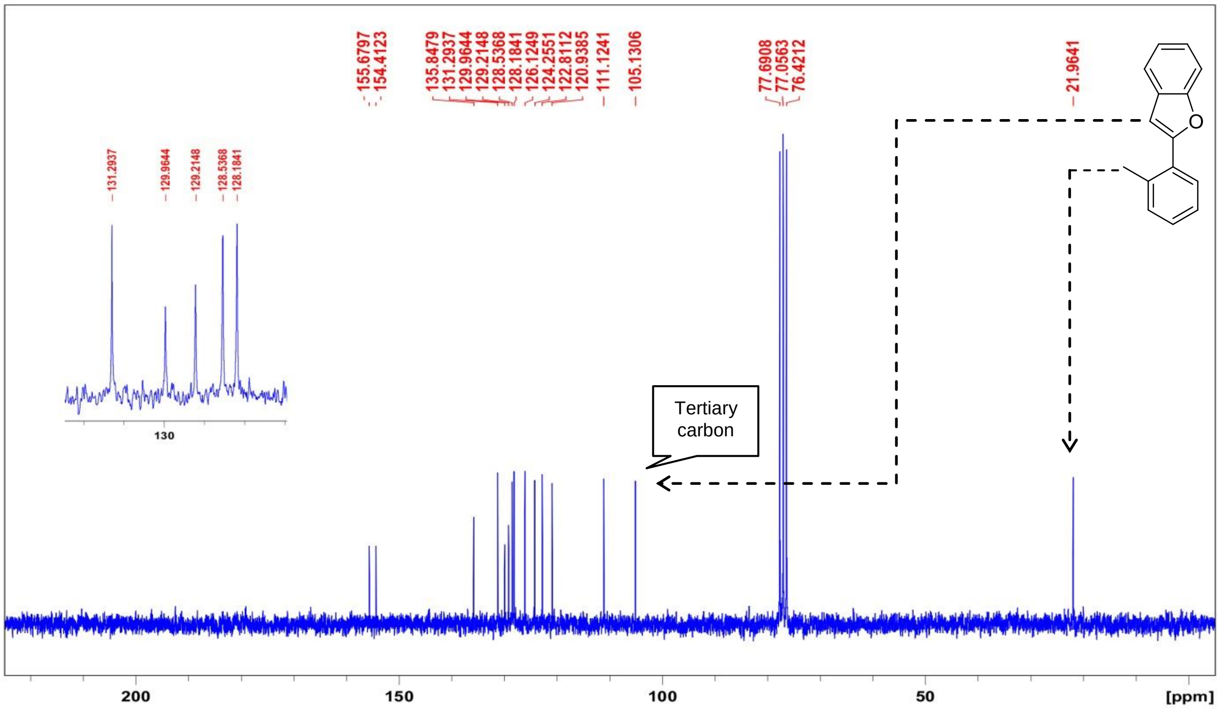

2-(2-Chlorophenyl)benzofuran (2i) [7]: Synthesized according to the general procedure as colorless solid (49 mg product, 43% yield); mp 48ºC (Lit. 46ºC); **^{1}H NMR (200 MHz, $CDCl_3$)** δ (ppm) = 8.03 (dd, *J* = 7.8, 1.6 Hz, 1H), 7.64 – 7.60 (m, 1H), 7.54 – 7.46 (m, 3H), 7.40 – 7.20 (m, 4H). **^{13}C NMR (50 MHz, $CDCl_3$)** δ (ppm) = 154.2, 152.0, 131.3, 130.9, 129.0, 129.0, 128.9(2C), 127.0, 124.9, 123.0, 121.5, 111.1, 107.4. **MS analyst, m/z (Int.)**: 230(34), 229(15), 228(100), 165(64), 164(17), 163(18), 114(12), 82(13).

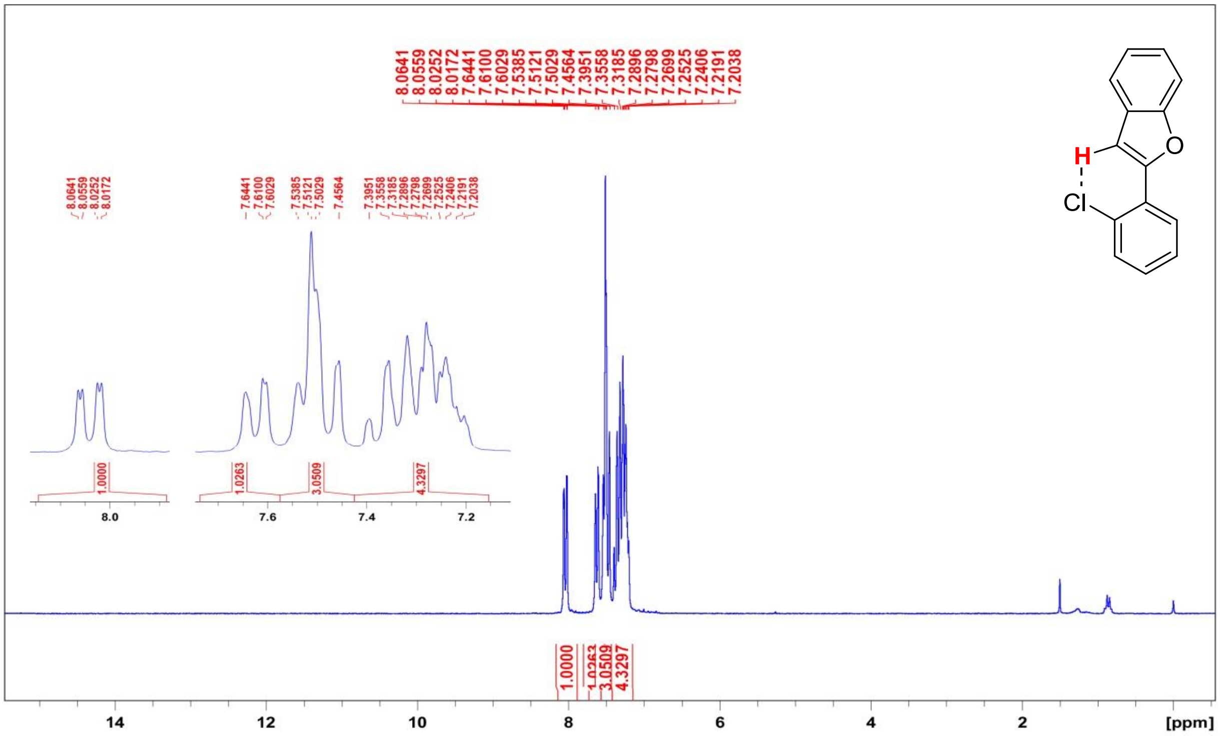

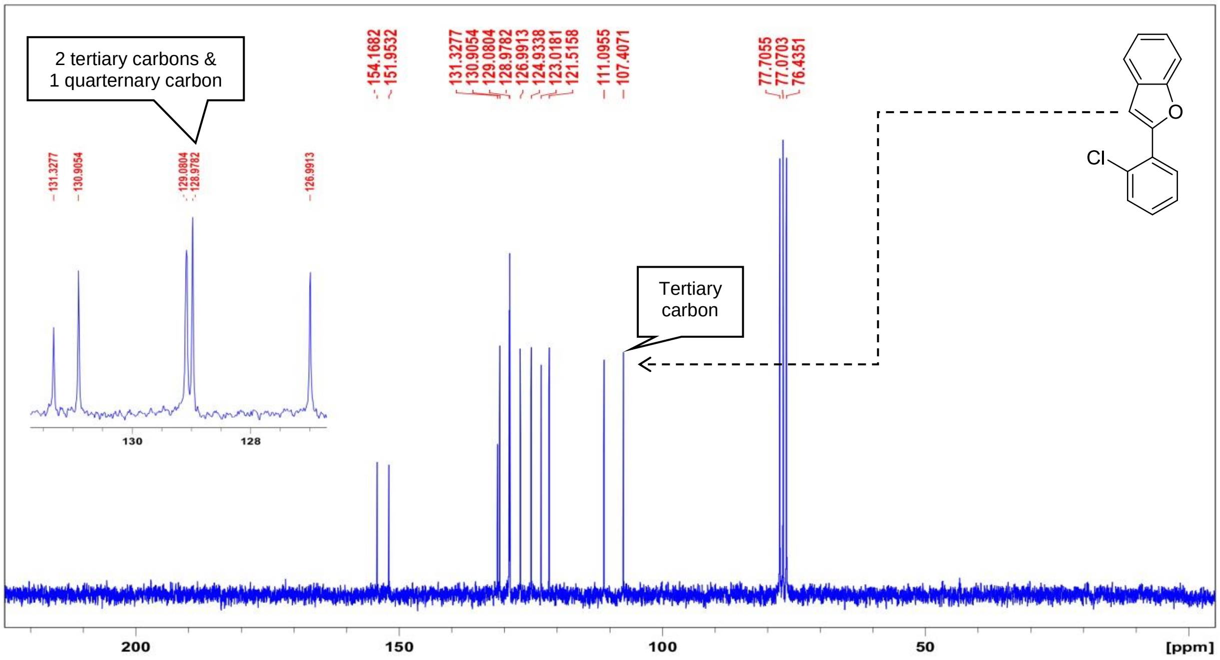

2-(3-Chlorophenyl)benzofuran (2j) [8]: Synthesized according to the general procedure as colorless solid (57 mg product, 50% yield); mp 80-82ºC (Lit 85ºC); **^{1}H NMR (200 MHz, $CDCl_3$)** δ (ppm) = 7.86 – 7.85 (m, 1H), 7.76 – 7.71 (m, 1H), 7.61 – 7.50 (m, 2H), 7.41 – 7.20 (m, 5H), 7.46 (d, *J* = 0.7 Hz, 1H). **^{13}C NMR (50 MHz, $CDCl_3$)** δ (ppm) = 154.9, 154.3, 134.9, 132.2, 130.1, 128.9, 128.4, 124.9, 124.8, 123.1, 122.9, 121.1, 111.3, 102.4. **MS Analyst, m/z (Int.)**: 230(32), 229(15), 228(100), 165(66), 164(18), 163(21), 115(17), 114(35), 83(33), 82(28), 81(20), 63(10).

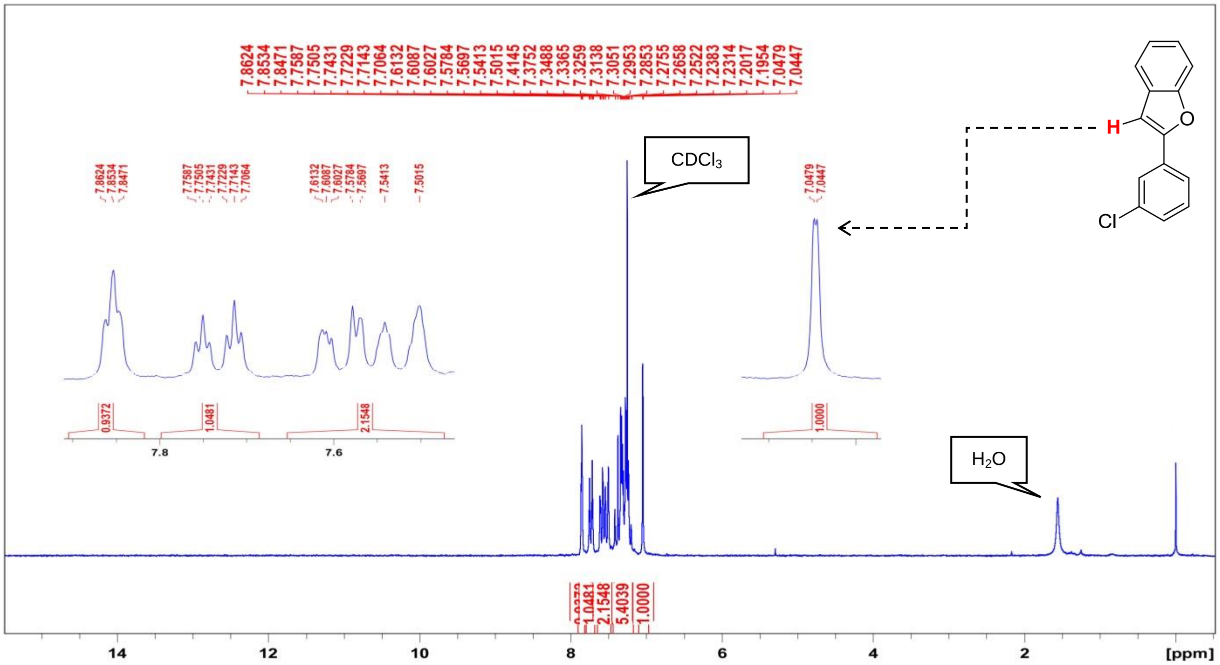

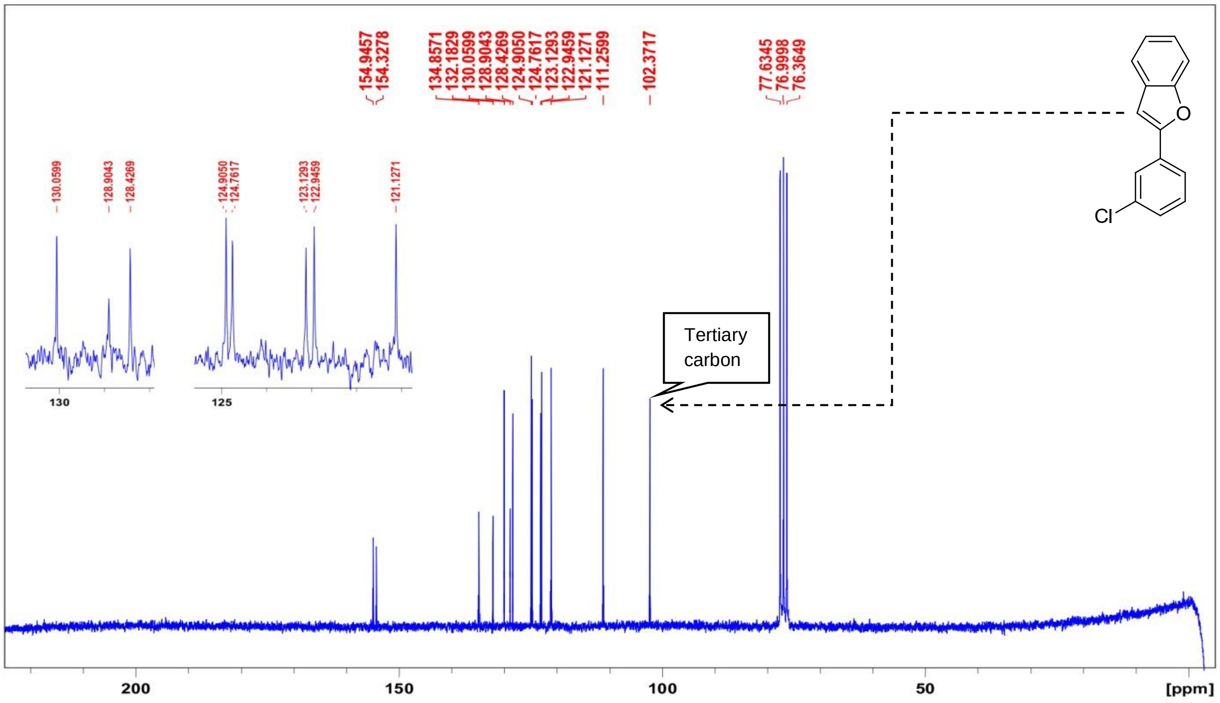

Ethyl 4-(benzofuran-2-yl)benzoate (2k) [9]**:** Synthesized according to the general procedure as light yellow solid (60 mg product, 45% yield); mp 120-121 ºC; **^{1}H NMR (200 MHz, $CDCl_3$)** δ (ppm) = 8.12 – 8.08 (m, 2H), 7.91 – 7.87 (m, 2H), 7.61 – 7.50 (m, 2H), 7.35 – 7.19 (m, 2H), 7.11 (d, *J* = 0.7 Hz, 1H), 4.39 (q, *J* = 7.1 Hz, 2H), 1.40 (t, *J* = 7.1 Hz, 3H). **^{13}C NMR (50 MHz, $CDCl_3$)** δ (ppm) = 166.2, 155.1, 154.7, 134.4, 130.1(2C), 129.8, 128.9, 125.0(2C), 124.5, 123.2, 121.3, 111.3, 103.4, 61.1, 14.4. **MS analyst, m/z (Int.):** 266(100), 238(69), 221(86), 193(24), 165(92), 164(30), 163(29), 139(18), 110(43), 83(42), 82(40), 69(20).

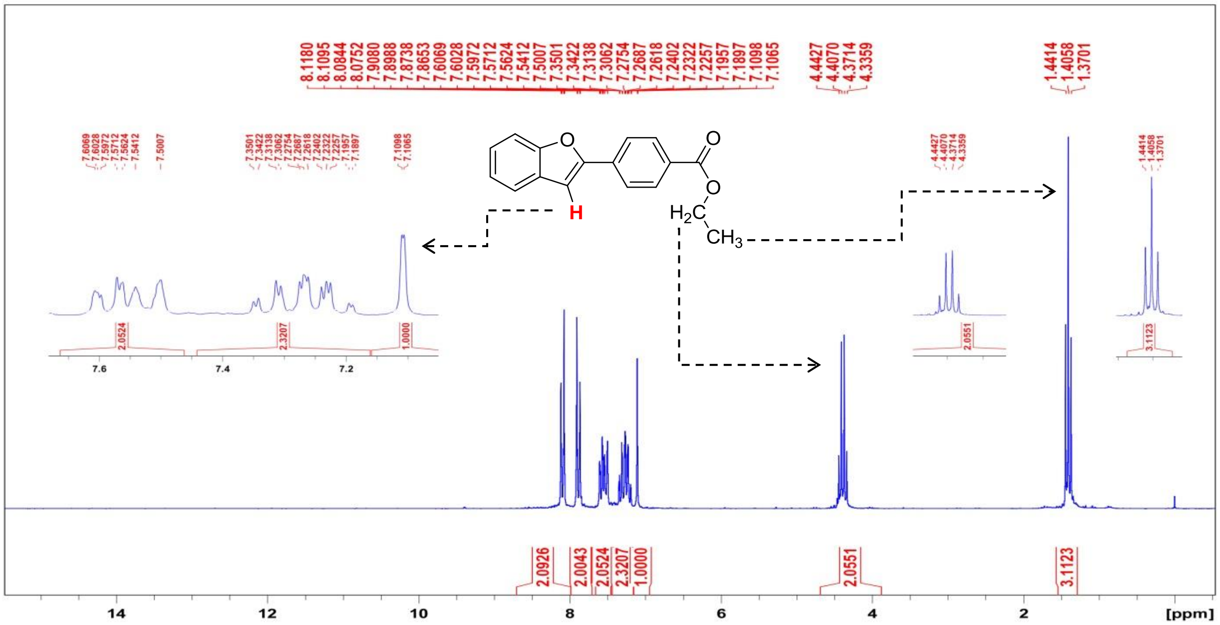

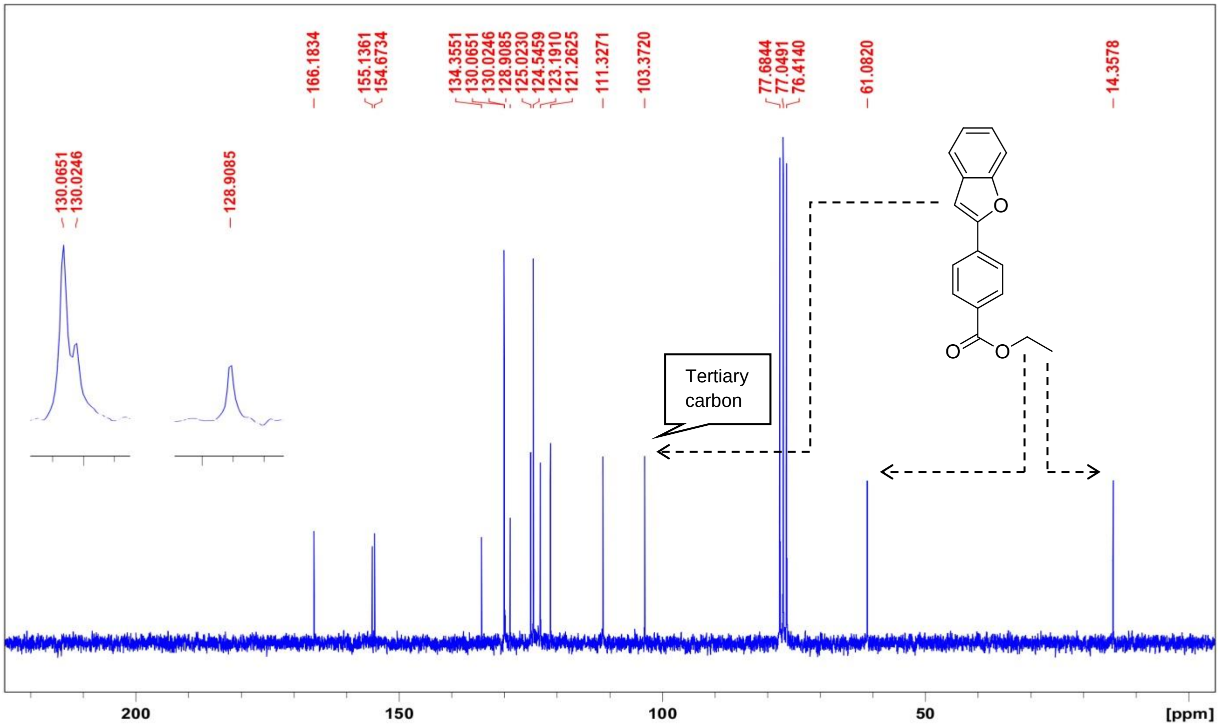

4-(Benzofuran-2-yl)benzaldehyde (2l) [10]: Synthesized according to the general procedure as yellow solid (49 mg product, 44% yield); mp 125-126ºC; **^{1}H NMR (200 MHz, $CDCl_3$)** δ (ppm) = 10.01 (s, 1H), 8.06 – 7.88 (m, 4H), 7.67 – 7.05 (m, 2H), 7.41 – 7.20 (m, 2H), 7.18 (d, *J* = 0.8 Hz, 1H). **^{13}C NMR (50 MHz, $CDCl_3$)** δ (ppm) = 191.5, 155.3, 154.2, 135.8, 135.8, 130.3(2C), 128.8, 125.4, 125.1(2C), 123.3, 121.4, 111.4, 104.3. **MS analyst, m/z (Int.)**: 222(100), 221(48), 193(17), 165(57), 164(18), 163(17), 139(14), 96(39), 82(24), 69(16).

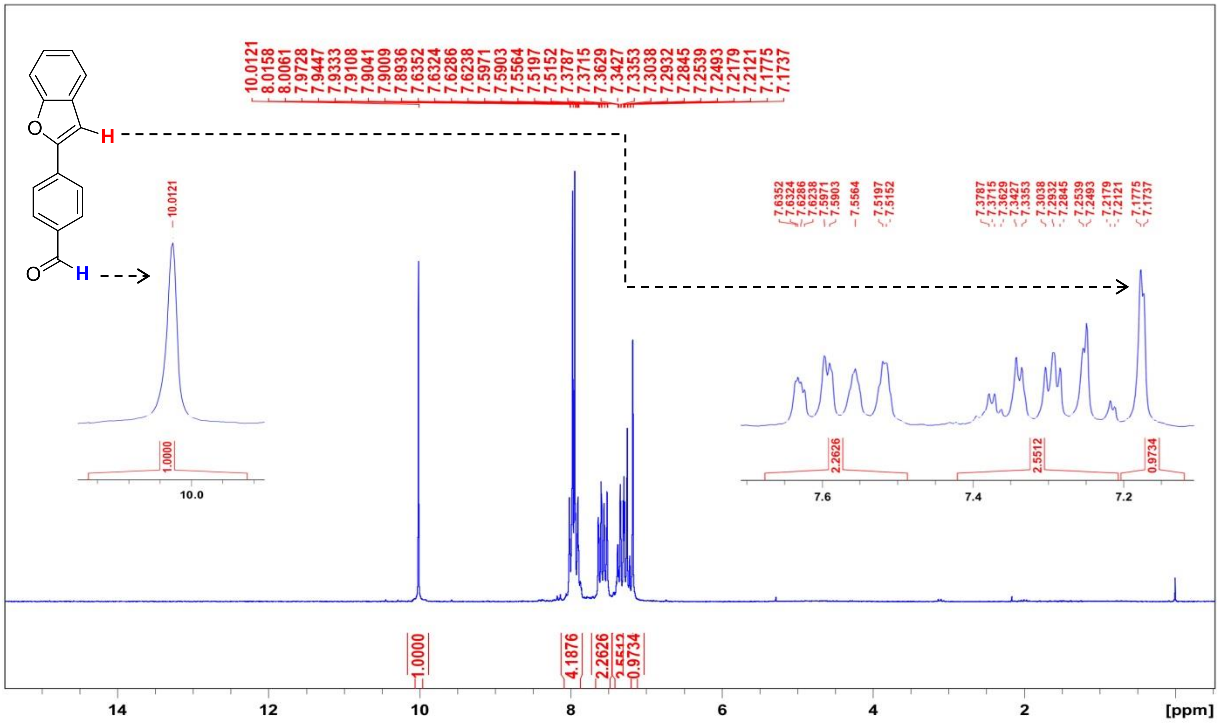

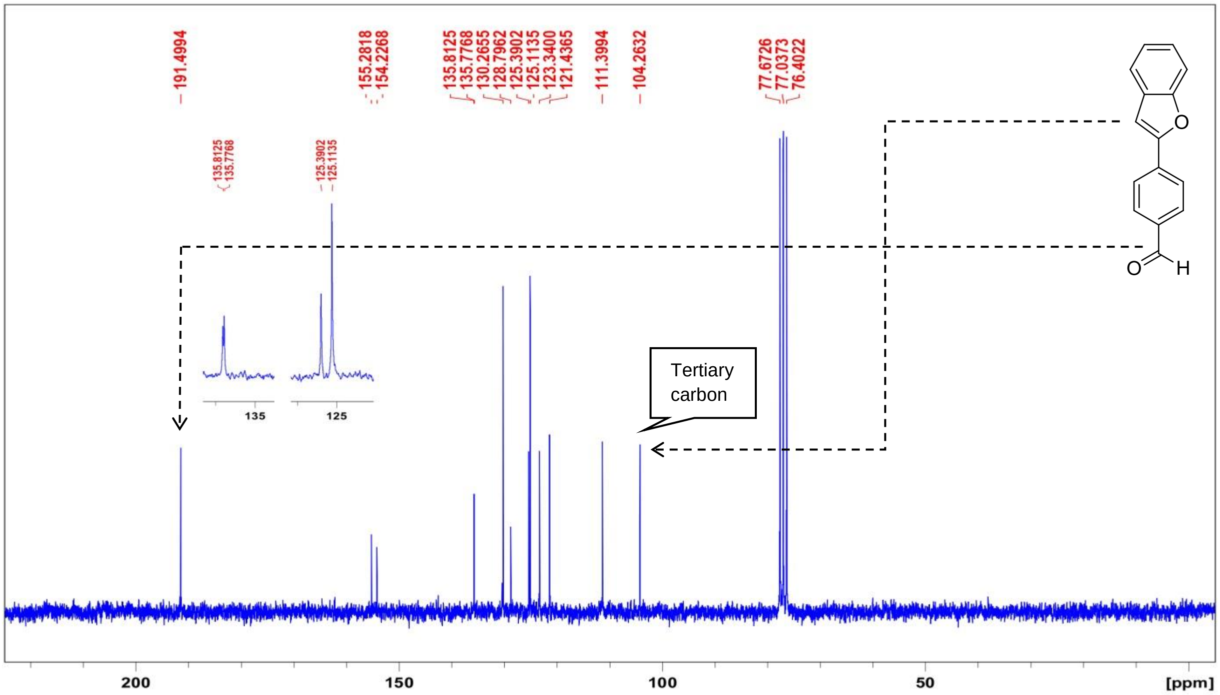

4-(Benzofuran-2-yl)benzonitrile (2m) [11]: Synthesized according to the general procedure as colorless solid (37 mg product, 34% yield); mp 143-145ºC (Lit 145-146ºC); **^{1}H NMR (200 MHz, CDCl$_3$)** δ (ppm) = 7.96 – 7.91 (m, 2H), 7.73 – 7.69 (m, 2H), 7.64 – 7.51 (m, 2H), 7.42 – 7.20 (m, 2H), 7.17 (d, *J* = 0.8 Hz, 1H). **^{13}C NMR (50 MHz, CDCl$_3$)** δ (ppm) = 155.2, 153.5, 134.4, 132.6(2C), 128.7, 125.6, 125.1(2C), 123.4, 121.5, 118.7, 111.5, 111.4, 104.3. **MS analyst, m/z (Int.)**: 219(100), 204(14), 190(53), 164(12).

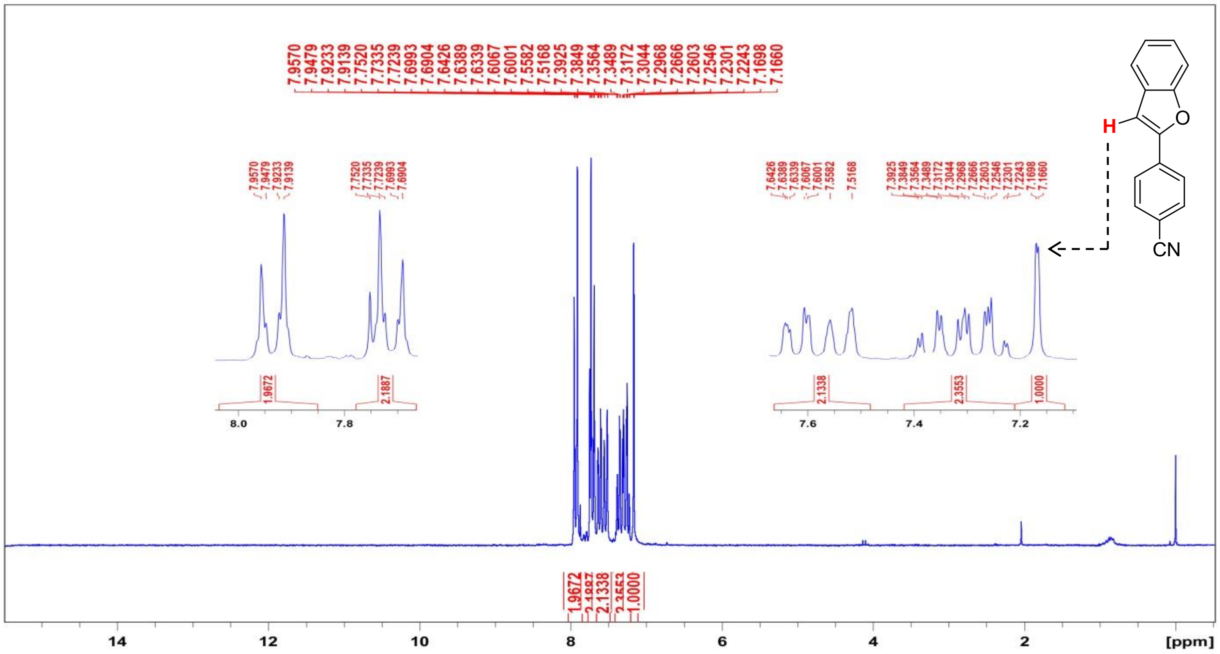

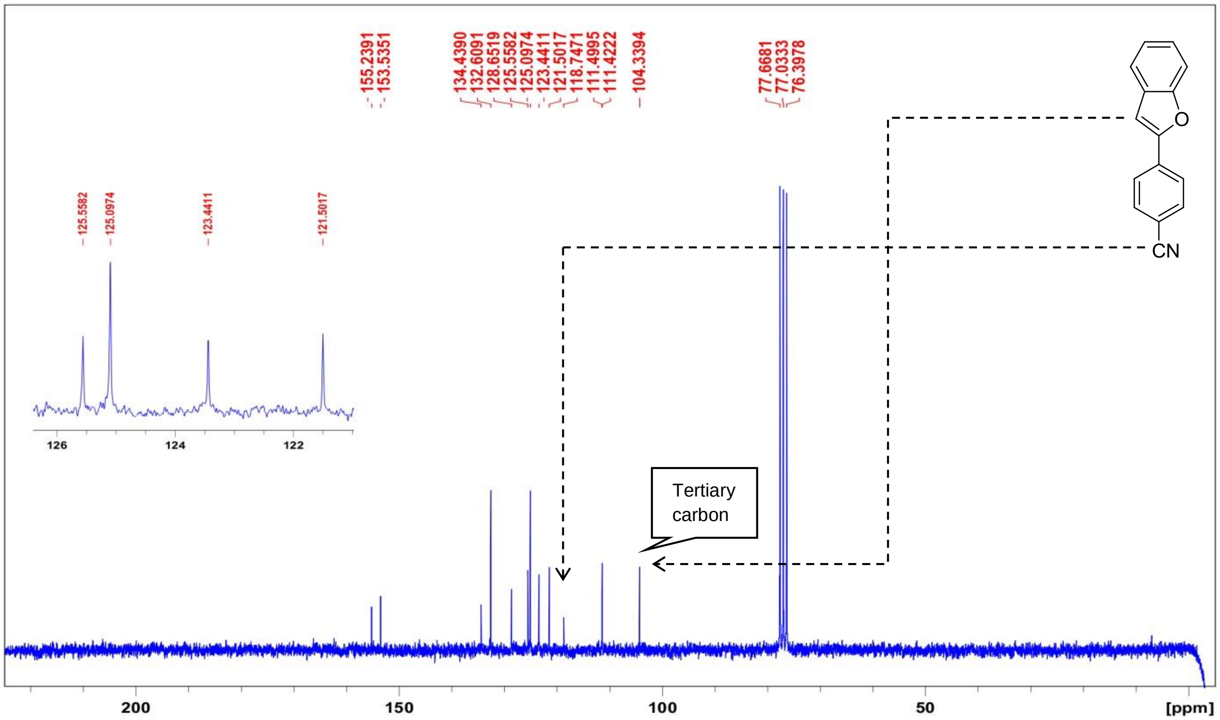

2-(4-Nitrophenyl)benzofuran (2n) [12]: Synthesized according to the general procedure as orange solid (26 mg product, 22% yield); mp 182-184ºC (Lit. 182-183ºC); **^{1}H NMR (200 MHz, $CDCl_3$)** δ (ppm) = 8.31 – 8.26 (m, 2H), 8.00 – 7.95 (m, 2H), 7.66 – 7.53 (m, 2H), 7.41 – 7.23 (m, 2H), 7.22 (d, *J* = 0.6 Hz, 1H). **^{13}C NMR (50 MHz, $CDCl_3$)** δ (ppm) = (ppm) = 155.4, 153.2, 147.2, 136.2, 128.6, 125.8, 125.2(2C), 124.3(2C), 123.5, 121.6, 111.5, 105.1. **MS analyst, m/z (Int.)**: 239(100), 209(43), 193(26), 181(30), 165(97), 164(29), 163(35), 139(19).

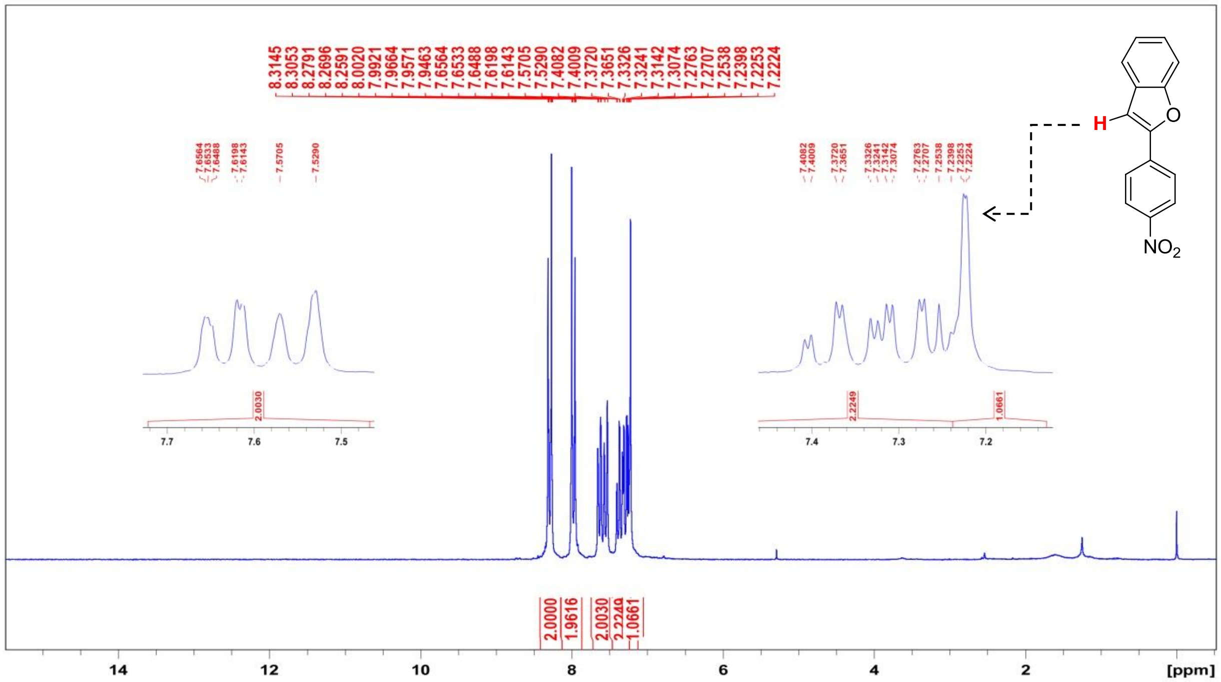

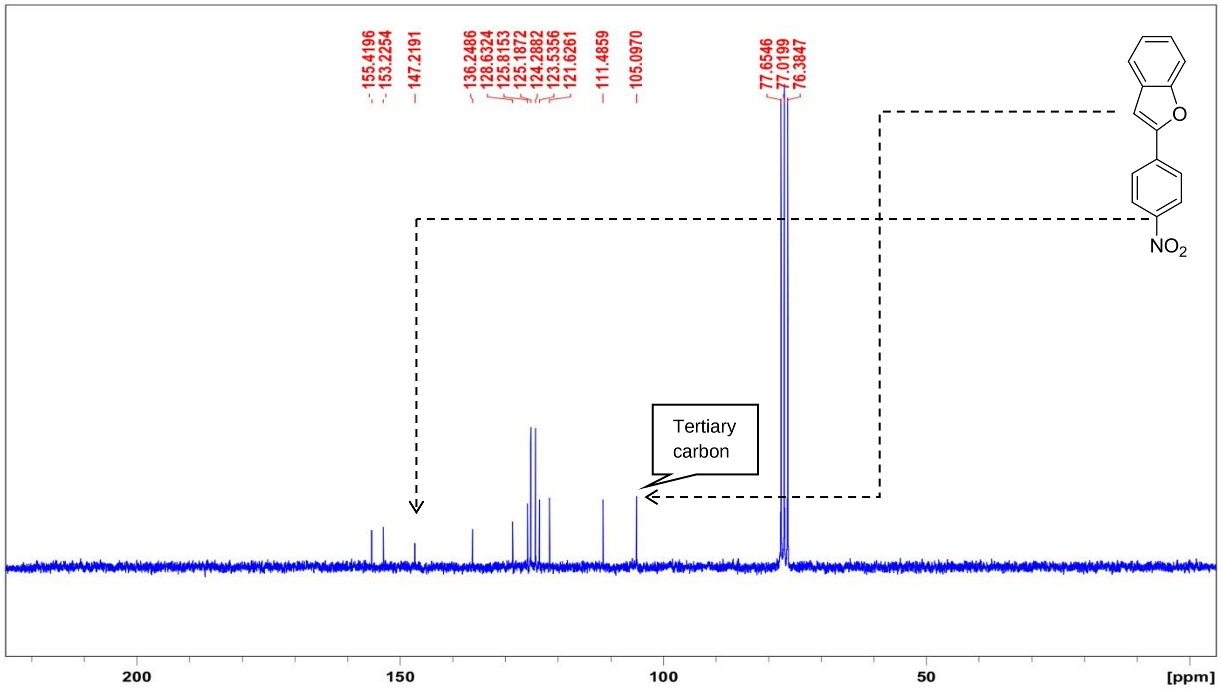

2-Phenyl-benzo[*b*]thiophene (3a) [13]: Synthesized according to the general procedure as colorless solid (45 mg product, 43% yield); mp. 160-161ºC (Lit. 164-168ºC) **^{1}H NMR (200 MHz, $CDCl_3$)** δ (ppm) = 7.84-7.67 (m, 4H), 7.53 (s, 1H), 7.46-7.29 (m, 5H). **^{13}C NMR (50 MHz, $CDCl_3$)** δ (ppm) = 144.3, 140.7, 139.5, 134.3, 129.0, 128.3, 126.5, 124.5 124.3, 123.6, 122.3, 119.5. **MS analyst, m/z (Int.)**: 211(16), 210(100), 209(12), 208(14), 165(23).

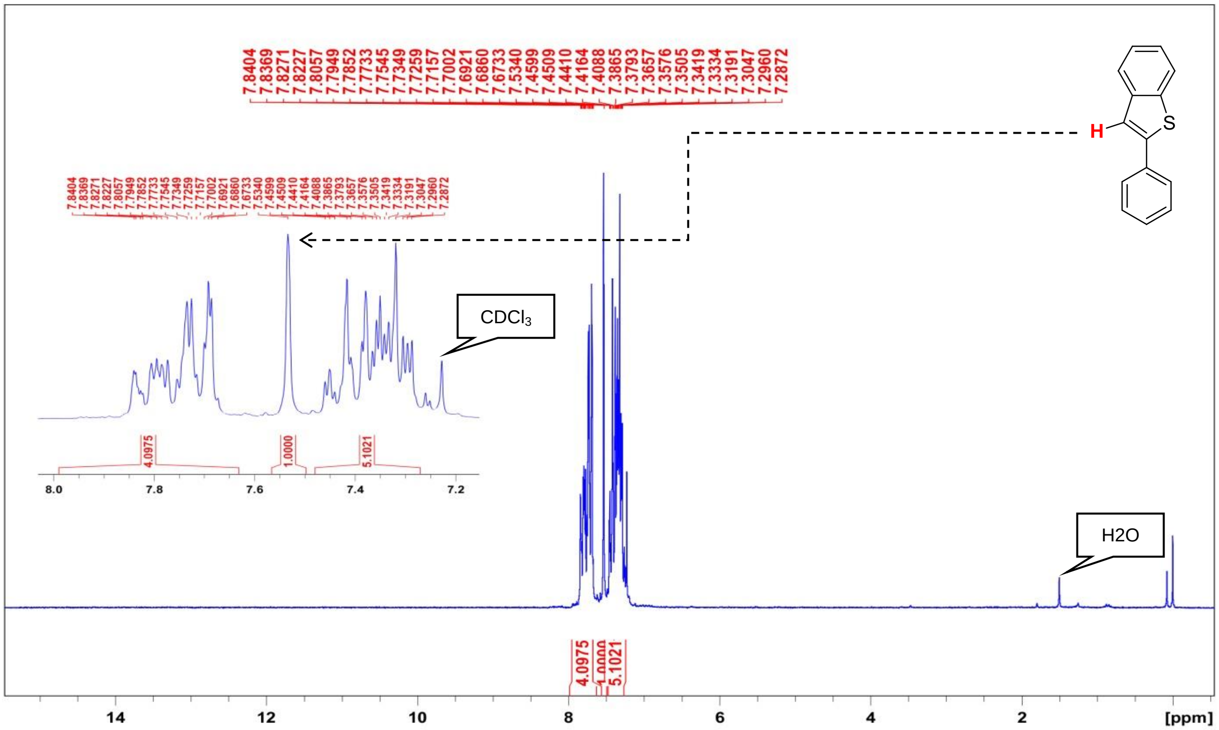

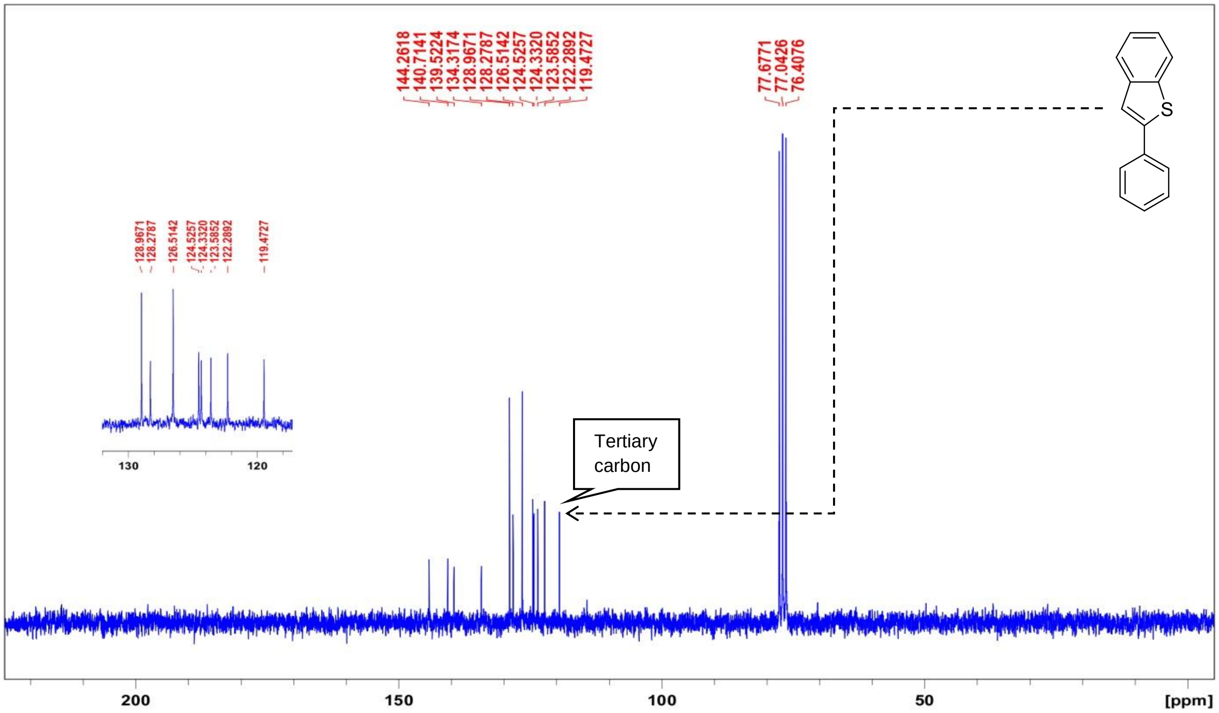

2-(4-Methylphenyl)-benzo[*b*]thiophene (3c) [14]: Synthesized according to the general procedure as colorless solid (38 mg product, 34% yield); mp. 164-165ºC. (Lit. 166-168ºC). **^{1}H NMR (200 MHz, $CDCl_3$)** δ (ppm) = 7.83-7.72 (m, 2H), 7.62-7.58 (m, 2H), 7.49 (s, 1H), 7.34-7.20 (m, 4H), 2.38 (s, 3H). **^{13}C NMR (50 MHz, $CDCl_3$)** δ (ppm) = 144.4, 140.8, 139.3, 138.3, 131.5, 129.6, 126.4, 124.4, 124.1, 123.4, 122.2, 118.9, 21.3. **MS analyst, m/z (Int.)**: 225(18), 224(100), 223(32), 221(13), 208(7).

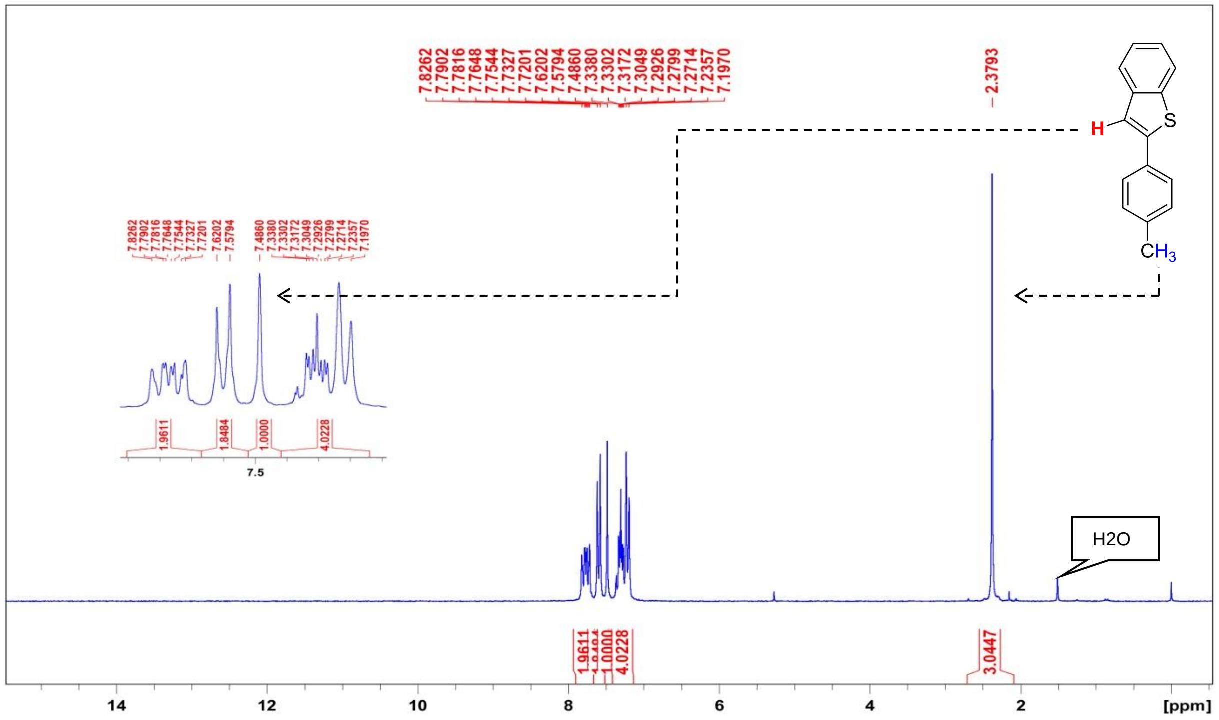

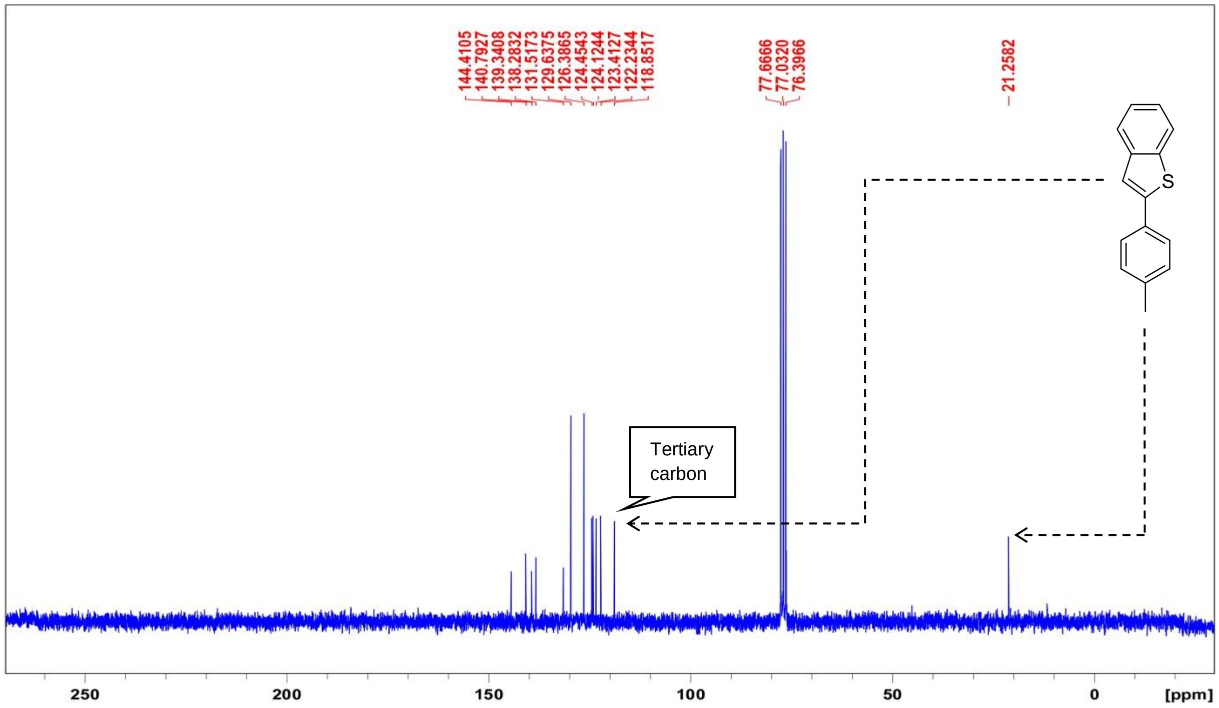

2-(4-Methoxyphenyl)-benzo[*b*]thiophene (3d) [13]: Synthesized according to the general procedure as colorless solid (64 mg, product 53% yield); mp. 193-195ºC (Lit 188-190ºC). **^{1}H NMR (200 MHz, CDCl$_3$)** δ (ppm) = 7.82-7.62 (m, 4H), 7.42 (s, 1H), 7.37-7.24 (m, 2H), 6.97-6.93 (m, 2H), 3.85 (s, 3H). **^{13}C NMR (100 MHz, benzene-d$_6$)** δ (ppm) = 160.1, 141.3, 139.7, 139.5, 127.9, 127.7, 124.5, 124.1, 123.4, 122.3, 118.6, 114.5, 54.6. **MS analyst, m/z (Int.)**: 240(100), 225(67), 197(46), 165(22), 152(16).

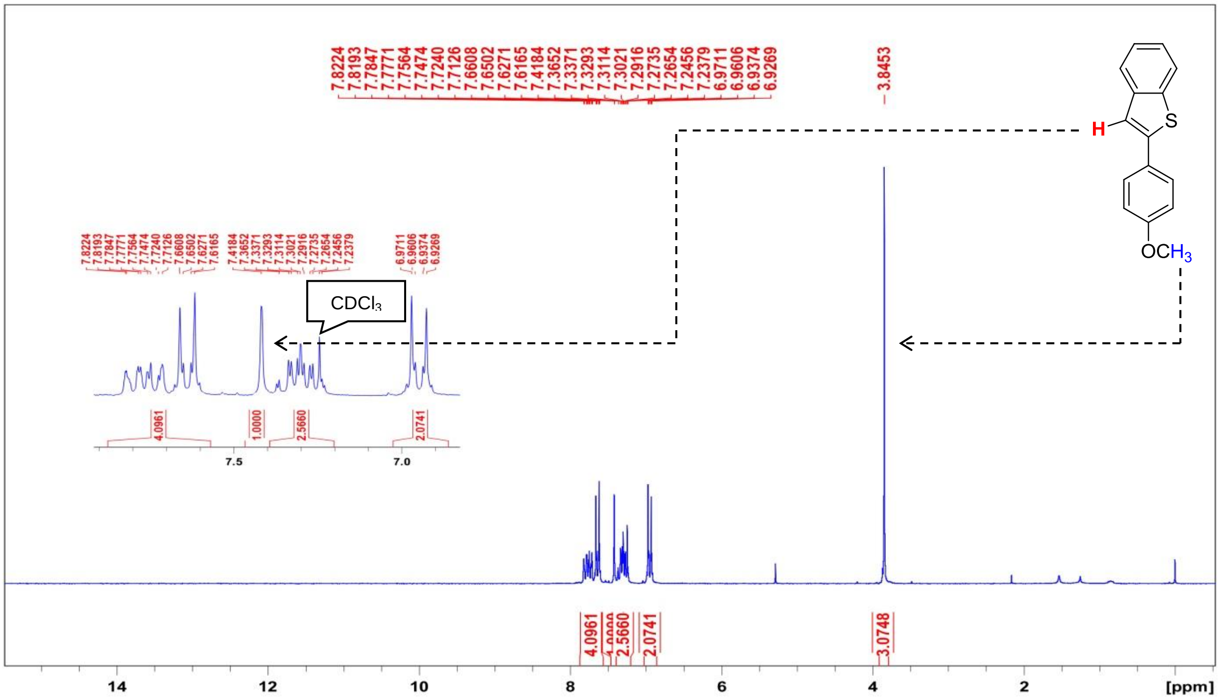

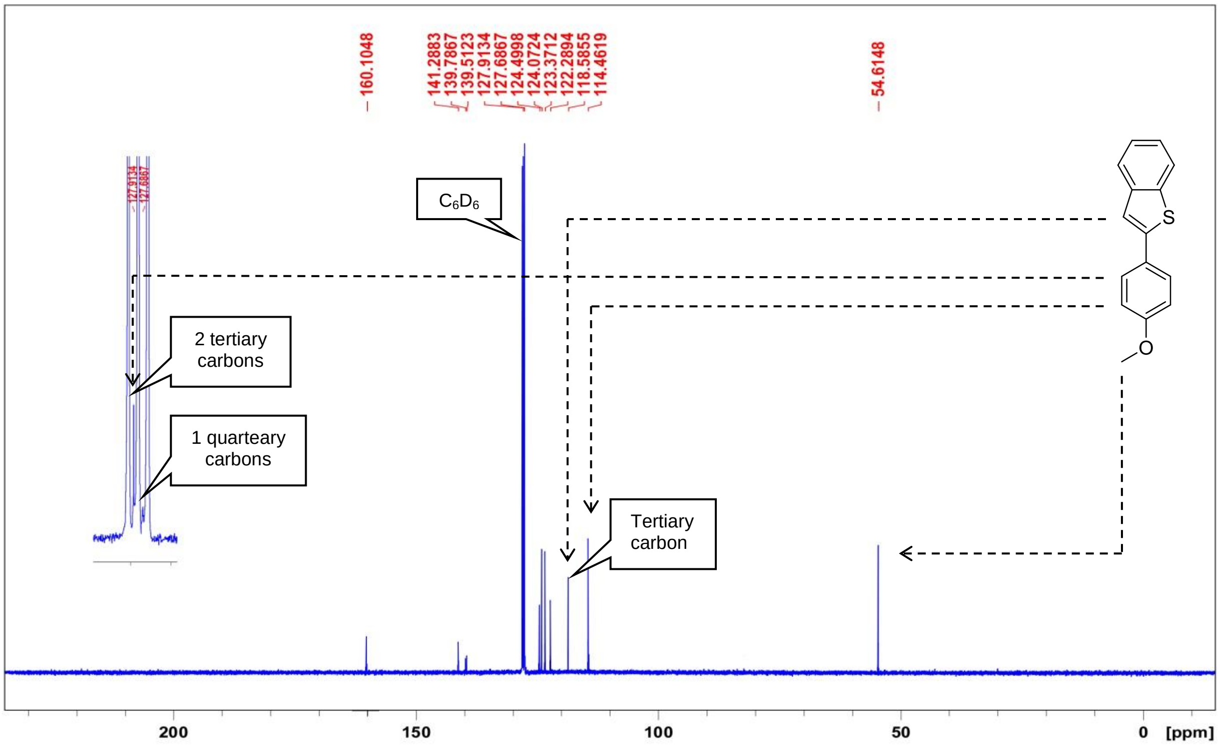

2-(4-Chlorophenyl)-benzo[b]thiophene (3e) [14]: Synthesized according to the general procedure as colorless solid (71 mg product, 58% yield); mp. 200-201ºC (Lit. 190-192ºC). **^{1}HNMR (200 MHz, $CDCl_3$)** δ (ppm) = 7.83-7.73 (m, 2H), 7.63-7.57 (m, 2H), 7.49 (s, 1H) , 7.39-7.23 (m, 4H). **^{13}C NMR (50 MHz, $CDCl_3$)** δ (ppm) = 142.8, 140.6, 139.5, 134.1, 132.8, 129.1, 127.6, 124.6, 123.7, 122.3, 119.9. **MS analyst, m/z (Int.)**: 246(46), 244(100), 208(13), 165(16), 122(14).

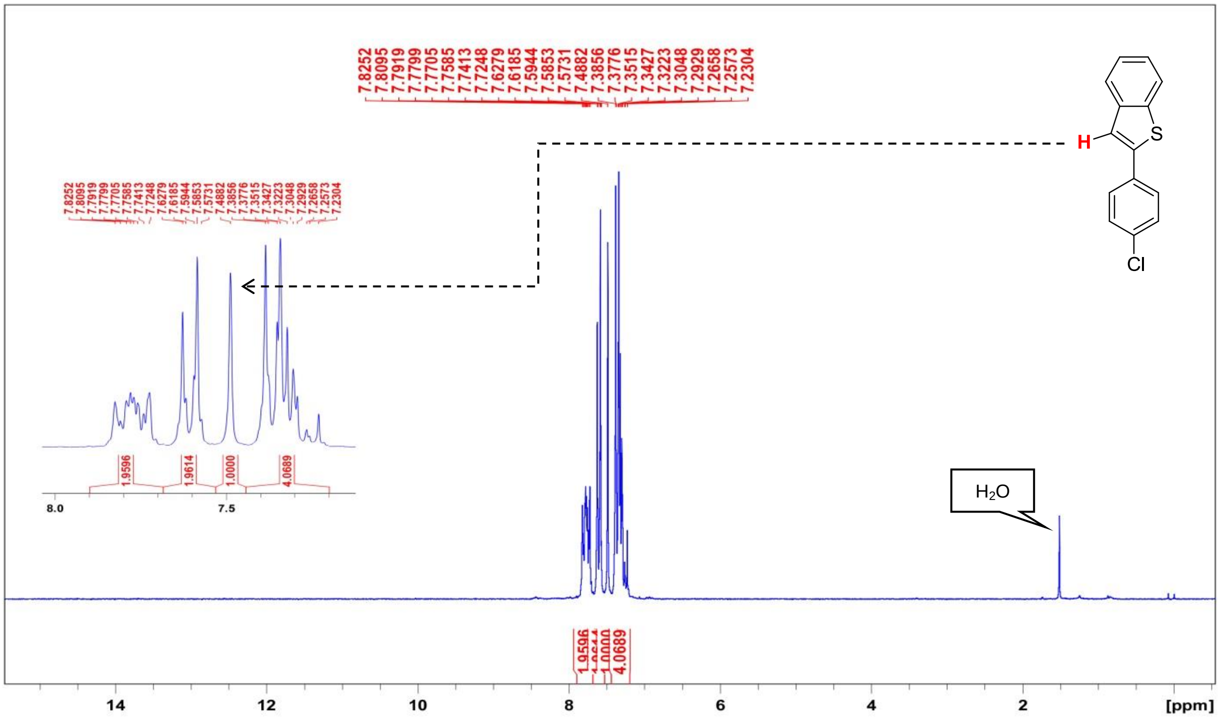

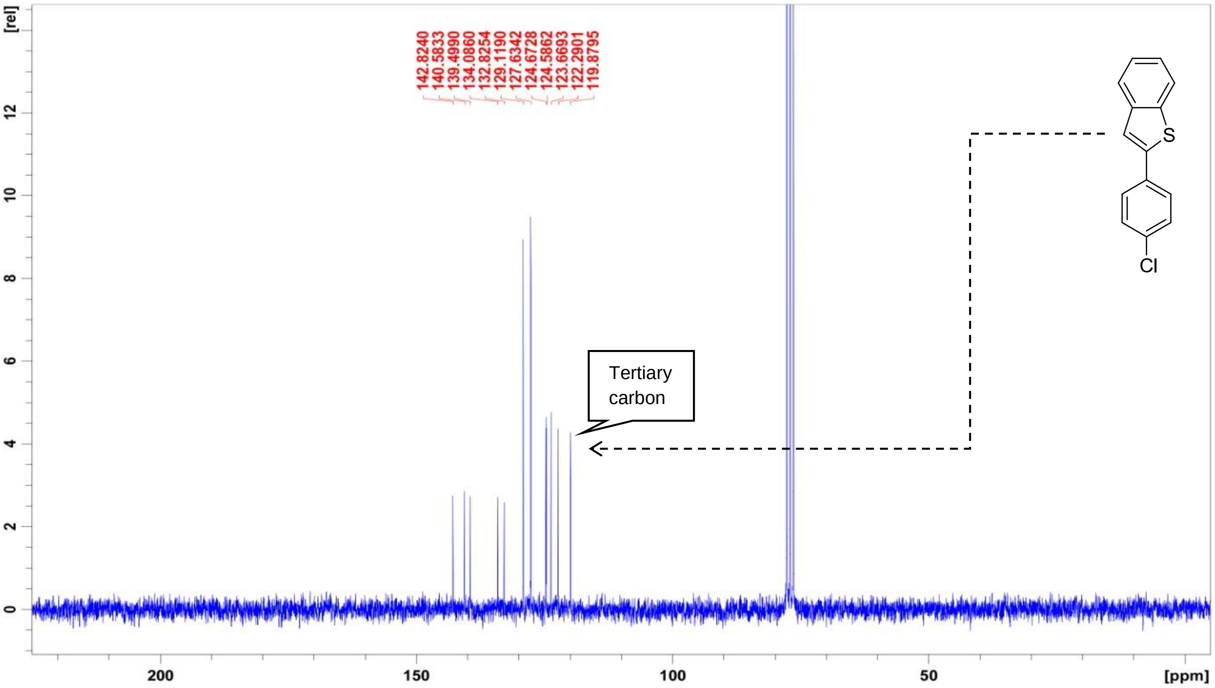

2-(4-Fluorophenyl)-benzo[b]thiophene (3f) [15]: Synthesized according to the general procedure as colorless solid (40 mg product, 35% yield); mp. 183-184ºC. **^{1}H NMR (200 MHz, $CDCl_3$)** δ (ppm) = 7.85-7.63 (m, 4H), 7.47 (s, 1H), 7.41-7.28 (m, 2H), 7.18-7.08 (m, 2H). **^{13}CNMR (50 MHz, $CDCl_3$)** δ (ppm) = 162.8 (d, J_{C-F} = 247 Hz), 143.1, 140.7, 139.4, 130.6 (d, J_{C-F} = 3Hz), 128.2 (d, J_{C-F} = 8Hz, 2C), 124.6, 124.4, 123.6, 122.3 119.4, 116.0 (d, J_{C-F} = 22Hz, 2C). **MS analyst, m/z (Int.)**: 229(16), 228(100), 196(13), 183(23), 114(9).

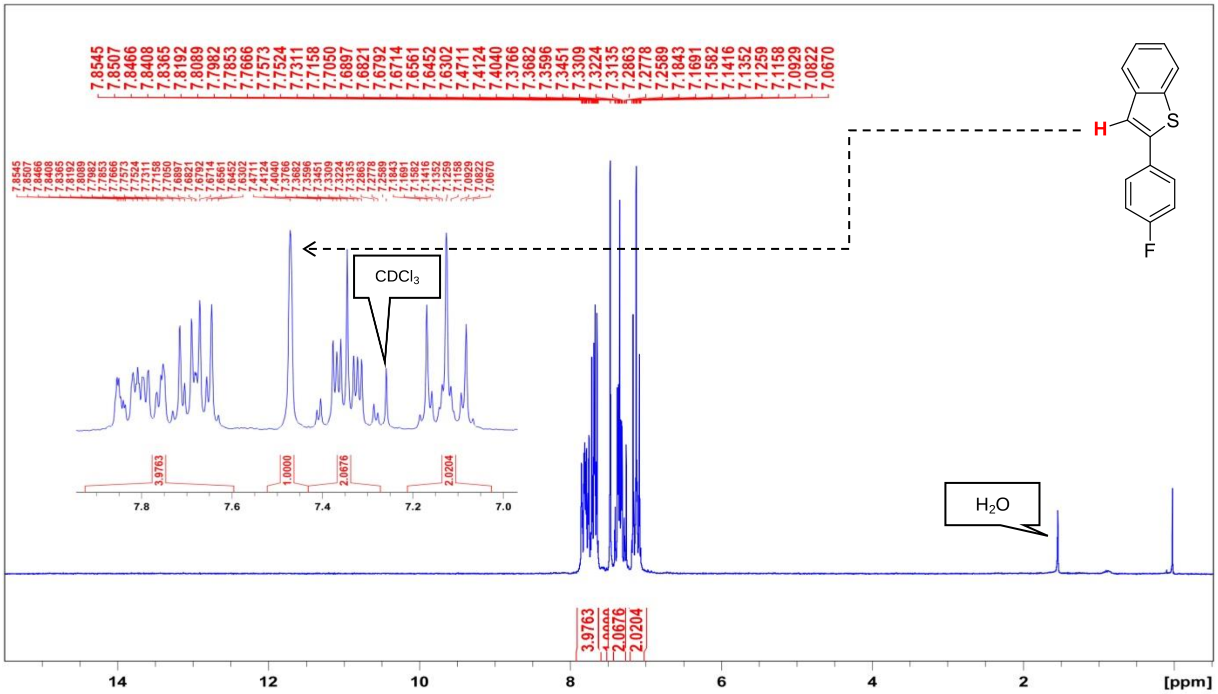

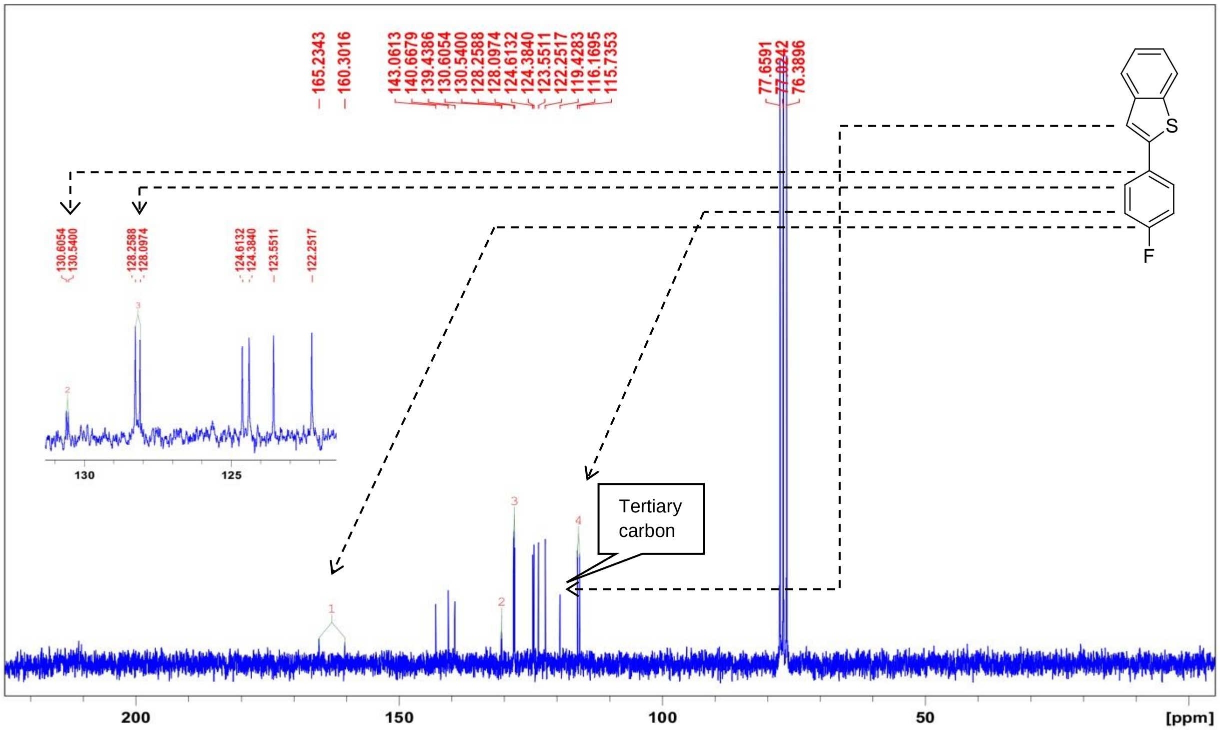

2-(2-Methylphenyl)-benzo[b]thiophene (3h) [16]: Synthesized according to the general procedure as colorless solid (76 mg product, 68% yield); mp. 73-74ºC (Lit. 69-70ºC). **^{1}H NMR (200 MHz, $CDCl_3$)** δ (ppm) = 7.85-7.73 (m, 2H), 7.48-7.46 (m, 1H), 7.43-7.21 (m, 6H), 2.46 (s, 3H). **^{13}C NMR (50 MHz, $CDCl_3$)** δ (ppm) = 143.5, 140.2, 140.1, 136.5, 134.2, 130.8, 130.7, 128.4, 126.0, 124.4, 124.1, 123.5, 123.1, 122.1, 21.1. **MS analyst, m/z (Int.)**: 225(18), 224(100), 223(95), 221(19), 189(13).

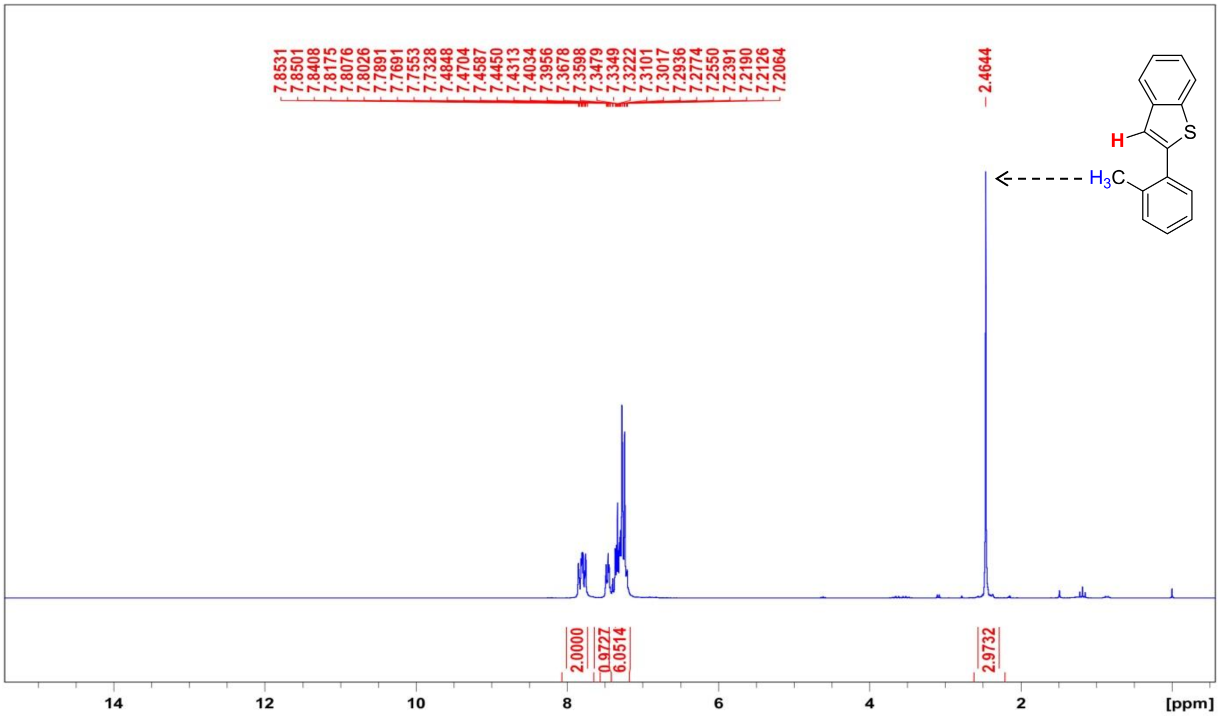

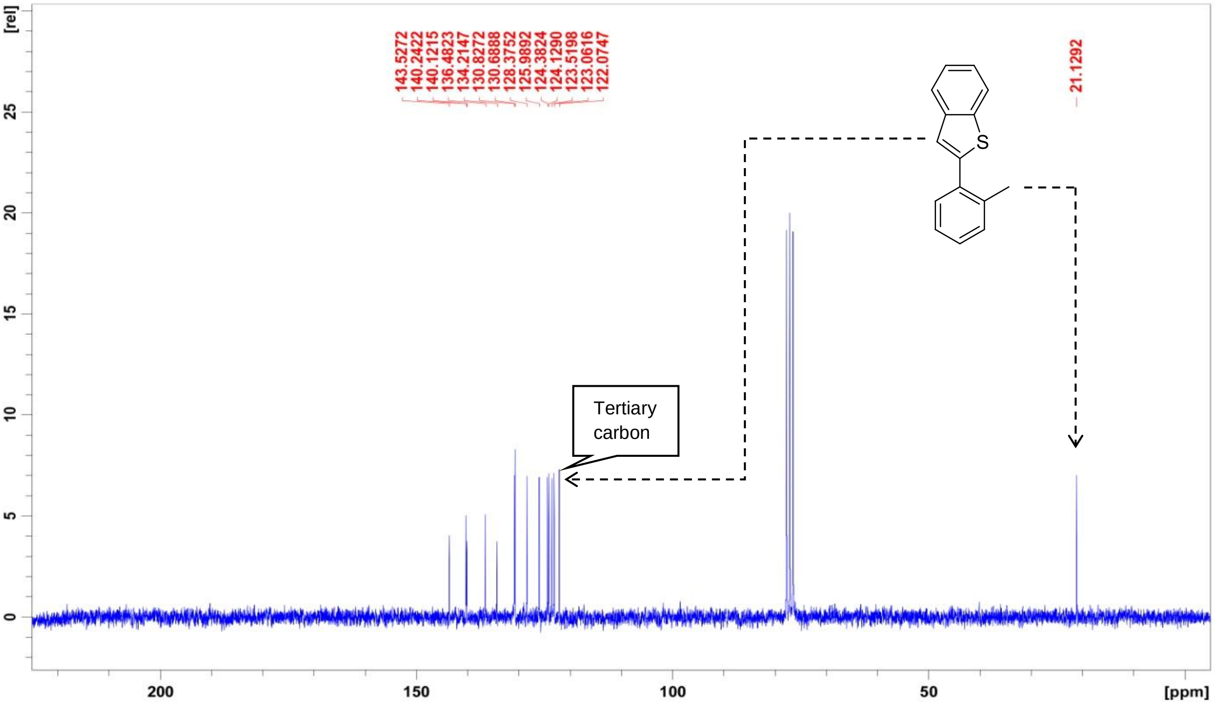

2-(2-Chlorophenyl)-benzo[b]thiophene (3i) [17]: Synthesized according to the general procedure as colorless solid (73 mg product, 60% yield); mp. 75-76ºC. **^{1}H NMR (200 MHz, $CDCl_3$)** δ (ppm) = 7.87-7.79 (m, 2H), 7.61-7.57 (m, 2H), 7.53-7.48 (m, 1H), 7.41-7.24 (m, 4H). **^{13}C NMR (50 MHz, $CDCl_3$)** δ (ppm) = 140.3, 140.2, 139.9, 133.2, 132.8, 131.9, 130.6, 127.0, 124.6, 124.6, 124.5, 124.4, 123.9, 122.0. **MS analyst, m/z (Int.)**: 246(28), 244(100), 208(23), 165(40), 104(19).

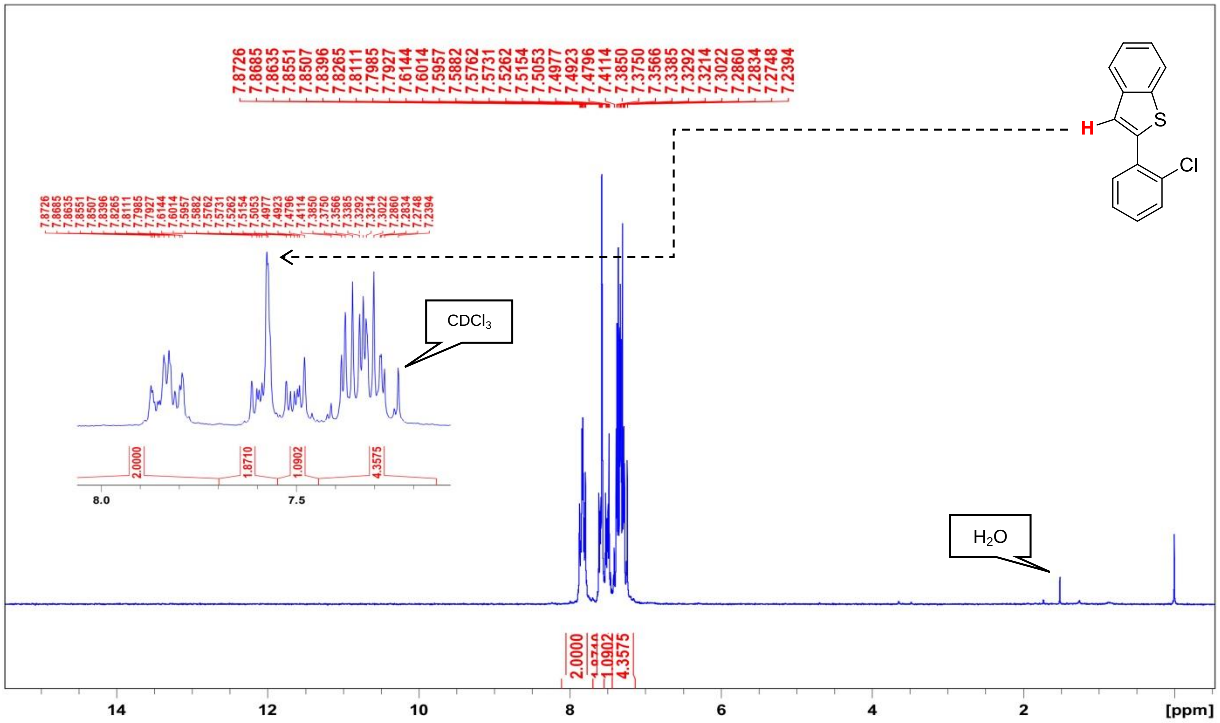

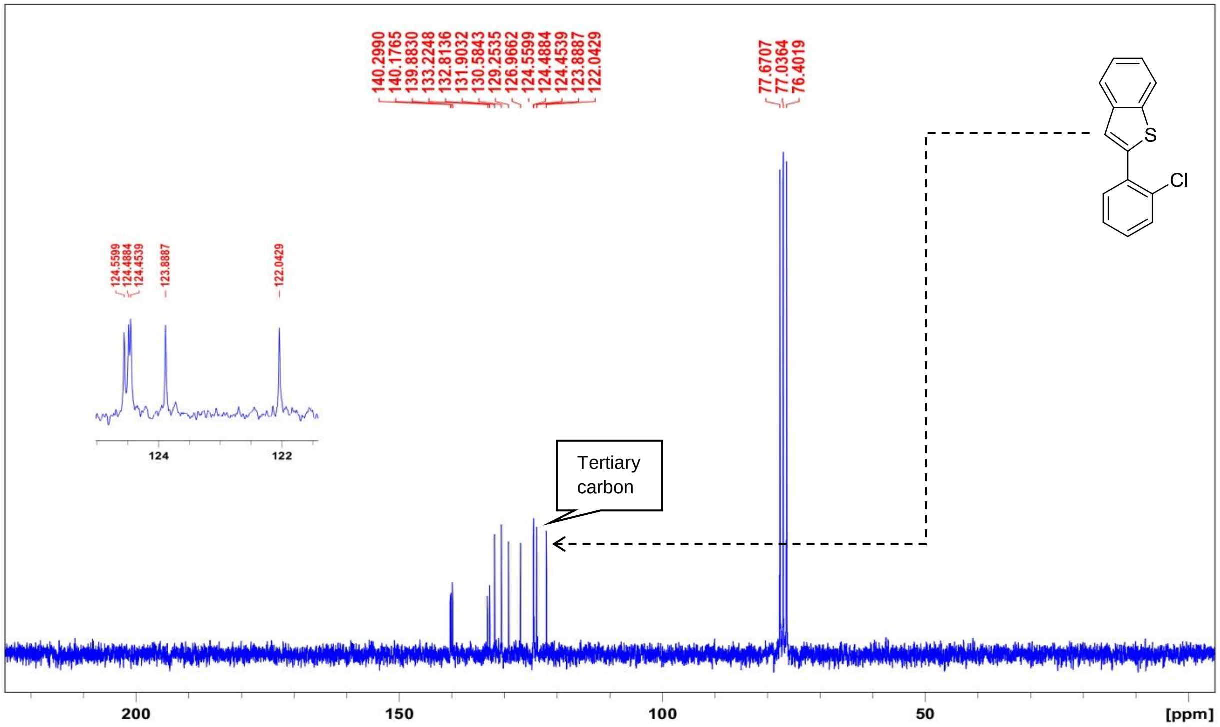

Ethyl 4-(benzo[b]thien-2-yl)-benzoate (3j) [15]: Synthesized according to the general procedure as colorless solid (64 mg product, 45% yield); mp. 171-172 ºC (Lit. 166-168 ºC). **^{1}H NMR (200 MHz, $CDCl_3$)** δ (ppm) = 8.12-8.06 (m, 2H), 7.86-7.73 (m, 4H), 7.64 (s, 1H), 7.38-7.33 (m, 2H), 4.41 (q, *J* = 7.1 Hz, 2H), 1.42 (t, *J* = 7.1 Hz, 3H). **^{13}C NMR (50 MHz, $CDCl_3$)** δ (ppm) = 166.2, 142.8, 140.5, 139.8, 138.4, 130.2, 129.9, 126.1, 124.9, 124.7, 123.9, 122.3, 121.0, 61.1, 14.3. **MS analyst, m/z (Int.)**: 282(100), 237(51), 208(38), 165(80), 104(52).

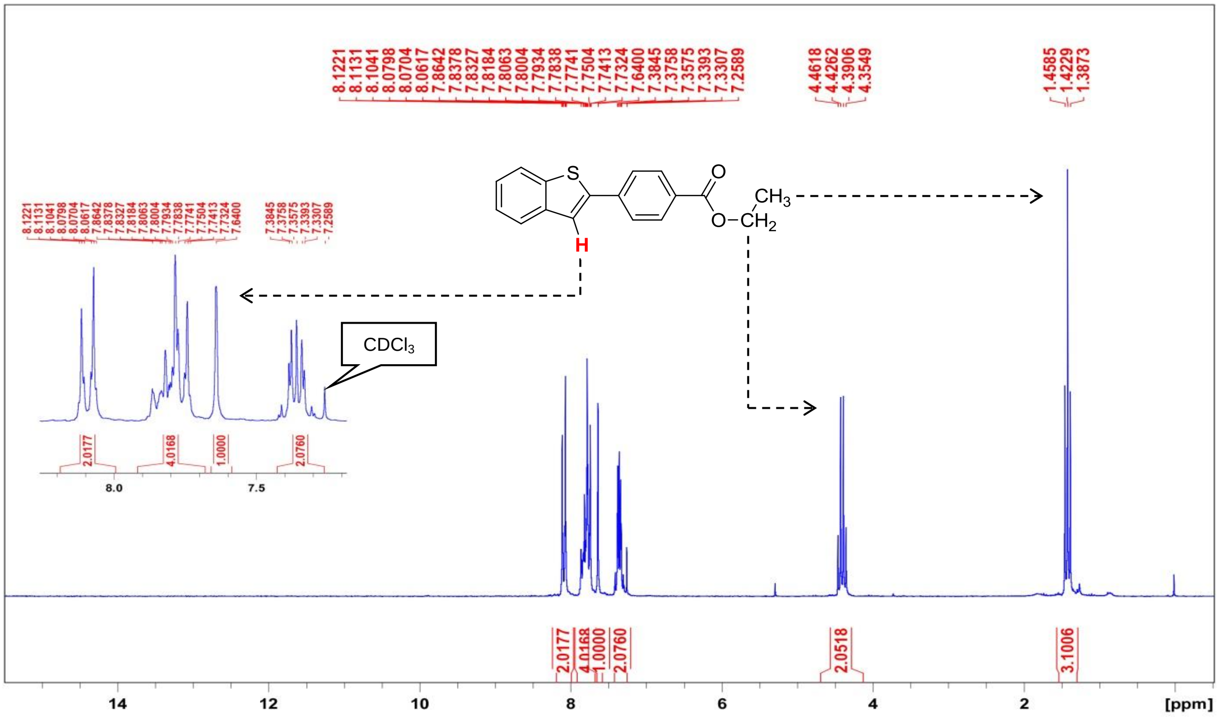

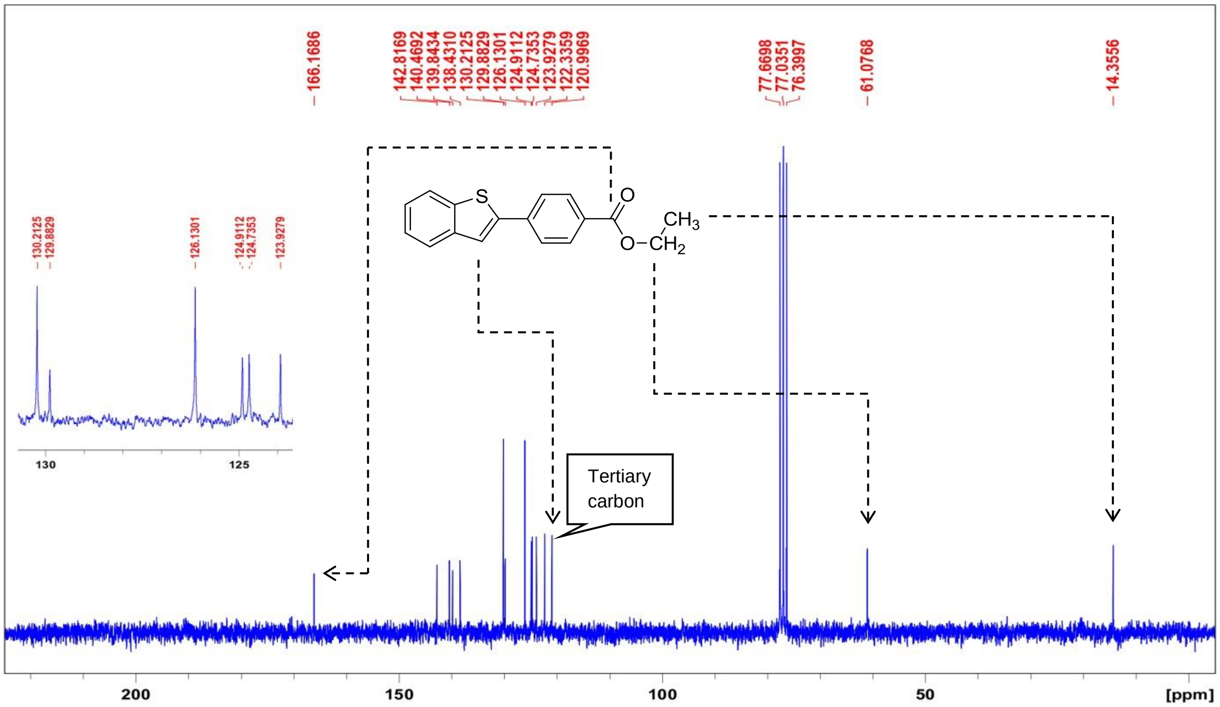

2-(4-Cyanophenyl)-benzo[b**]thiophene (3k)** [14]: Synthesized according to the general procedure as colorless solid (29 mg product, 25% yield); mp. 168-171ºC (Lit. 181-183ºC). **^{1}H NMR (200 MHz, $CDCl_3$)** δ (ppm) = 7.87-7.66 (m, 6H), 7.64 (s, 1H), 7.43-7.33 (m, 2H). **^{13}C NMR (50 MHz, $CDCl_3$)** δ (ppm) = 141.7, 140.3, 140.0, 138.6, 132.7, 126.7, 125.4, 125.0, 124.2, 122.4, 121.8, 118.7, 111.4. **MS analyst, m/z (Int.)**: 236(19), 235(100), 203(10), 190(23), 118(12).

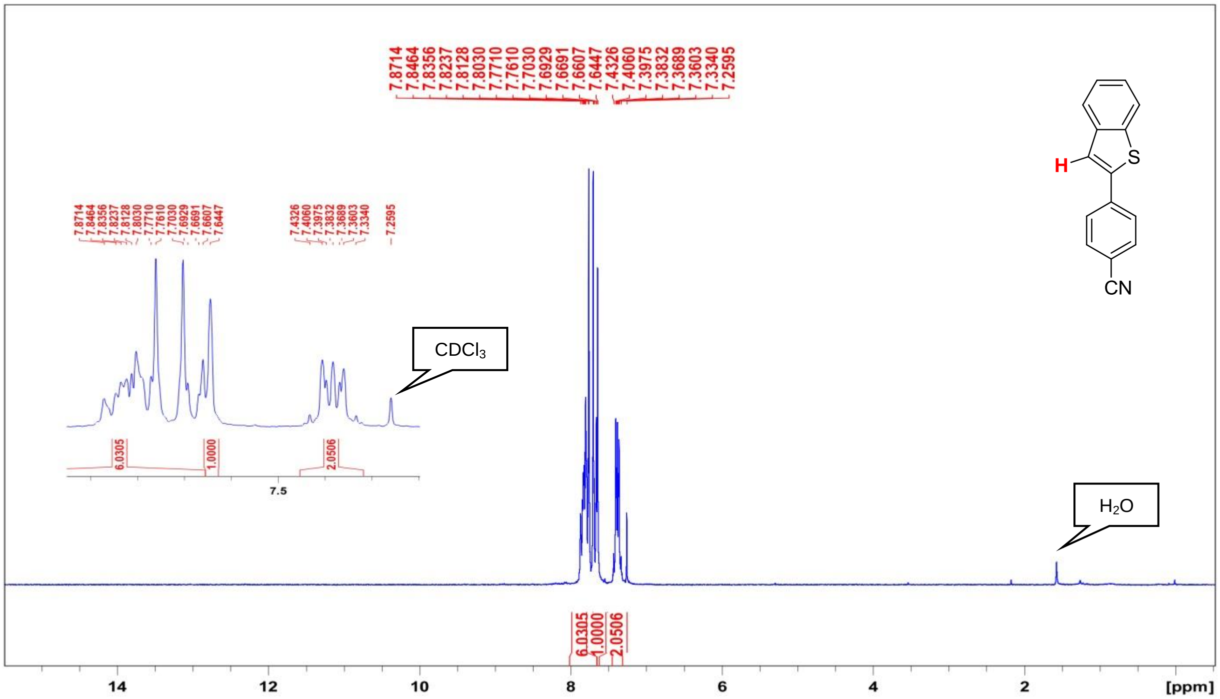

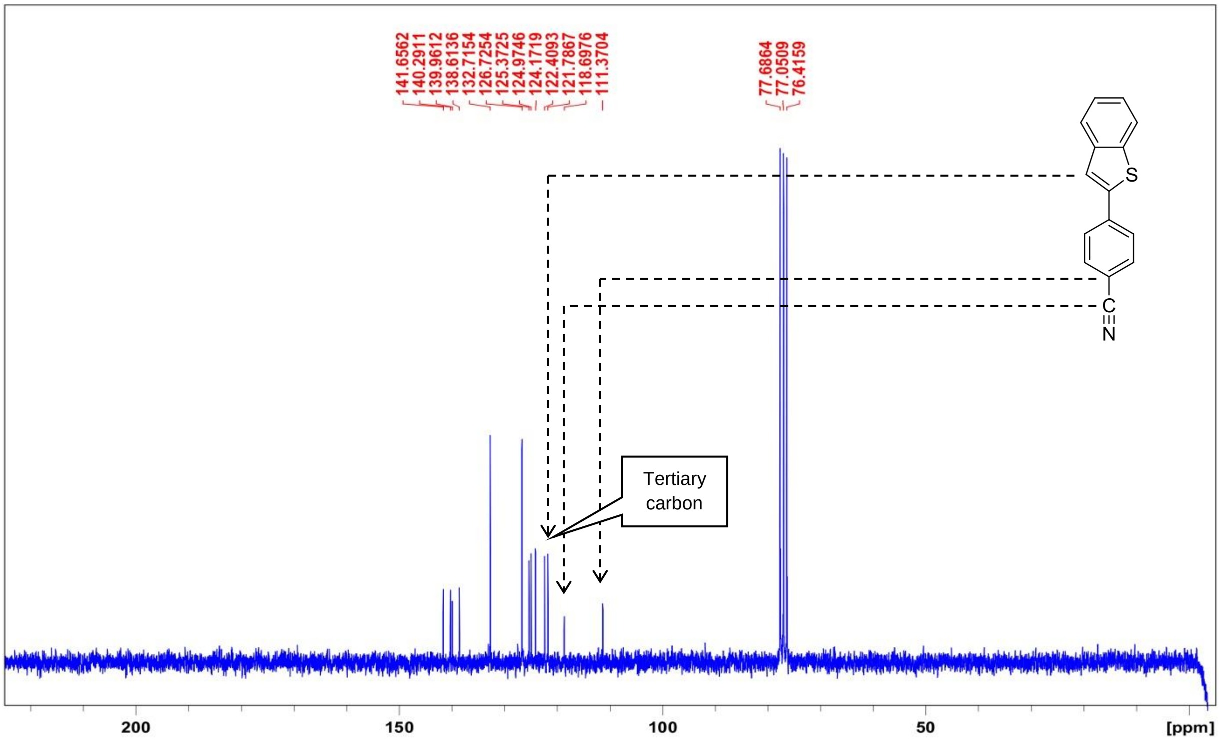

2-(4-Nitrophenyl)-benzo[b]thiophene (3l) [18]: Synthesized according to the general procedure as colorless solid (33 mg product, 26% yield); mp. 213-214ºC (Lit. 201-202ºC). **^{1}H NMR (200 MHz, DMSO)** δ (ppm) = 8.33-8.29 (m, 2H), 8.15 (s, 1H), 8.07-8.02 (m, 3H), 7.94-7.89 (m, 1H), 7.45-7.39 (m, 2H). **^{13}C NMR (50 MHz, DMSO)** δ (ppm) = 146.7, 142.2, 140.4, 140.1, 139.8, 139.4, 126.8, 125.7, 125.2, 124.5, 123.5, 122.6. **MS analyst, m/z (Int.)**: 255(100), 225(29), 209(21), 208 (50), 197 (17), 165 (48).

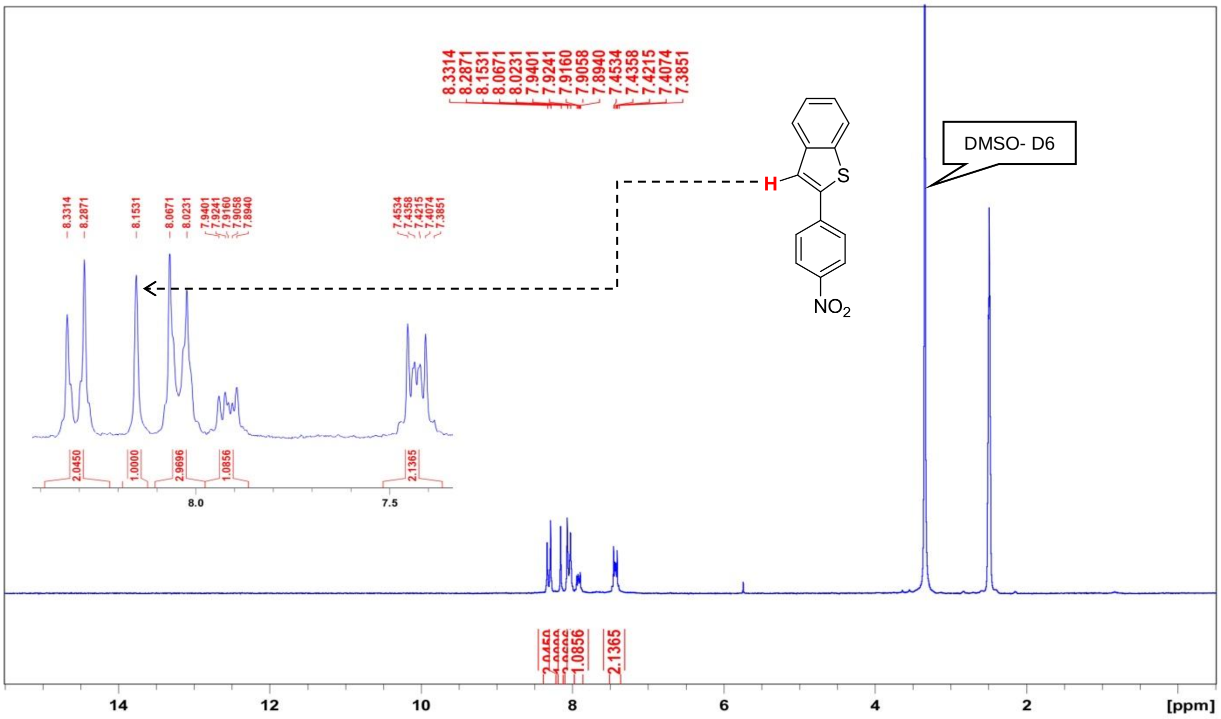

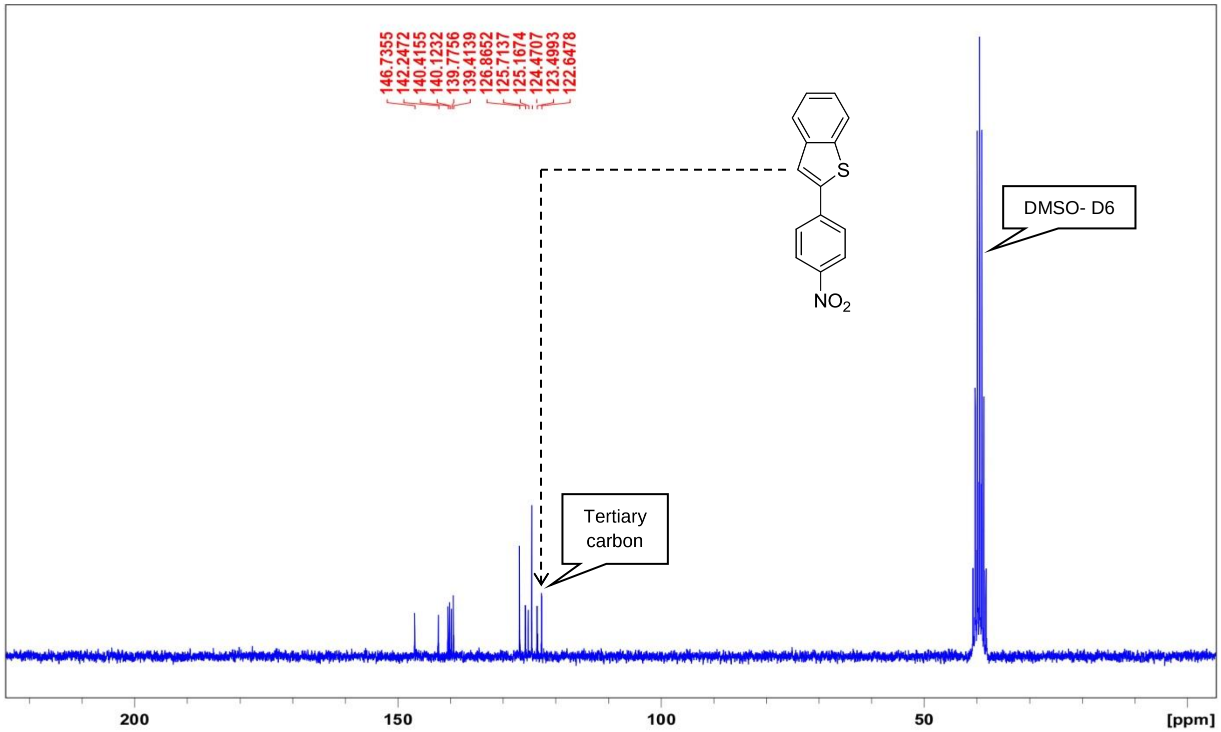

1-Methyl-2-phenyl-1H-indole (3n) [19]: Synthesized according to the general procedure as light purple solid (66 mg product, 64% yield); mp 98-100ºC. **^{1}H NMR (200 MHz, $CDCl_3$)** δ (ppm) = 7.65 – 7.62 (m, 1H), 7.54 – 7.34 (m, 6H), 7.28 – 7.10 (m, 2H), 6.56 (s, 1H), 3.73 (s, 3H). **^{13}C NMR (50 MHz, $CDCl_3$)** δ (ppm) = 141.6, 138.4, 132.9, 129.4(2C), 128.5(2C), 128.0, 127.9, 121.7, 120.5, 119.9, 109.6, 101.7, 31.2. **MS analyst, m/z (Int.)**: 208(17), 207(100), 206(46), 165(12), 102(14).

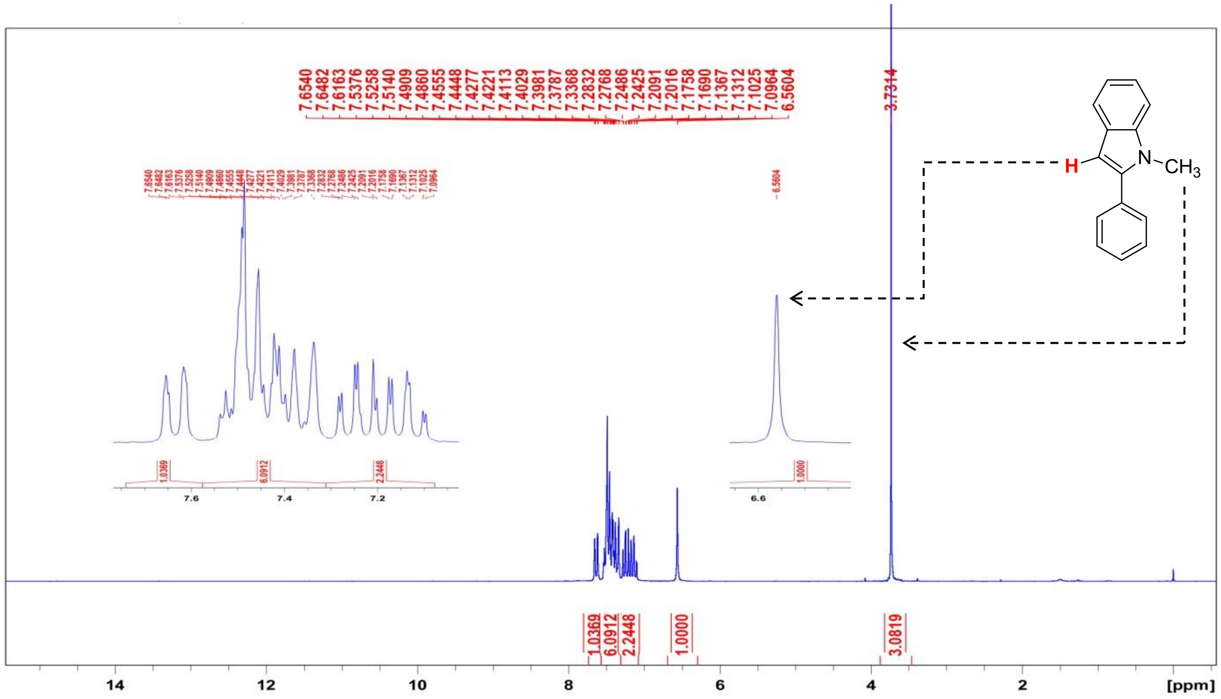

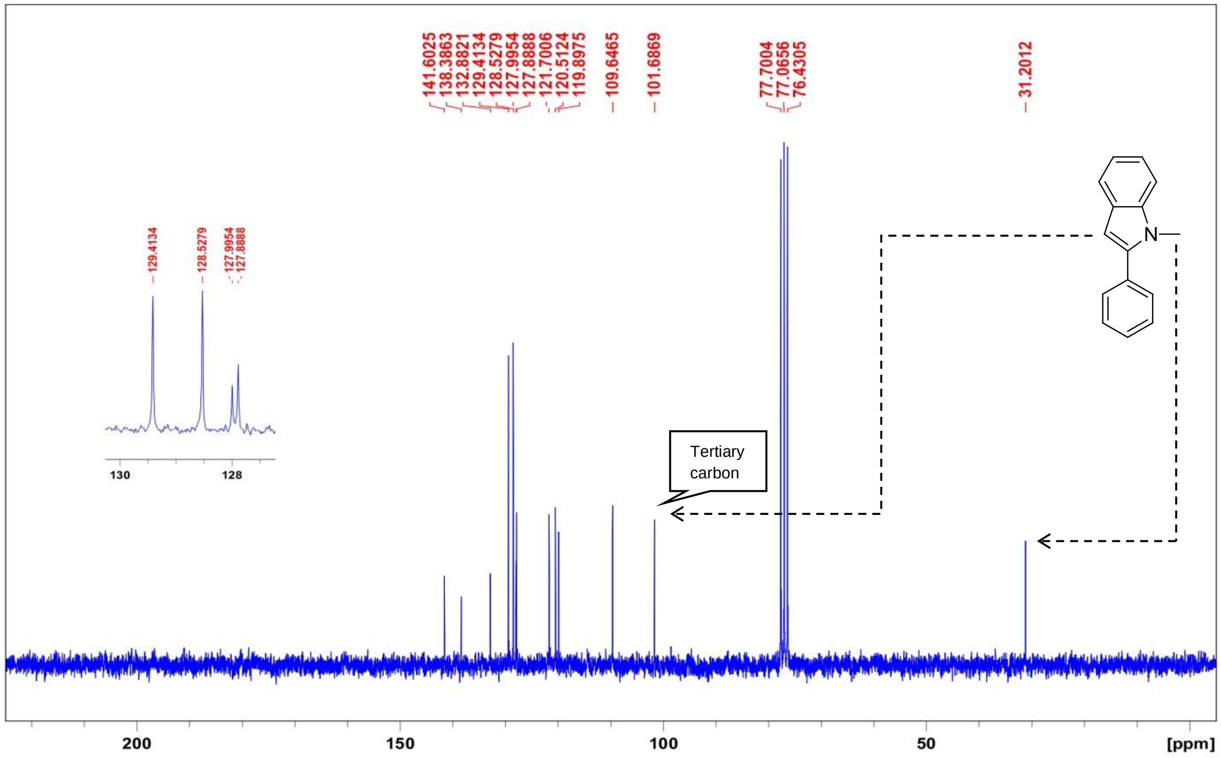

2,3-Diphenylbenzo[b]furan (4a) [20]: Synthesized according to the general procedure (in 0.25 mmol scale) as colorless solid (32 mg product, 47% yield); mp 122-123ºC (Lit. 121ºC). **^{1}H NMR (200 MHz, $CDCl_3$)** δ (ppm) = 7.72 – 7.59 (m, 2H), 7.59 – 7.18 (m, 12H). **^{13}C NMR (50 MHz, $CDCl_3$)** δ (ppm) = 154.0, 150.5, 132.9, 130.7, 130.3, 129.8 (2C), 129.0(2C), 128.4(2C), 128.4, 127.6, 127.0(2C), 124.7, 122.9, 120.0 117.5, 111.1. **MS analyst, m/z (Int.)**: 271(21), 270(100), 269(23), 255(12), 241(30), 239(31), 207(32), 165(10), 134(15), 119(15).

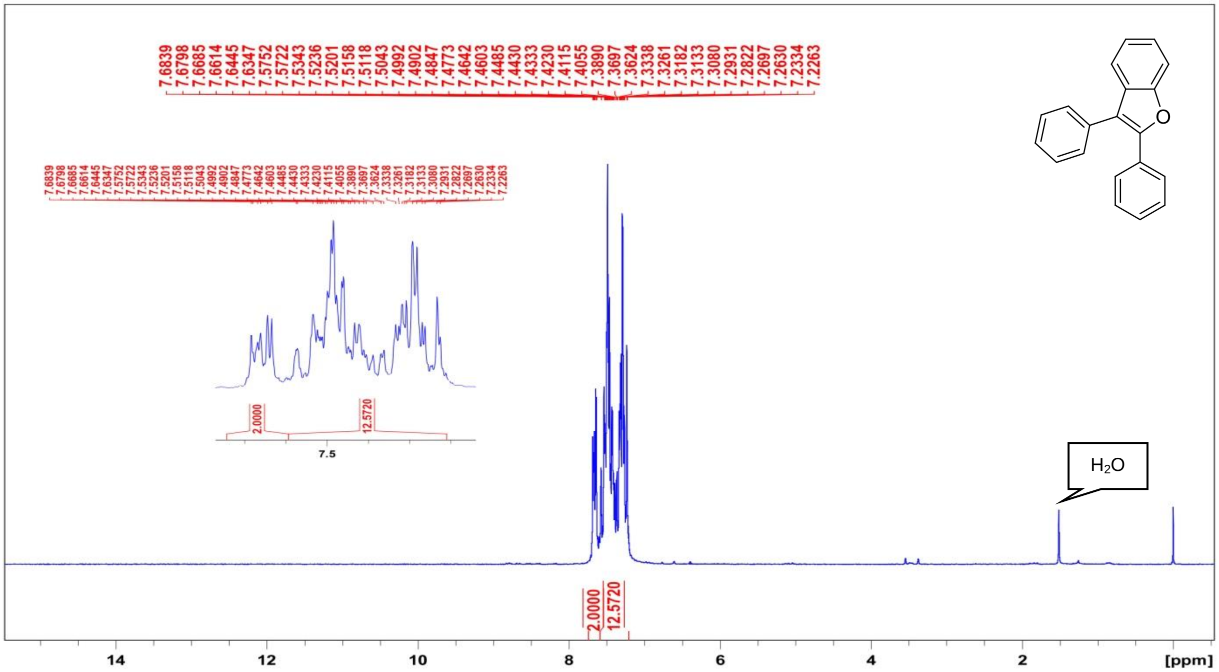

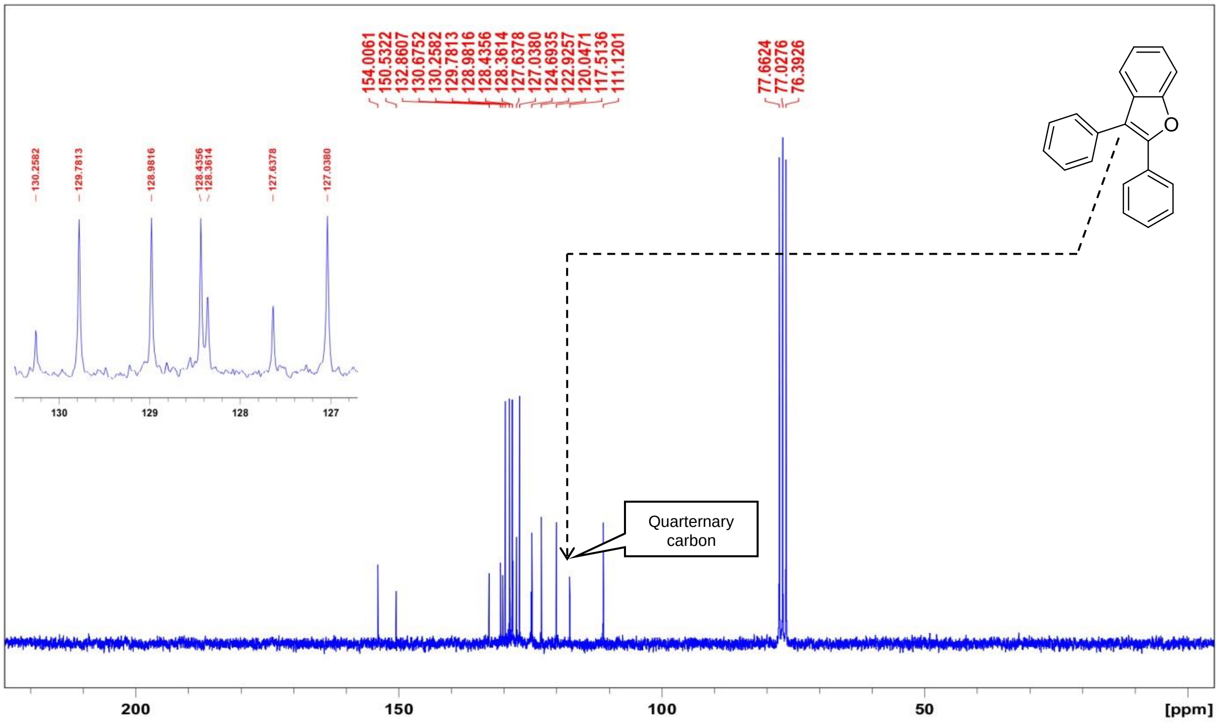

2,3-bis(4-Chlorophenyl)benzo[b]furan (4b) [21]: Synthesized according to the general procedure (in 0.25 mmol scale) as colorless solid (49 mg product, 58% yield); mp 106-108ºC (Lit. 102-104ºC). **^{1}H NMR (200 MHz, $CDCl_3$)** δ (ppm) = 7.59 – 7.54 (m, 3H), 7.49 – 7.40 (m, 6H), 7.37 – 7.29 (m, 3H). **^{13}C NMR (50 MHz, $CDCl_3$)** δ (ppm) = 154.0, 149.6, 134.5, 133.8, 131.0, 131.0(2C), 129.7, 129.4(2C), 129.0, 128.8(2C), 128.2(2C), 125.1, 123.2, 119.8, 116.8, 111.2. **MS analyst, m/z (Int.)**: 340(69), 339(21), 338(100), 302(13), 268(50), 240(13), 239(56), 237(12), 207(10), 151(14), 134(40), 120(16), 119(19).

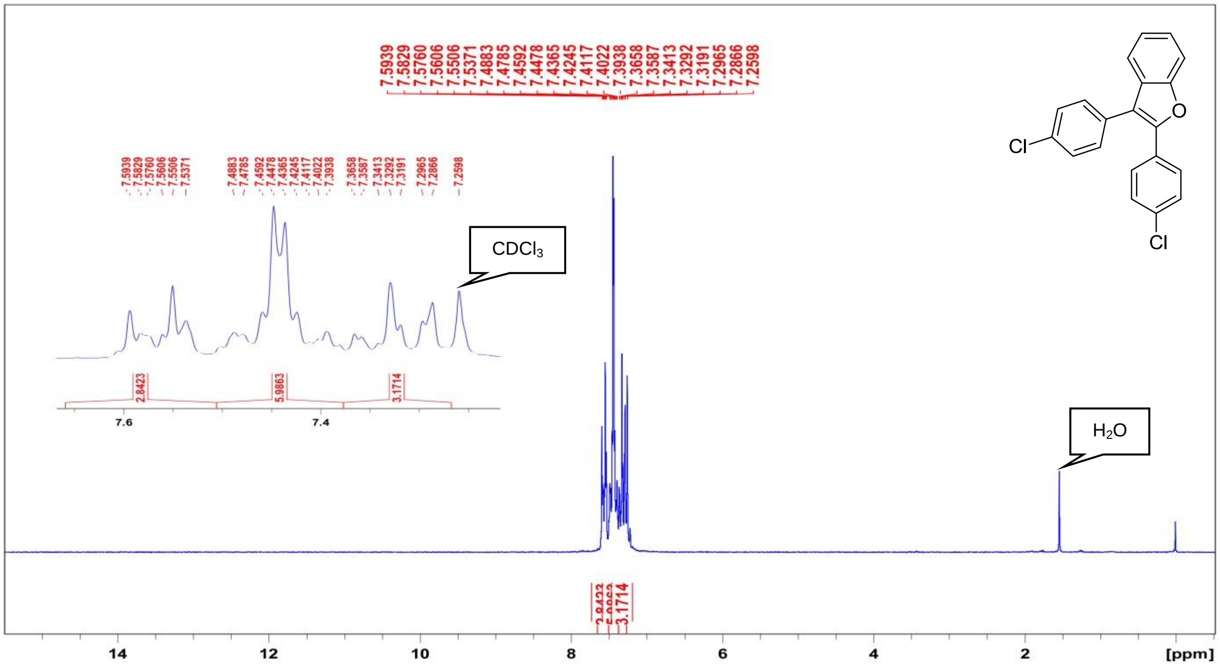

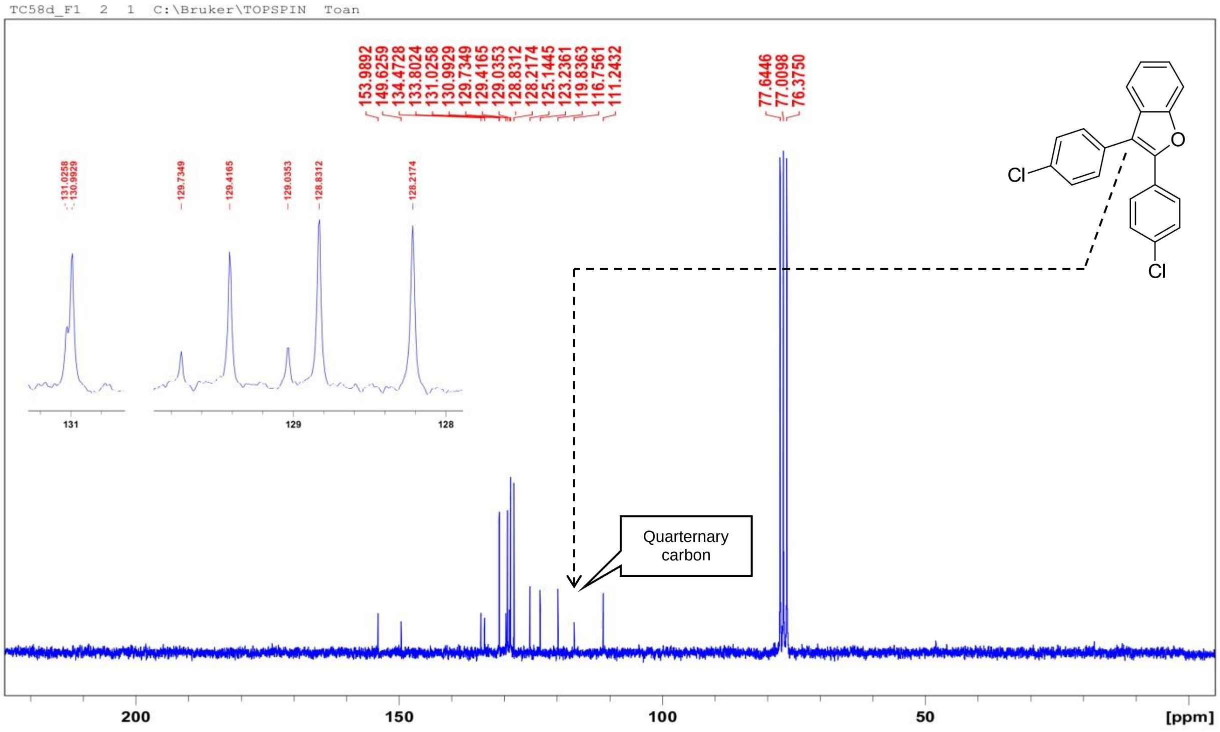

2,3-bis(4-Fluorophenyl)benzo[b]furan (4c) [22]: Synthesized according to the general procedure (in 0.2 mmol scale) as colorless solid (27 mg product, 44% yield); mp 96-98ºC . **^{1}H NMR (200 MHz, $CDCl_3$)** δ (ppm) = 7.65 – 7.53 (m, 3H), 7.49 – 7.13 (m, 7H), 7.07 – 6.98 (m, 2H). **^{13}C NMR (50 MHz, $CDCl_3$)** δ (ppm) = 162.7(d, J_{CF} = 250Hz), 162.4(d, J_{CF} = 250Hz), 153.9, 149.8, 131.4 (d, J_{CF} = 10Hz, 2C), 130.0, 128.9(d, J_{CF} = 10Hz, 2C), 128.5(d, J_{CF} = 3Hz), 126.7(d, J_{CF} = 3Hz), 124.9, 123.1, 119.8, 116.2(d, J_{CF} = 20Hz, 2C), 116.0(d, J_{CF} = 20Hz, 2C), 115.4, 111.2, 103.7. **MS analyst, m/z (Int.)**: 307(21), 306(100), 277(23), 275(17), 257(14), 183(11), 143(11), 129(16).

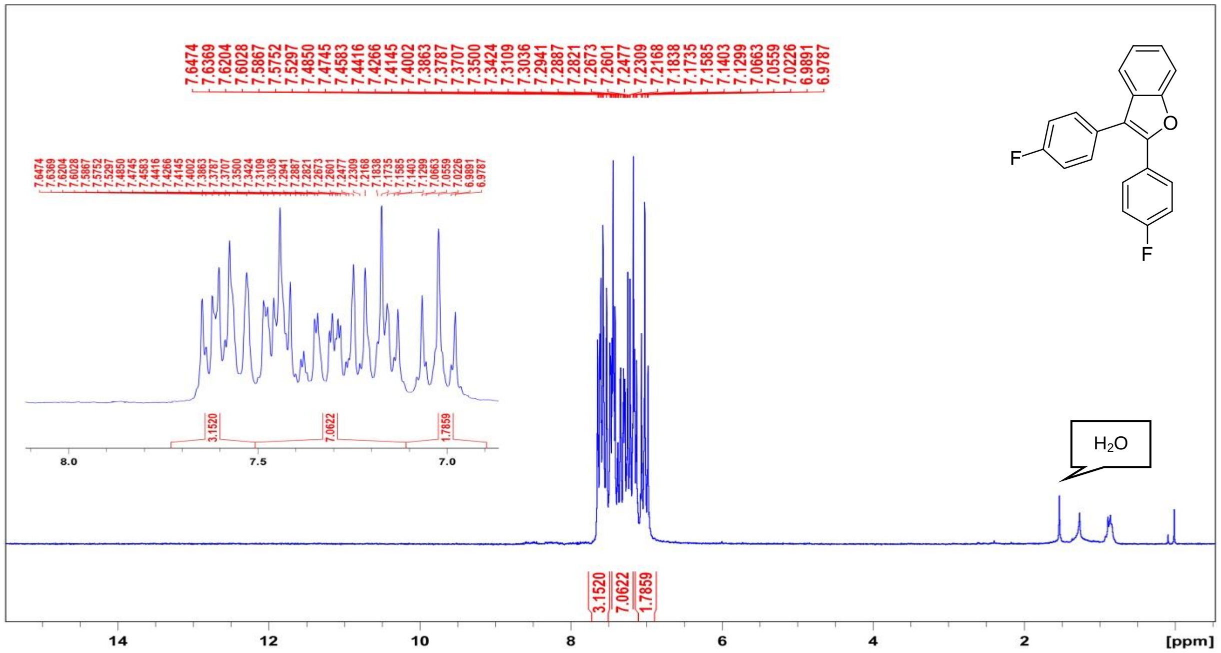

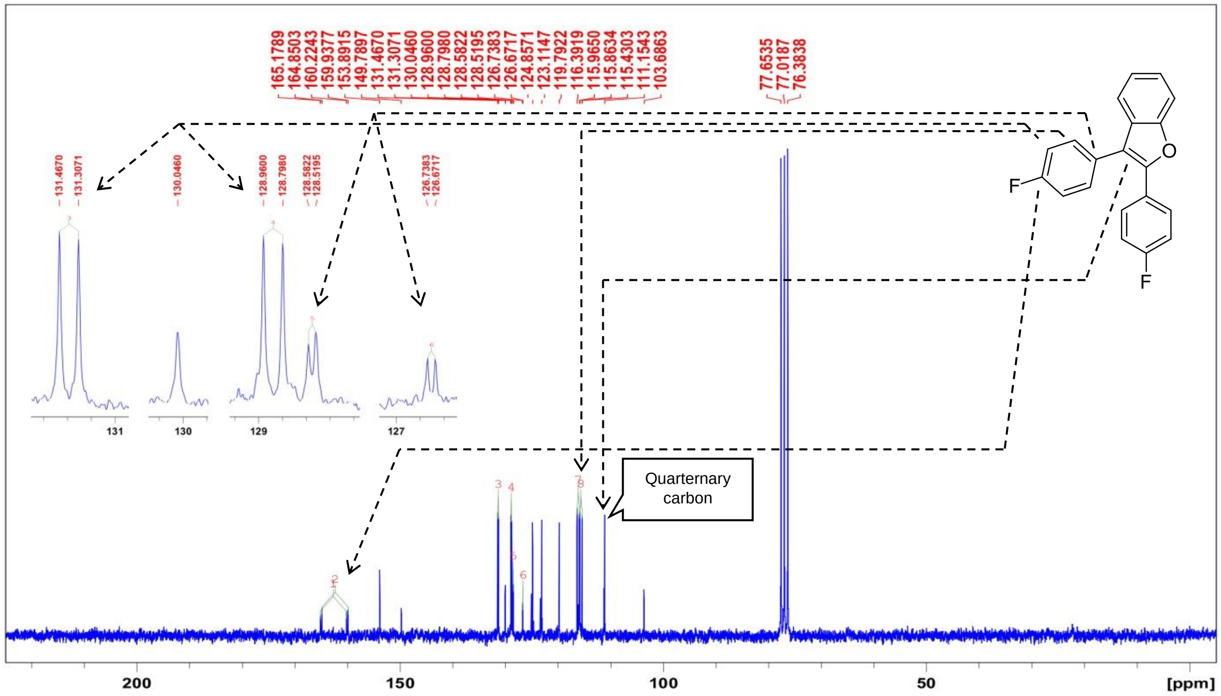

2,3-di-o-Tolylbenzo[b]furan (4d) [22]: Synthesized according to the general procedure (in 0.2 mmol scale) as colorless solid (24 mg product, 40% yield); mp. 71-73ºC. **^{1}H NMR (200 MHz, $CDCl_3$)** δ (ppm) = 7.67 – 7.48 (m, 2H), 7.44 – 7.01 (m, 10H), 2.28 (s, 3H), 2.04 (s, 3H). **^{13}C NMR (50 MHz, $CDCl_3$)** δ (ppm) = 154.3, 152.6, 137.5, 137.1, 131.8, 130.9, 130.7, 130.5, 130.4, 129.7, 128.9(2C), 127.7, 125.9, 125.5, 124.3, 122.7, 120.4, 118.3, 111.2, 20.6, 20.0. **MS Analyst, m/z (Int.)**: 299(100), 298(94), 297(82), 283(16), 256(14), 252(16), 206(16), 192(22), 178(20), 134(24).

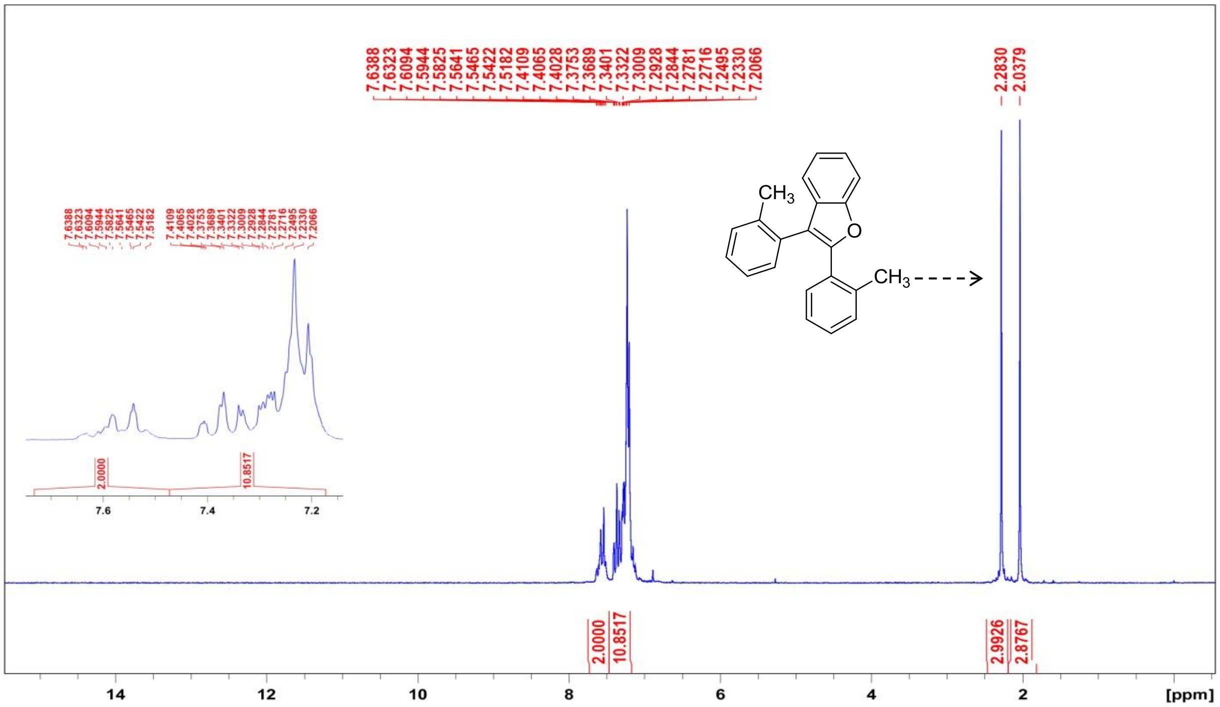

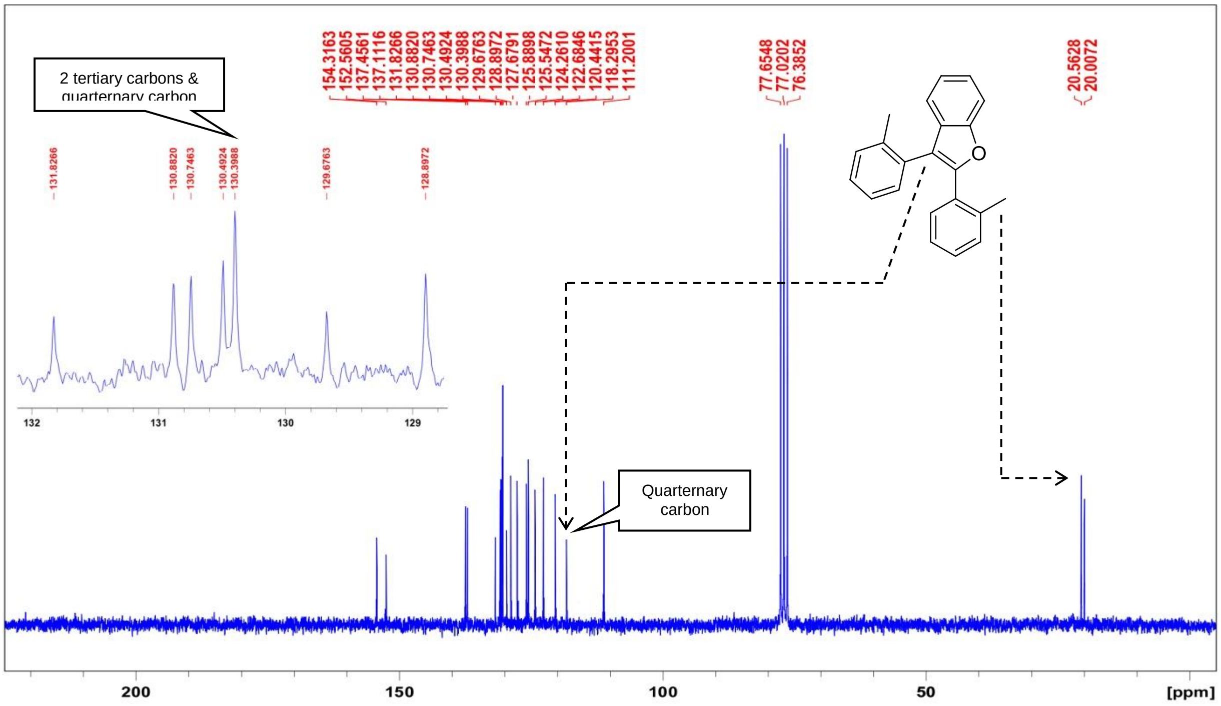

2,3-bis(2-Chlorophenyl)benzo[b]furan (4e) [23]: Synthesized according to the general procedure (in 0.25 mmol scale) as pale yellow solid (45 mg product, 53% yield); mp. 61-64ºC, **^{1}H NMR (200 MHz, CDCl$_3$)** δ (ppm) = 7.65 – 7.61 (m, 1H), 7.53 – 7.41 (m, 5H), 7.37 – 7.23 (m, 6H). **^{13}C NMR (50 MHz, CDCl$_3$)** δ (ppm) = 154.4, 150.4, 134.2, 134.0, 132.3, 132.2, 131.5, 130.4, 130.3, 130.0, 129.9, 129.2, 128.7, 126.9, 126.6, 124.9, 122.9, 121.0, 118.1, 111.5. **MS analyst, m/z (Int.)**: 340(46), 339(14), 338(67), 269(22), 268(100), 240(15), 239(56), 237(19), 134(32), 133(23), 120(29), 119(44), 118(23).

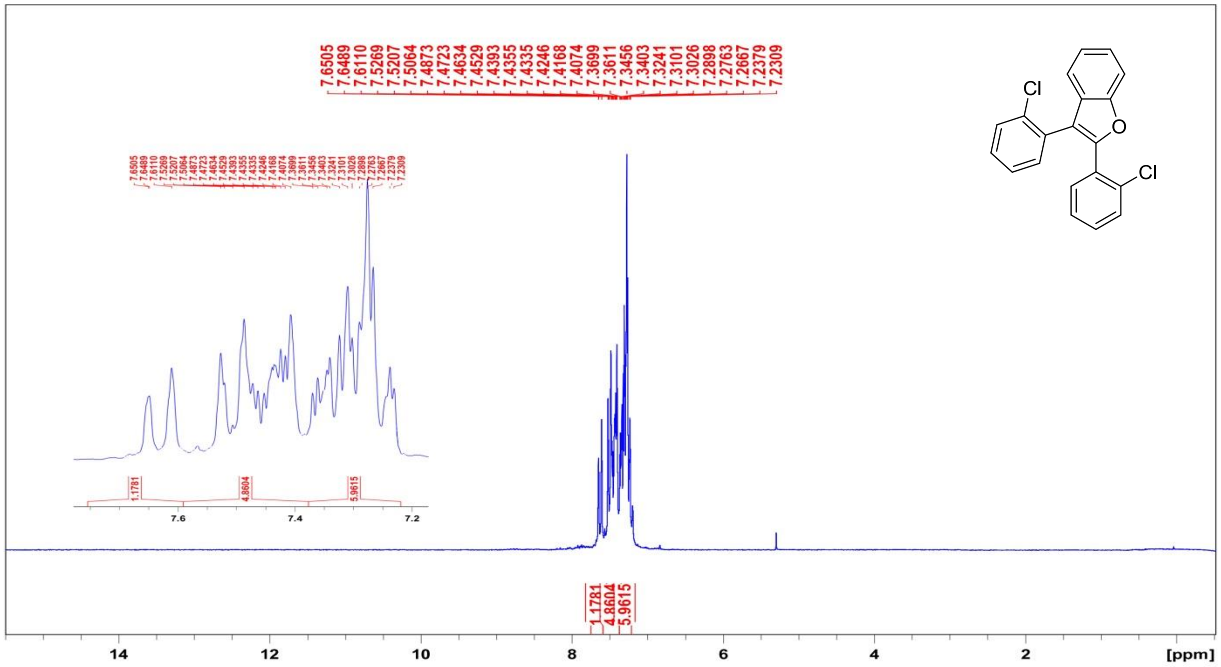

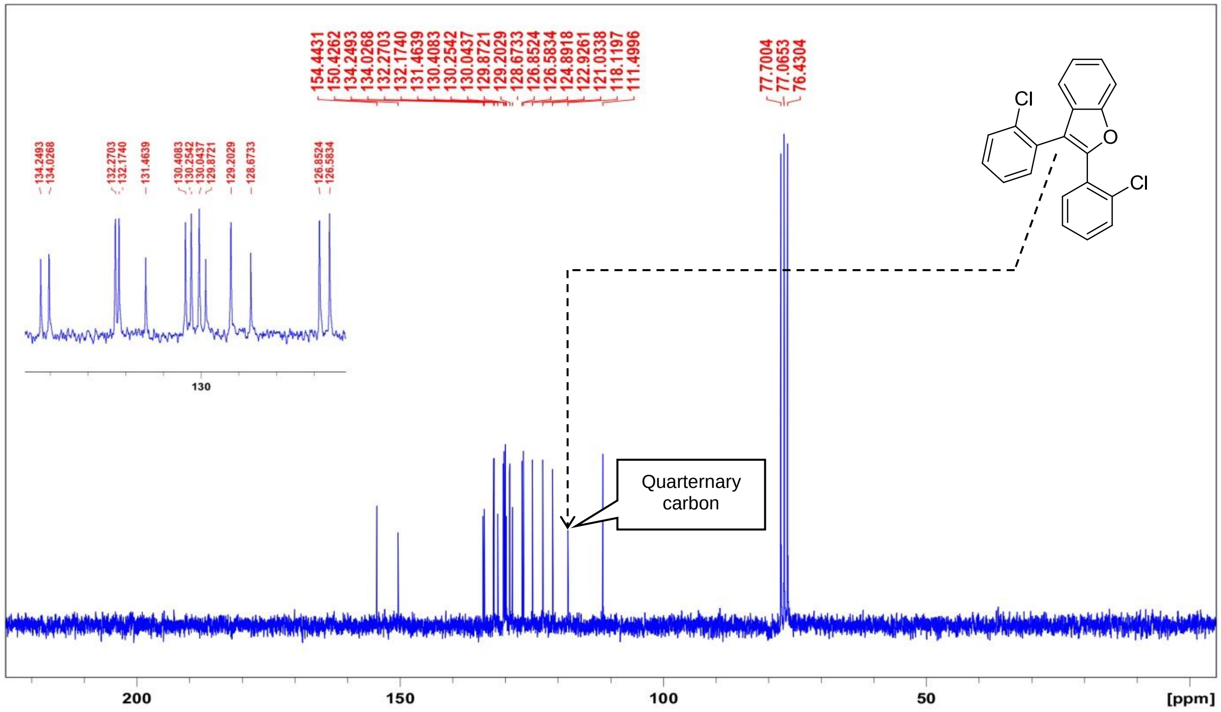

4,4'-(Benzo[b]furan-2,3-diyl)dibenzonitrile (4f) [24]: Synthesized according to the general procedure (in 0.15 mmol scale) as pale yellow solid (8 mg product, 17% yield); mp 178-183ºC. **^{1}H NMR (200 MHz, $CDCl_3$)** δ (ppm) = 7.83 – 7.79 (m, 2H), 7.73 – 7.69 (m, 2H), 7.64 – 7.59 (m, 5H), 7.49 – 7.39 (m, 2H), 7.33 (dd, *J* = 7.4, 1.1 Hz, 1H). **^{13}C NMR (50 MHz, $CDCl_3$)** δ (ppm) = 154.3, 148.9, 137.2, 134.1, 133.1(2C), 132.5(2C), 130.4(2C), 128.9, 127.3(2C), 126.4, 123.9, 120.0, 118.6, 118.5, 118.4, 112.1, 112.1, 111.6. **MS Analyst, m/z (Int.)**: 321(21), 320(100), 291(13), 289(11), 264(12), 206(18), 190(10), 118(12).

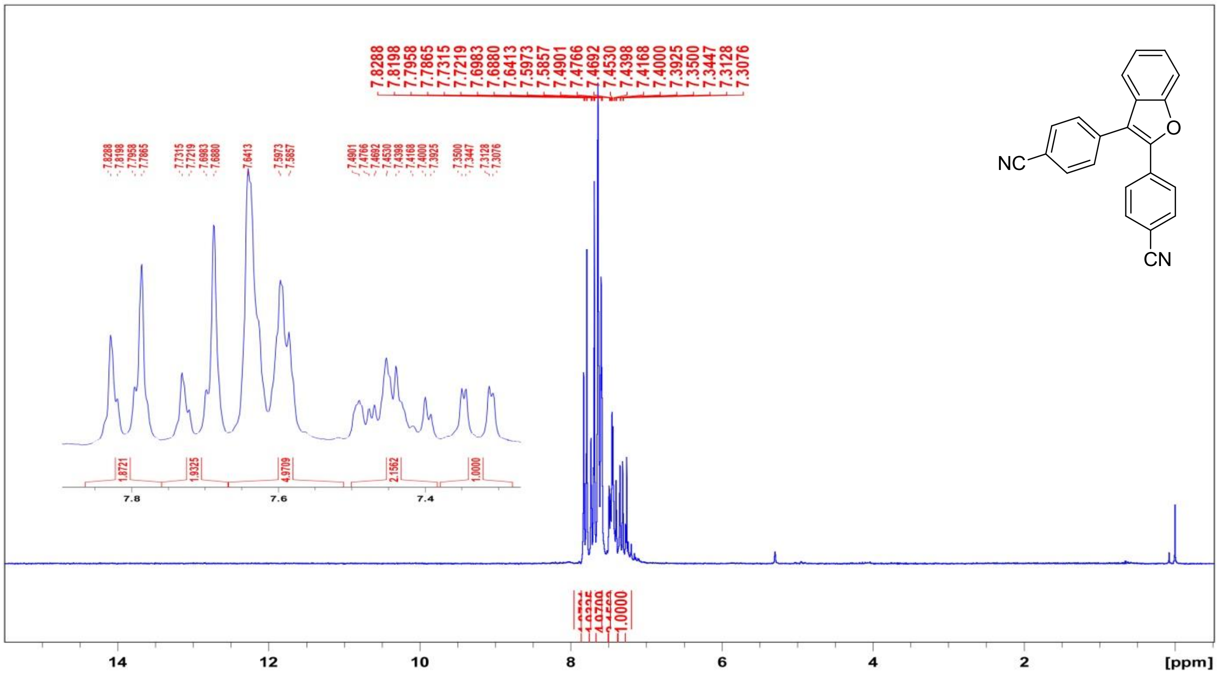

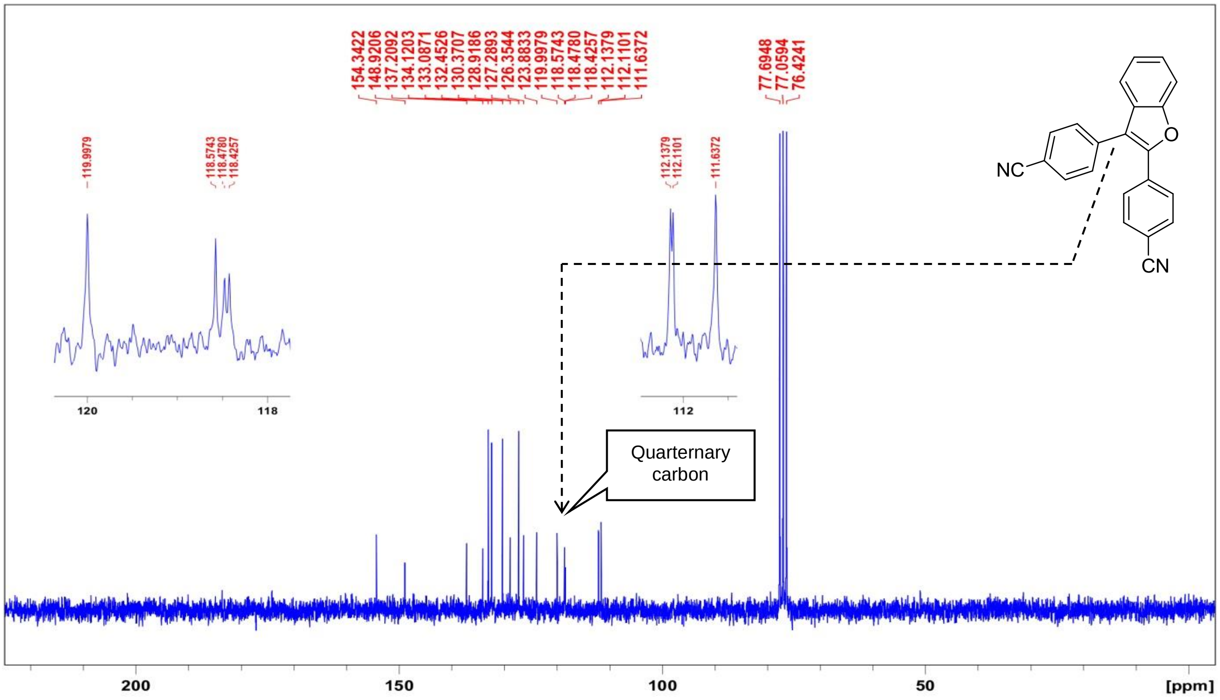

3-(4-Chlorophenyl)-2-phenylbenzo[b]furan (4h) [25]: Synthesized according to the general procedure (in 0.2 mmol scale) as colorless solid (31 mg product, 50% yield). mp 102-103ºC (Lit. 100-101ºC). **^{1}H NMR (200 MHz, CDCl3)** δ (ppm) = 7.66 – 7.59 (m, 2H), 7.59 – 7.46 (m, 2H), 7.45 – 7.43 (m, 4H), 7.38 – 7.22 (m, 5H). **^{13}C NMR (50 MHz, $CDCl_3$)** δ (ppm) = 154.01, 150.75, 133.58, 131.35, 131.09(2C), 130.40, 129.90, 129.29(2C), 128.61, 128.55(2C), 127.09(2C), 124.81, 123.02, 119.72, 116.24, 111.19. **MS analyst, m/z (Int.)**: 306(33), 305(23), 304(100), 269(14), 268(33), 241(29), 240(16), 239(49), 207(33), 135(14), 134(46), 119(46), 118(24).

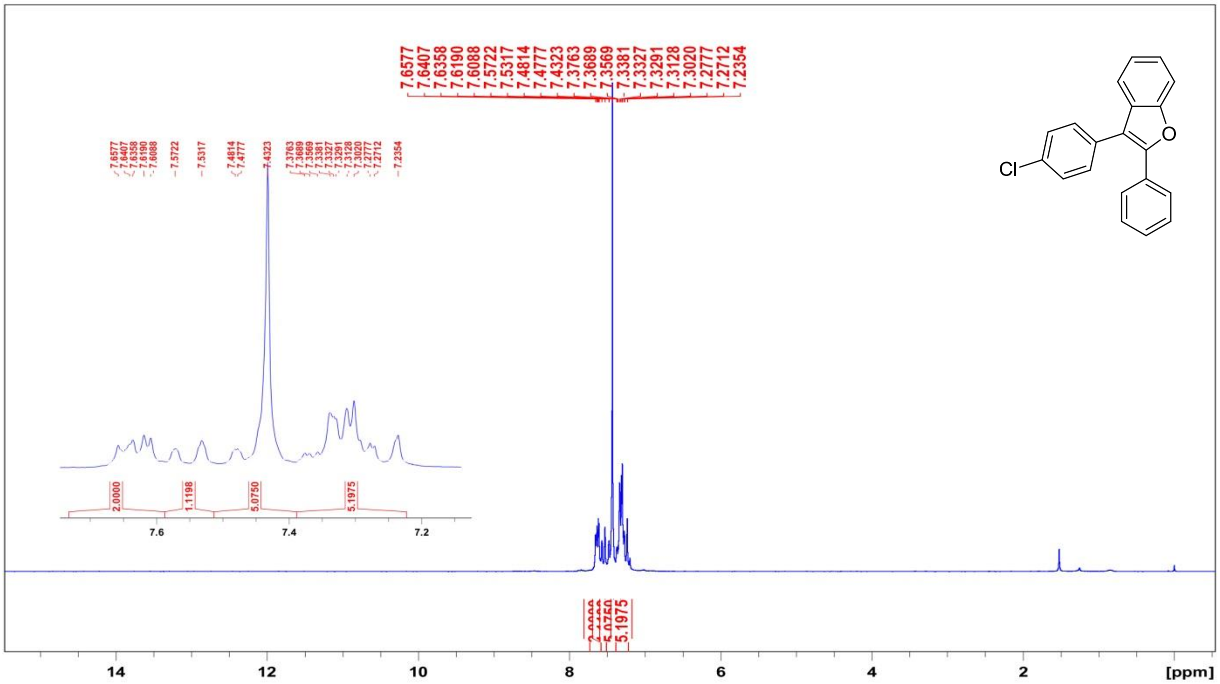

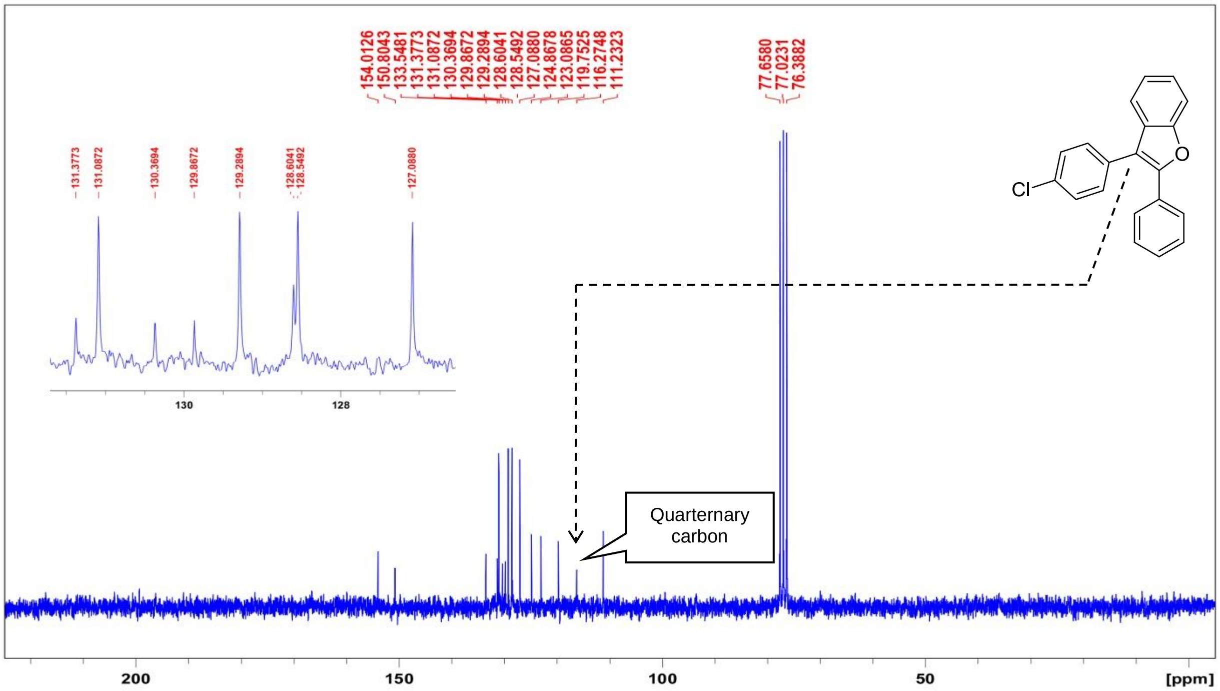

3-(4-nitrophenyl)-2-phenylbenzofuran (4i) [25]: Synthesized according to the general procedure (in 0.2 mmol scale) as colorless solid (13 mg product, 20% yield); mp 140-141ºC (Lit. 135-138ºC). **^{1}H NMR (200 MHz, $CDCl_3$)** δ (ppm) = 8.34 – 8.29 (m, 2H), 7.71 – 7.66 (m, 2H), 7.63 – 7.49 (m, 4H), 7.42 – 7.25 (m, 5H). **^{13}C NMR (50 MHz, $CDCl_3$)** δ (ppm) = 154.19, 151.97, 147.12, 140.24, 130.47(2C), 129.81, 129.21, 128.98, 128.75(2C), 127.46(2C), 125.27, 124.30(2C), 123.49, 119.46, 115.44, 111.50. **No MS analyst**.

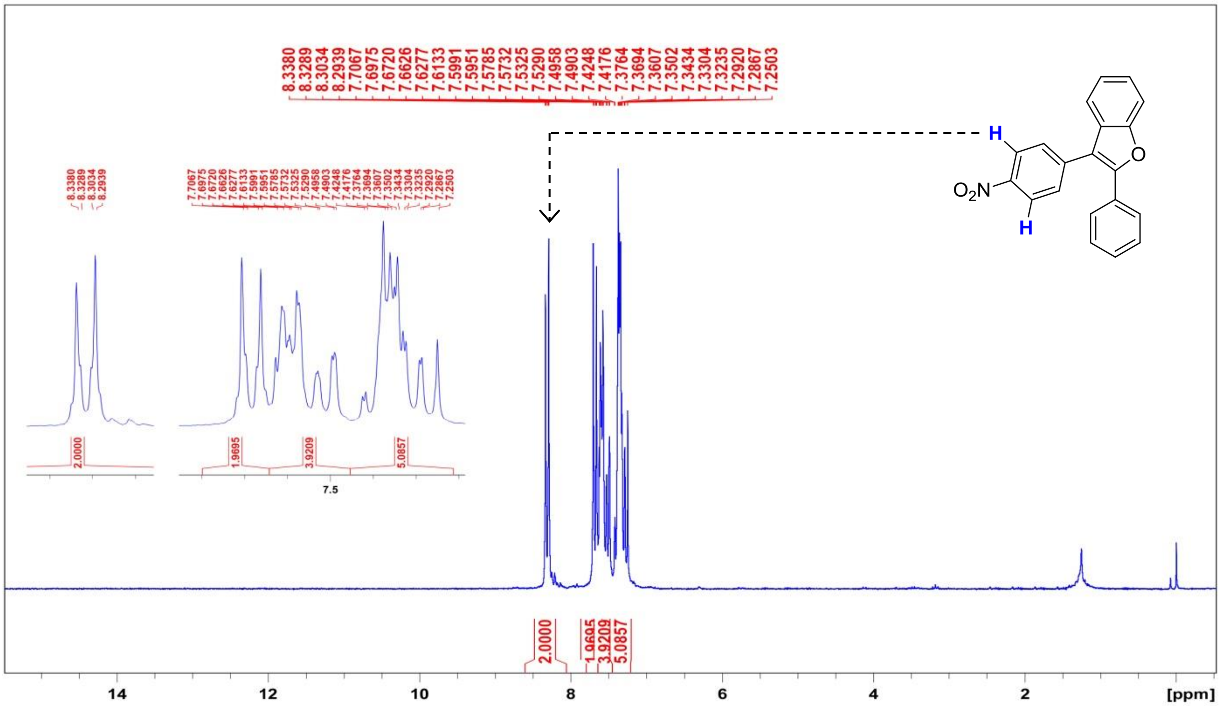

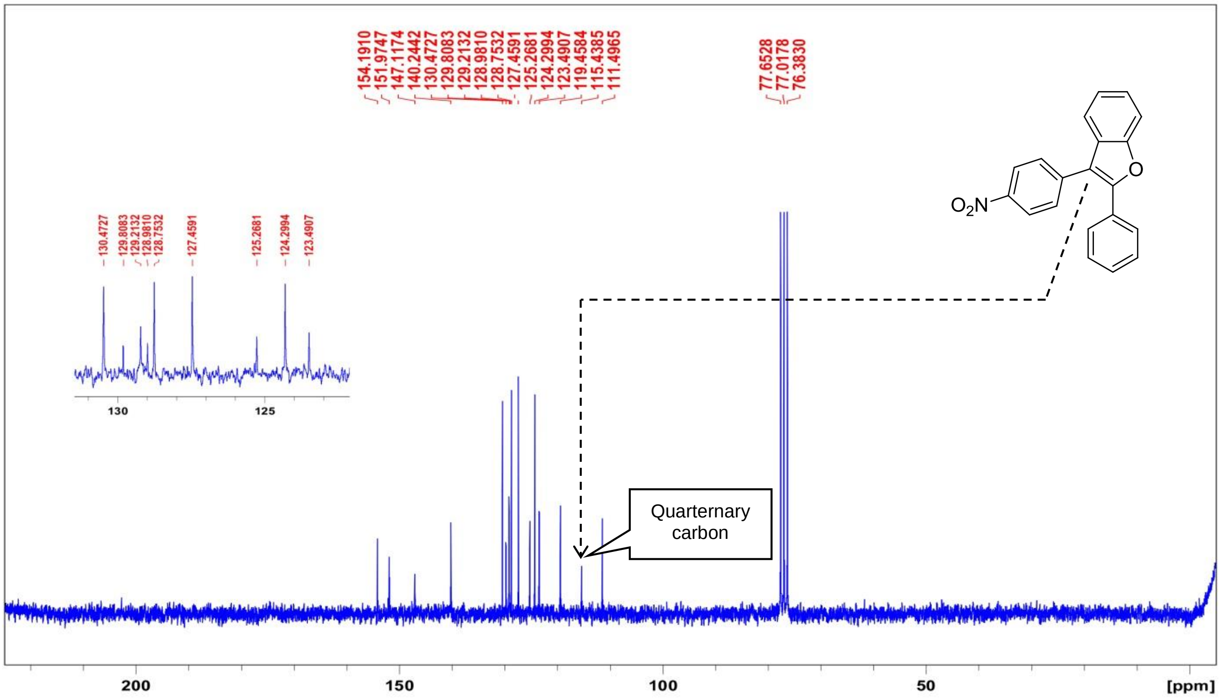

References

[1] L. Ackermann; L. T. Kaspar, *J. Org. Chem.* **2007**, *72*, 6149.

[2] G. M. Kabalka, L. Wang, R. M. Pagni, *Tetrahedron* **2001**, *57*, 8017.

[3] E. A. Jaseer, D. J. C. Prasad, G. Sekar, *Tetrahedron* **2010**, *66*, 2077.

[4] S. Ghosh, J. Das, *Tetrahedron Lett.* **2011**, *52*, 1112.

[5] S. K. Chittimalla, T.-C. Chang, T.-C. Liu, H.-P. Hsieh, C.-C. Liao, *Tetrahedron* **2008**, *64*, 2586.

[6] J. Astoin, P. Demerseman, A. Riveron, R.J. Royer, *Heterocycl. Chem.* **1977**, *14*, 867.

[7] M. Carril, R. S. Martin, I. Tellitu, E. Domínguez, *Org. Lett.* **2006**, *8*, 1467.

[8] X. Wang, M. Liu, L. Xu, Q. Wang, J. Chen, J. Ding, H. Wu, *J. Org. Chem.* **2013**, *78*, 5273−5281.

[9] S. Matsuda, M. Takahashi, D. Monguchi, A. Mori, *Synlett* **2009**, *12*, 1941–1944.

[10] I. Kondolff, H. Doucet, M. Santelli, *J. Heterocyclic Chem.* **2008**, *45*, 109-118.

[11] a) O. Vakuliuk et al , *Adv. Synth. Catal.* **2011**, *353*, 925 – 930; b) S. K. Chittimala, T.-Ch. Chang, T.-Ch. Liu, H.-P. Hsieh, Ch.-Ch. Liao, *Tetrahedron* **2008**, *64*, 2586-3595.

[12] E. K. Yum et al , *Bull. Korean Chem. Soc.* **2013**, *34*, No. 9.

[13] V. Guilarte, M. A. Fernandez-Rodriguez, P. Garcia-Garcia, E. Hernando, R. Sanz, *Org. Lett.* **2011**, *13*, No. 19.

[14] L.-L. Sun, C.-L. Deng, R.-Y. Tang, X.-G. Zhang, *J. Org. Chem.* **2011**, *76*, 7546–7550.

[15] B. Liegault, D. Lapointe, L. Caron, A. Vlassova, K. Fagnou, *J. Org. Chem.* **2009**, *74*, 1826-1834.

[16] A. B. Bíró, A. Kotschy, *Eur. J. Org. Chem.* **2007**, 1364–1368.

[17] T. Truong, M. Mesgar, K. K. A. Le, O. Daugulis, *J. Am. Chem. Soc.* **2014**, *136*, 8568−8576.

[18] M. Baghbanzadeh, C. Pilger, C. O. Kappe, *J. Org. Chem.* **2011**, *76*, 8138-8142.

[19] Y. M. A. Yamada, Y. Yuyama, T. Sato, S. Fujikawa, Y. Uozumi, *Angew. Chem. Int. Ed.* **2014**, *53*, 127 –131.

[20] Y. Terao, T. Satoh, M. Miura, M. Nomura, *Bull. Chem. Soc. Jpn.* **1999**, *72*, 2345–2350.

[21] G. Liu, Y. Shen, Z. Zhou, X. Lu, *Angew. Chem. Int. Ed.* **2013**, *52*, 6033 –6037.

[22] T. H. Nguyen, M. Hussain, I. Malik, A. Villinger, P. Langer, *Tetrahedron Lett.* **2010**, *51(18)*, 2420-2422.

[23] W. Zeng, W. Wu, H. Jiang, L. Huang, Y. Sun, Z. Chen, X. Li, *Chem. Commun.* **2013**, *49*, 6611-6613.

[24] **4,4'-(benzofuran-2,3-diyl)dibenzonitrile (4f),** A 7mL vial equipped with a screw cap with septum and a magnetic stir bar was charged with 4-(benzofuran-2-yl)benzonitrile (**2m**) (110mg, 0.5 mmol), 4-bromobenzonitrile (136mg, 0.75 mmol), cesium pivalate (175mg, 0.75 mmol), $Pd(OAc)_2$ (4.5mg, 0.02mmol) and Sphos (8.2 mg, 0.04 mmol). The vial was closed, evacuated and flushed with argon 3 times. Then 2 mL of degassed DMAc were added by syringe. The mixture was heated to 140 ºC for 24 hours. The reaction mixture was cooled to room temperature and then diluted with 15mL ethylacetate and filtered through a pad of celite. The organic phase was washed 3 times with saturated ammonium chloride solution, once with brine and dried over sodium sulfate. The solvent was removed under reduced pressure. Purification was performed by MPLC

on silica gel with gradient in polarity of solvent mixture (PE → PE/EtOAc 95:5) to afford 27mg (34%) **4f** as pale yellow solid. Mp. 178-183ºC. ^{1}H NMR (200 MHz, $CDCl_3$) δ (ppm) = 7.83 – 7.79 (m, 2H), 7.73 – 7.69 (m, 2H), 7.64 – 7.59 (m, 5H), 7.49 – 7.39 (m, 2H), 7.33 (dd, J = 7.4, 1.1 Hz, 1H). ^{13}C NMR (50 MHz, $CDCl_3$) δ (ppm) = 154.3, 148.9, 137.2, 134.1, 133.1(2C), 132.5(2C), 130.4(2C), 128.9, 127.3(2C), 126.4, 123.9, 120.0, 118.6, 118.5, 118.4, 112.1, 112.1, 111.6. MS Analyst, m/z (Int.): 321(21), 320(M^+, 100), 291(13), 289(11), 264(12), 206(18), 190(10), 118(12).

[25] N. A. Markina, Y. Chen, R. C. Larock, *Tetrahedron* **2013**, *69*, 2701-2713.
